# A novel diterpene agent isolated from *Microbispora hainanensis* strain CSR-4 and its in vitro and in silico inhibition effects on acetylcholine esterase enzyme

**DOI:** 10.1038/s41598-020-68009-y

**Published:** 2020-07-06

**Authors:** Chitti Thawai, Nantiya Bunbamrung, Pattama Pittayakhajonwut, Sumet Chongruchiroj, Jaturong Pratuangdejkul, Ya-Wen He, Sarin Tadtong, Vipaporn Sareedenchai, Pinidphon Prombutara, Yang Qian

**Affiliations:** 1 Department of Biology, Faculty of Science, King Mongkut’s Institute of Technology Ladkrabang, Bangkok, 10520 Thailand; 2 Actinobacterial Research Unit, Faculty of Science, King Mongkut’s Institute of Technology Ladkrabang, Bangkok, 10520 Thailand; 3 Center of Excellence in Applied Biosciences, King Mongkut’s Institute of Technology Ladkrabang, Bangkok, 10520 Thailand; 4 National Center for Genetic Engineering and Biotechnology (BIOTEC), National Science and Technology Development Agency (NSTDA), Thailand Science Park, Phaholyothin Road, Klong Luang, 12120 Pathum Thani Thailand; 5 Department of Microbiology, Faculty of Pharmacy, Mahidol University, Phayathai, Bangkok, 10400 Thailand; 6 State Key Laboratory of Microbial Metabolism, School of Life Sciences and Biotechnology, Shanghai Jiao Tong University, Shanghai, 200240 People’s Republic of China; 7 Department of Pharmacognosy, Faculty of Pharmacy, Srinakharinwirot University, Ongkarak, 26120 Nakhon nayok Thailand; 8 Omics Sciences and Bioinformatics Center, Faculty of Science, Chulalongkorn University, 254 Payathai Road, Patumwan, Bangkok, 10330 Thailand; 9 Department of Life Science and Engineering, Harbin Institute of Technology, Harbin, 150001 People’s Republic of China

**Keywords:** Biotechnology, Chemical biology, Computational biology and bioinformatics, Drug discovery, Microbiology, Chemistry

## Abstract

An actinomycete strain CSR-4 was isolated from the rhizosphere soil of *Zingiber montanum.* Taxonomic characterization revealed strain CSR-4 was a member of the genus *Microbispora*. Whole-genome sequence analysis exhibited the highest average nucleotide identity (ANI) value (95.34%) and digital DNA–DNA hybridization (DDH) value (74.7%) between strain CSR-4 and the closest relative *M. hainanensis* DSM 45428^T^, which was in line with the assignment to same species. In addition, a new diterpene compound, 2α-hydroxy-8(14), 15-pimaradien-17, 18-dioic acid, and nine known compounds were isolated from the ethyl acetate crude extract of fermentation broth. Interestingly, a new diterpene displayed the suppressive effect on the recombinant human acetylcholinesterase (rhAChE) enzymes (IC_50_ 96.87 ± 2.31 μg/ml). In silico studies based on molecular docking and molecular dynamics (MD) simulations were performed to predict a binding mode of the new compound into the binding pocket of the rhAChE enzyme and revealed that some amino acids in the peripheral anions site (PAS), anionic subsite, oxyanion site and catalytic active site (CAS) of the rhAChE have interacted with the compound. Therefore, our new compound could be proposed as a potential active human AChE inhibitor. Moreover, the new compound can protect significantly the neuron cells (% neuron viability = 88.56 ± 5.19%) from oxidative stress induced by serum deprivation method at 1 ng/ml without both neurotoxicities on murine P19-derived neuron cells and cytotoxicity against Vero cells.

## Introduction

Actinomycetes have been considered as an important microbial source for the biotechnological and ecological roles^1^. Genus *Microbispora*, the important microorganism of the family *Streptosporangiaceae* in the class actinobacteria, has been reported to be involved in the biodegradation process that provides humus, plant nutrients and suppresses the growth of plant pathogens by secreting the bioactive secondary metabolites to the soil^2^. Many studies revealed that members in the genus *Microbispora* are the excellent secondary metabolite producers as they are able to provide a large number of promising bioactive compounds such as antibiotics, enzymatic inhibitors, antidiabetic and antiatherogenic compounds^3–5^. Recently, it was estimated that the world’s population of elderly (aged 60 years or older) will grow rapidly in near decades, above 16% of the total population^6^. One of the major health problems related to age is a cognitive dysfunction, which includes neuropsychiatric and neurodegenerative disorders, such as Alzheimer’s disease (AD), schizophrenia, depression, and Parkinson’s disease^6–8^. It is known that acetylcholinesterase (AChE) is a cholinergic enzyme that catalyzes the breakdown of acetylcholine (ACh), an important neurotransmitter in the brain. The reduction of ACh is the major cause of AD^7,8^. The promising way for treating these problems is to enhance the acetylcholine (ACh) level in the brain. Microorganisms especially actinomycetes have been reported as promising sources for anti-AChE agents. Geranylphenazinediol was isolated from marine *Streptomyces* sp. LB173 showing AChE inhibitory activity at the low concentration (micromolar range)^9^. *Rubrobacter radiotolerans* is an outstanding marine actinomycete that produces rare dimeric indole derivatives. These indole derivatives showed the AChE inhibitory activity with IC_50_ values of 11.8–13.5 μM^10^. The extract from the fermentation broth of a marine-derived *Streptomyces* sp. UTMC 1334 exhibited a moderate inhibition of AChE (IC_50_ = 0.36 ± 0.019 mg ml^−1^)^11^. *S. parvulus* strain Tu 64 is a potential actinomycete that produces Manumycin C, B, and A. These compounds inhibited the AChE with IC_50_ of 15.0, 11.5, 12.5 mM, respectively^12^. Moreover, many compounds obtained from actinomycetes have been documented for having neuroprotective properties. For example, a rare molecule with β-carboline chromophore named mescengricin was isolated from *S. griseoflavus* 2853-SVS4 and affected the reduction of the l-glutamate toxicity in chick primary mescencephalic neurons with an EC_50_ value of 6.0 nM^13^. 1,4-Naphtoquinone derivative, flaviogeranin, was isolated from *Streptomyces* sp. RAC226 and possessed neuroprotective activity by preventing cell death in C6 cells treated with 100 nM of glutamate for 24 h with an EC_50_ of 8.6 nM^14^. Naphthomycinal was isolated from *Streptomyces* sp. PF7 and decreased the l-glutamate toxicity in N18-RE-105 cells with EC_50_ value of 400 nM^15^. Due to no suppression of the buthionine sulfoximine (BSO) toxicity, compared with vitamin E, the mode of action of this compound should not be involved in the anti-oxidative activity. l-Glutamate toxicity in N18-RE-105 cells is caused by suppression of the synthesis of the intracellular reducing agent glutathione. Bicyclohexapeptides, named neuroprotectins A and B, were isolated from the culture broth of *Streptomyces* sp. Q27107 and effectively protected the primary cultured chick telencephalic cells from glutamate- and kainite-induced neurotoxicities^16^. Indanostalin was isolated from *Streptomyces* sp. and exhibited neuroprotective activity against C6 rat glioma cells at EC_50_ value of 130 nM^17^. It showed no protective activity on N18-RE-105 rat primary retina-mouse neuroblastoma hybrid cells at less than 40 mM. The carbazole derivatives with an *ortho* quinone moiety, named carquinostatin and lavanduquinocin, were isolated from *S. exfoliates*^18^ and *S. viridochromogenes*^19^, respectively. Both compounds reduced l-glutamate toxicity in neuronal hybridoma N18-RE-105 cells with EC_50_ values of 15.5 nM and 4.3 nM, respectively. In the case of the secondary metabolites produced from *Microbispora*, only a few compounds have been reported for having biological property e.g. bispolides, 20-membered ring macrodiolide antibiotics, were extracted and purified from the fermentation of *Microbispora* sp. A34030. The compounds exhibited an anti-MRSA activity^4^. A furanone-containing polyketide, linfuranone A, was produced by an endophytic *Microbispora* sp. GMKU363 isolated from a Thai medicinal plant *Clinacanthus siamensis* Bremek. Linfuranone A displays antidiabetic and antiatherogenic activities^5^. Thus, an attempt to discover the new natural product to inhibit the acetylcholinesterase enzyme is still highlighted. Since the crude extracted from the fermentation broth of *Microbispora* sp. CSR-4 showed anti-acetylcholinesterase (anti-AChE) activities, further investigation on bioactive ingredient was conducted and led to the isolation of a new diterpene. Herein, we report the chemical characterization of the new diterpenoid, 2α-hydroxy-8(14),15-pimaradien-17, 18-dioic acid together with its both in vitro and in silico anti-AChE activities. Additionally, the neuroprotective, cytotoxic, and antioxidant activities of the new compound are also carried out. Moreover, the taxonomic characterizations of strain CSR-4 are also performed.

## Results and discussion

### Taxonomic characterization of strain CSR-4

*Zingiber cassumunar* Roxb. is a famous medicinal plant in Thailand. The rhizome of this plant has usually been used for the treatment of muscle and skin problems. Many reports revealed that the rhizome of *Z. cassumunar* comprises several kinds of volatile oils. Furthermore, the bioactive secondary metabolites of the extract of *Z. cassumunar* rhizome have been reported to inhibit the growth of pathogenic microorganisms^20^. Thus, the actinomycete living around the rhizome is unique in its adaptations to the chemical environment of a host plant and is considered to be a potential source for bioactive natural products^21,22^. Strain CSR-4 was isolated from the rhizosphere soil of *Z. cassumunar* rhizome. Cultural characteristic results revealed that strain CSR-4 showed good growth on ISP2 and ISP3 agars, but moderate to poor growth on ISP4, ISP5, ISP6, ISP7, glucose asparagine, Czapek sucrose, and nutrient media. The color of substrate mycelium was light yellowish-brown to strong yellowish-brown on the different media tested. Greyish white to white aerial mycelium could be observed on ISP2 and ISP3 media after 14 days of cultivation at 30 °C (Additional files: Fig. S4a). The pale yellow diffusible pigment was detected on both media tested. Strain CSR-4 produced longitudinally paired spores (0.8–1.0 × 0.9–1.2 µm) borne directly on aerial mycelia (Additional files: Fig. S4b). Spore arrangement of strain CSR-4 shows typical morphology found in the genus *Microbispora*^23^. Strain CSR-4 was Gram-stain-positive, mesophilic, catalase, and oxidase-positive actinomycete. Nitrate reduction and urease activity were positive. Peptonization of milk and hydrolysis of starch were negative. Like *M. hainanensis* DSM 45428^T^, strain CSR-4 grew in the presence of up to 1% (w/v) NaCl. The pH and temperature for cell growth were in the range of pH 5–10 and 20–50 °C. The results obtained from API ZYM test revealed that both strains were able to produce alkaline phosphatase, esterase C4, esterase lipase C8, leucine arylamidase, valine arylamidase, trypsin, α-chymotrypsin, acid phosphatase, naphtol phosphohydrolase, α-glucosidase, β-glucosidase, *N*-acetyl-β-glucosaminidase but the production of lipase (C14), β-glucuronidase, α-mammosidase, and α-fucosidase was not observed. Only a few biochemical characteristics i.e. the production of α-galactosidase, β-galactosidase, cysteine arylamidase, the inability to decompose hypoxanthine and starch hydrolysis were observed as the phenotypically different points between both strains. Other physiological and biochemical characteristics of strains CSR-4 and *M. hainanensis* DSM 45428^T^ were shown in Additional files (Table S1).

**Figure 1:**
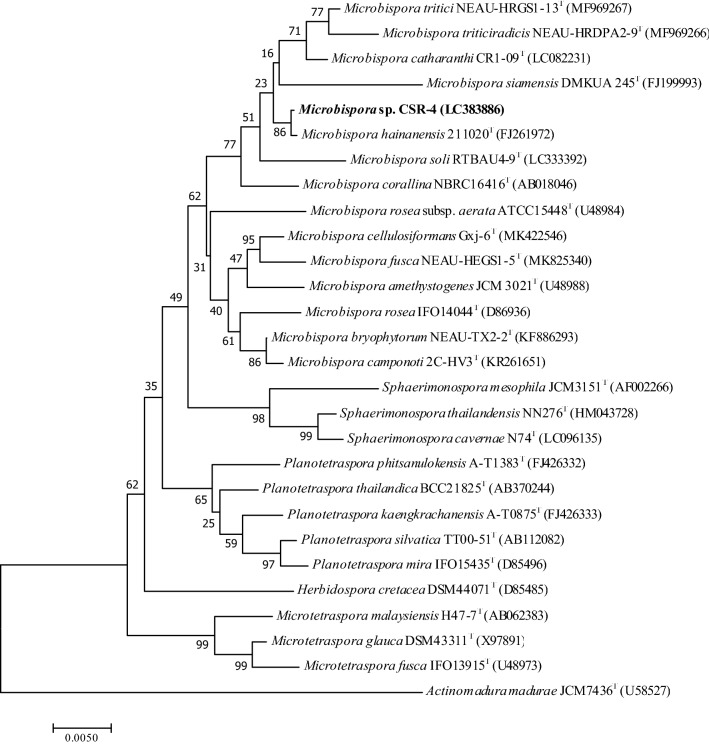
Neighbour-joining phylogenetic tree based on 16S rRNA gene sequences comparing strain CSR-4 to *Microbispora* species and other genera in the family *Streptosporangiaceae*. *Actinomadura madurae* JCM 7436^T^ was used as the out-group. The numbers on the branches indicate the percentage bootstrap values of 1,000 replicates.

The chemotaxonomic characteristics of strain CSR-4 were consistent with the assignment to the genus *Microbispora*. The strain contained meso-diaminopimelic acid in its cell-wall peptidoglycan. The diagnostic reducing sugar, madurose (3-*O*-methyl-d-galactose), was found in the cell hydrolysates. Similar results were observed in all type strains of the genus *Microbispora* e.g. *M. bryophytorum*^24^, *M. camponoti*^25^, *M. corallina*^26^, *M. siamensis*^27^, and *M. rosea*^28^, with the exception of *M. hainanensis*^29^. Besides, glucose and ribose were reducing sugars observed in the whole-cell hydrolysates of strain CSR-4. The characteristic menaquinones, MK-9(H_2_), MK-9(H_0_), MK-9(H_4_), were detected in the cell of strain CSR-4. Similarly, the closest relative, *M. hainanensis*, also contained MK-9(H_4_), MK-9(H_2_), and MK-9(H_0_) in different proportions. The polar lipid profile of strain CSR-4 contains phosphatidylethanolamine, phosphatidylmethylethanolamine diphosphatidylglycerol, phosphatidylinositol, two ninhydrin-positive phosphoglycolipids, and one unidentified phospholipid. In the case of fatty acid analysis, strain CSR-4 exhibited the same major fatty acid components (> 10%), iso-C_16:0_ and C_16:0_ that was also found in *M. hainanensis* (Additional files: Table S2). Interestingly, three common fatty acids, iso-C_16:0_, C_16:0_, and 10-methyl C_17:0_ were found to have in all members of the genus *Microbispora*, but *M. siamensis*^16^ and *M. soli*^30^ were excluded. The morphological and chemotaxonomic data confirmed the taxonomic affiliation in the genus level that strain CSR-4 belongs to the genus *Microbispora*. 16S ribosomal RNA gene-based phylogenetic analysis showed that strain CSR-4 clustered clearly with *M. hainanensis* supported by a high bootstrap value of 86% and 77% in the neighbor-joining^31^ and maximum-likelihood^32^ trees, respectively (Fig. 1, Additional files: Fig. S1). The phylogenomic tree also showed that strain CSR-4 formed the same phyletic branch with *M. hainanensis* (Additional files: Fig. S2). The EzBioCloud analysis^33^ revealed that a complete 16S rRNA gene sequence (1,498 nt) of strain CSR-4 shared the highest 16S rRNA gene sequence similarities to *M. hainanensis* 211020^T^ (99.9% 16S rRNA gene sequence similarity) followed by *M. rosea* subsp. *rosea* ATCC 12950^T^ (98.8%) and *M. corallina* DF-32^T^ (98.7%). According to the draft genome sequence, the genomic DNA G + C content of this strain was 71.3 mol%, which was close to those of the type strains of species of the genus *Microbispora*^23–29^. The genotypic data showed that strain CSR-4 exhibited the highest 16S rRNA gene sequence similarity to *M. hainanensis* 211020^T^. Additionally, the average nucleotide identity (ANI) values of the draft genomic sequence obtained from two online servers, Kostas Lab^34^ and JspeciesWS^35^, were 95.34% and 95.06, respectively, a result well above the threshold used to delineate prokaryote species^36^ (Additional files: Table S3). Moreover, the digital DNA–DNA hybridization (dDDH)^37^ value between the genomes of the two strains was 74.7%, which was higher than the 70% cut-off point for assigning strains to the same species suggested by Wayne et al.^38^. In consistent with its phenotypic, chemotaxonomic characteristics, and genome characterization, strain CSR-4 was a member of the species *M. hainanensis*.

### Genome analysis for secondary metabolites and bioinformatic detail of strain CSR-4

De novo assembly of the genome sequencing data of strain CSR-4 resulted in 440 contigs, an N50 of 113 kb, and genome coverage of 102 ×. The draft genome of strain CSR-4 was 8,679,128 bp in total with a 71.3 mol% G + C, and was deposited to GenBank with the accession number VJVX00000000 (Additional files: Table S4). Of the 7,910 genes predicted, 86 of them were assigned to RNA genes (76, 9, and 1 were predicted as tRNA, rRNA, and tmRNA genes, respectively). Besides, 7,824 genes were identified as protein-coding sequences. Functional analysis by clusters of orthologous genes (COGs) revealed that the most of predicted CDS (> 50%) are involved in metabolisms (28.3% protein metabolism, 16.0% carbohydrate metabolism, 7.7% lipid metabolism, 4.8% nucleotides and nucleosides metabolism). The other genes are responsible for the cellular processes and information storage and processing. The poorly characterized genes are also observed (Additional files: Fig. S5). Further analysis using Artermis Comparison Tool (ACT)^39^ which uses BLAST to compare two genomes revealed a small amount of synteny exists between strain CSR-4 and *M. hainanensis* DSM 45428^T^ (Additional files: Fig. S3). Moreover, the results obtained from the antiSMASH server^40^ revealed that strain CSR-4 contained six terpene biosynthesis gene clusters, type I polyketide synthase (T1PKS) genes, type III polyketide synthase (T3PKS) genes, and several non-ribosomal peptide synthetase (NRPS) genes (Additional files: Table S5). Others were associated with the synthesis of phenazines and siderophores. Gene clusters found in strain CSR-4 include those related to the production of colabomycin E, frankiamicin, abyssomicins M-X, acarviostatin, octacosamicin, showdomycin, streptovaricin, tetronasin, quartromicin, cyclizidine. In case of secondary metabolism biosynthetic gene clusters (smBGCs) analyzed by using antiSMASH server^40^, the genome of strain CSR-4 contains gene clusters exhibiting relatedness to the cluster known to produce alkylresorcinol (100%) in *Streptomyces griseus* subsp. *griseus* NBRC 13350^T^ (MIBiG accession BGC0000282, GenBank accession AP009493.1, positions 548,718–551,330)^41^, together with desferrioxamine (83%) in *Streptomyces argillaceus* (MIBiG accession BGC0001453, GenBank accession LT989883.1, positions 1–7,234)^42^ and guangnanmycin (71%) in *Streptomyces* sp. CB01883 (MIBiG accession BGC0001611, GenBank accession MF925481.1, positions 1–72,893)^43^. One of the terpene biosynthesis genes found in strain CSR-4 had 100% similarity to the geosmin biosynthetic gene cluster from *Nostoc punctiforme* PCC 73102 (MIBiG accession BGC0000661, GenBank accession CP001037.1, positions 3,414,766–3,420,177)^44^. It is well known that geosmin can be produced from soil microorganisms especially actinomycetes^45^. Remaining terpene biosynthesis genes of strain CSR-4 revealed a low similarity percentage (< 38%) to the hopene biosynthetic gene cluster from *Streptomyces coelicolor* A3 (MIBiG accession BGC0000663, GenBank accession AL645882.2, positions 7,516,017–7,529,773). Furthermore, there are six smBGCs in the genome of strain CSR-4, including linear azol(in)e-containing peptides (LAP) (positions 117,181–140,997 nt.), siderophore (positions 23,071–36,328 nt.), bacteriocin (positions 20,923–31,078 nt.), betalactone (positions 41,961–74,800 nt.), NRPS-like fragment (positions 30,117–74,139 nt.), and terpene (positions 50,061–71,761 nt.) clusters, showing no relatedness to known cluster in antiSMASH analysis. This implied a high opportunity of strain CSR-4 in producing new terpene products and suggested its ability for valuable bioactive compound production.

### Structure elucidation of the isolated compounds

**Figure 2:**
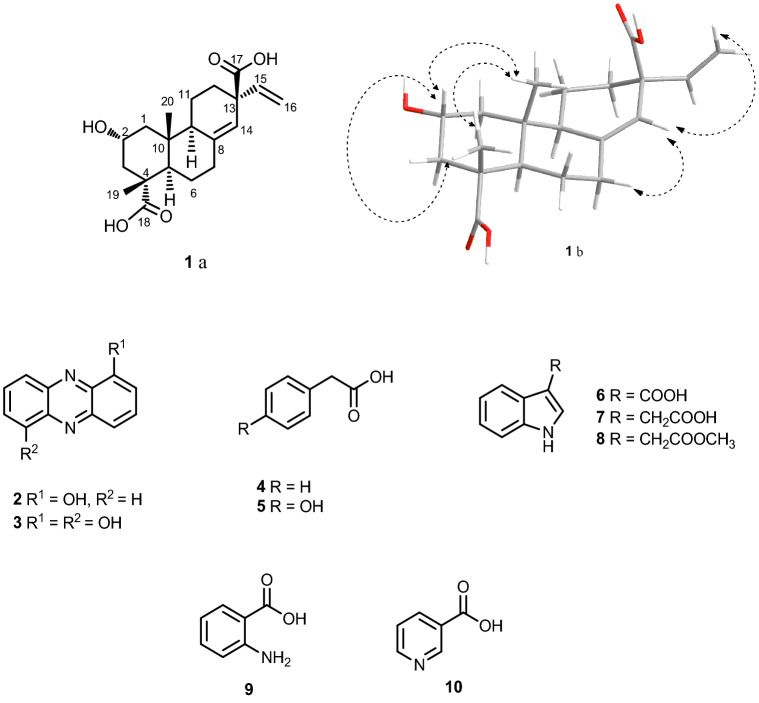
Chemical structures of compounds **1**–**10** isolated from *Microbispora hainanensis* strain CSR-4; (**1**a) planar structure of compound **1** and (**1**b) 3D structure of compound **1** with the selected NOESY correlations.

Compounds **1**–**10** (Fig. 2), including one new and nine known compounds, were isolated from the EtOAc crude extract of the soil actinomycete *Microbispora* sp. CSR-4. The new diterpene compound **1**, 2α-hydroxy-8(14),15-pimaradien-17,18-dioic acid, was elucidated by using spectroscopic techniques including NMR, Mass, UV, and IR spectroscopy. Compounds **2**–**10** were identical to those identified as 1-hydroxyphenazine, 1,6-dihydroxylphenazine, phenylacetic acid, *p*-hydroxyl phenyl acetic acid, indole-3-carboxylic acid, indole-3-acetic acid, methyl indole-3-acetate, anthranilic acid, and 3-pyridinecarboxylic acid, respectively^46–53^.

Compound **1** was obtained as a brown oil and the HRESIMS (Additional files: Fig. S6) established a molecular formula C_20_H_28_O_5_, deducing from the sodium-adduct mass ion at *m/z* 371.1826 [M+Na]^+^. The IR spectrum (Additional files: Fig. S7) showed the characteristic absorption bands of carboxylic hydroxyl (at ν 2,800–3,600 cm^−1^) and carboxylic carbonyl (at ν_max_ 1698 cm^−1^) functionalities. The ^13^C NMR and DEPT spectra (Table 1) of compound **1** displayed signals of two methyls, seven methylenes, five methines (two sp^2^ methines and three sp^3^ methines), and six quaternary carbons. The ^1^H and ^13^C NMR spectral data (Additional files: Fig. S8, S9) were similar to those of metaglyptin M (2α-hydroxy-pimara-8(14), 15-dien-18-oic acid)^54^, except a lack of a methyl group at C-13 and an additional quaternary carbon at δ_C_ 178.9 (C-17). The NMR data suggested a replacement of methyl with a carboxylic acid. In addition, the HMBC spectrum confirmed the position of an extra carboxylic at C-13 by showing correlation from two methines at H-14 (δ_H_ 5.66) and H-15 (δ_H_ 5.97) to C-17 (δ_C_ 178.9). The multiplicity of H-2 signal as doublet of doublet of triplets indicated its position at axial (Fig. 2, **1**b). Moreover, the NOESY spectrum showed cross-peak correlations (Fig. 2, **1**b) from H_3_-19 to H_3_-20 and H-2 (δ_H_ 3.80); and from H-14 to H_β_-7 (δ_H_ 2.41) and H_a_-16 (δ_H_ 5.11), confirming a hydroxy at C-2 was at α-position and COOH at C-13 was at β-position. Thus compound **1** (Fig. 2, **1**a) was 2α-hydroxy-8(14), 15-pimaradien-17,18-dioic acid.

**Table 1:** The ^1^H and ^13^C assignments of 2α-hydroxy-8(14),15-pimaradien-17,18-dioic acid (compound 1).

Position	Compound **1** in CD_3_OD	
	δ_H_ (mult., *J* in Hz)	δ_C_, type
1	1.08 (t, 11.9)/1.97 (dd, 11.9, 2.1)	48.3, CH_2_
2	3.80 (ddt, 11.9, 11.8, 3.9)	69.0, CH
3	1.70 (dd, 11.8, 11.8)/1.84–1.89 (m)	46.4, CH_2_
4	–	49.3, qC
5	1.95 (dd, 12.5, 2.0)	49.8, CH
6	1.32–13.9 (m)/1.49 (ddd, 12.9, 12.5, 4.5)	25.1, CH_2_
7	2.17 (ddd, 12.7, 12.7, 5.4)	36.4, CH_2_
8	–	141.07, qC
9	1.89–1.92 (m)	52.7, CH
10	–	40.4, qC
11	1.43 (dt, 10.5, 2.6)/1.58–1.66 (m)	19.2, CH_2_
12	1.72 (dd, 12.0, 12.0)/1.92 (ddd, 12.0, 7.9, 1.9)	32.0, CH_2_
13	–	51.7, qC
14	5.66 (s)	123.3, CH
15	5.97 (dd, 17.4, 10.3)	142.5, CH
16	5.11 (d, 17.4, 0.9)/5.20 (dd, 10.3, 0.9)	117.4, CH_2_
17	–	178.9, qC
18	–	181.5, qC
19	1.21 (s)	18.5, CH_3_
20	0.83 (s)	16.5, CH_3_

*2α-hydroxy-8(14),15-pimaradien-17,18-dioic acid *(***1***), brown oil; [α]_D_^25^ + 25.65 (*c* 0.16, MeOH); UV λ_max_, nm (log ε, MeOH) 233 (3.26), 267 (3.21); IR (ATR) *ν*_max_, cm^−1^ 3,600–2,800 (br), 2,925, 2,854, 1,698, 1,468, 1,391, 1,371, 1,252, 1,233, 1,150, 1,129, 1,030, 1,008, 964 and 923 (Fig. S7); HRESIMS *m/z* [M+Na]^+^: 371.1826 (calcd for C_20_H_28_O_5_Na, 371.1829) (Fig. S6).

Compounds **2** was obtained as a brown solid and its molecular formula C_12_H_8_N_2_O as deduced by mass ion peak at *m/z* 197.0715 [M+H]^+^ in the HRESIMS spectrum (Additional files: Fig. S10). The ^1^H and ^13^C spectra data (Additional files: Fig. S11, S12) suggested to be 1-hydroxyphenazine, whose NMR information described in the literature^55,56^ was compared with our data to confirm its identity.

Compound **3** was obtained as a dark yellow solid and HRESIMS data (Additional files: Fig. S13) indicated 16 mass units higher than that of compound **2** by revealing the mass ion peak at *m/z* 213.0657 [M+H]^+^, which corresponded to the molecular formula of C_12_H_8_N_2_O_2._ This compound was identified as 1,6-dihydroxyphenazine by comparing their ^1^H and ^13^C NMR data (Additional files: Fig. S14, S15) with the previous reports^57,58^. Compounds **2** and **3** were generally isolated from soil and marine microorganisms e.g. *Streptomyces* spp.^47^ and *Pseudomonas aeruginosa*^59^.

Compounds **4** was obtained as a brown gum. HRESIMS spectrum (Additional files: Fig. S16) revealed the molecular formula C_8_H_8_O_2_ by giving a sodium-adduct mass ion at *m/z* 159.0437 [M+Na]^+^. Comparison of the ^1^H NMR and ^13^C NMR spectra (Additional files: Fig. S17, S18) with the published data^48^ proved the identity of compound **4** to be phenylacetic acid.

Compound **5** was obtained as a brown gum and the HRESIMS (Additional files: Fig. S19) confirmed the molecular formula C_8_H_8_O_3_ by showing a sodium-adduct ion peak at *m/z* 175.0373 [M+Na]^+^. The ^1^H NMR and ^13^C NMR spectra (Additional files: Fig. S20, S21) were similar to those reported in the literature^51^ as *p*-hydroxylphenylacetic acid. Both compounds **4** and **5** were typically isolated from plants and microorganisms with interesting biological activities. Several genera of the actinomycetes and filamentous fungi e.g. *Micromonospora*^48^, *Streptomyces*^49^, and *Aspergillus*^50^ have been reported as the producer of these compounds.

Compound **6** was obtained as a brown solid. The molecular formula of compound **6** suggested to be C_9_H_7_NO_2_ from the HRESIMS (Additional files: Fig. S22), which displayed the sodium-adduct mass ion peak at *m/z* 184.0391 [M+Na]^+^. Comparing the NMR data with references^52^, compound **6** was indole-3-carboxylic acid (Additional files: Fig. S23, S24).

Compounds **7** and **8** were obtained as a brown solid and brown gum. HRESIMS data (Additional files: Fig. S25, S28) determined the molecular formula to be C_10_H_9_NO_2_ and C_11_H_11_NO_2_, by giving the sodium-adduct mass ion peaks at *m/z* 198.0524 [M+Na]^+^ and 212.0684 [M+Na]^+^, respectively. Their ^1^H and ^13^C NMR data (Additional files: Fig. S26, S27, S29, S30) were compared with previously reported data^53^, which confirmed that compounds **7** and **8** were indole-3-acetic acid and methyl indole-3-acetate, respectively. Indoles are commonly found in both plants and actinomycetes. Indole derivatives have been isolated from various genera of soil actinomycetes such as *Streptomyces*^60^, *Micromonospora*^48^, and *Microbispora*^61^.

Compound **9** was obtained as a brown solid. HRESIMS spectrum (Additional files: Fig. S31) showed a deprotonated molecular ion peak at *m/z* 136.0402 [M–H]^–^_,_ revealing the molecular formula of C_7_H_7_NO_2_. The ^1^H and ^13^C NMR spectral data of compound **9** (Additional files: Fig. S32, S33) were compared with those of anthranilic acid^52,62^, which was isolated from *Streptomyces* sp. B-9–1. They are identical, thus compound **9** is anthranilic acid.

Compounds **10** was obtained as a brown solid and its molecular formula was determined to be C_6_H_5_NO_2_ by HRESIMS spectrum (Additional files: Fig. S34), showing the sodium adduct ion peak at *m/z* 146.0304 [M+Na]^+^. Together with the ^1^H and ^13^C NMR spectral data (Additional files: Fig. S35, S36), compound **10** was identified as 3-pyridine carboxylic acid, which was earlier produced from *Nocardiopsis* sp. 236^58^.

### In vitro and in silico Inhibition of AChE of the new compound

**Figure 3:**
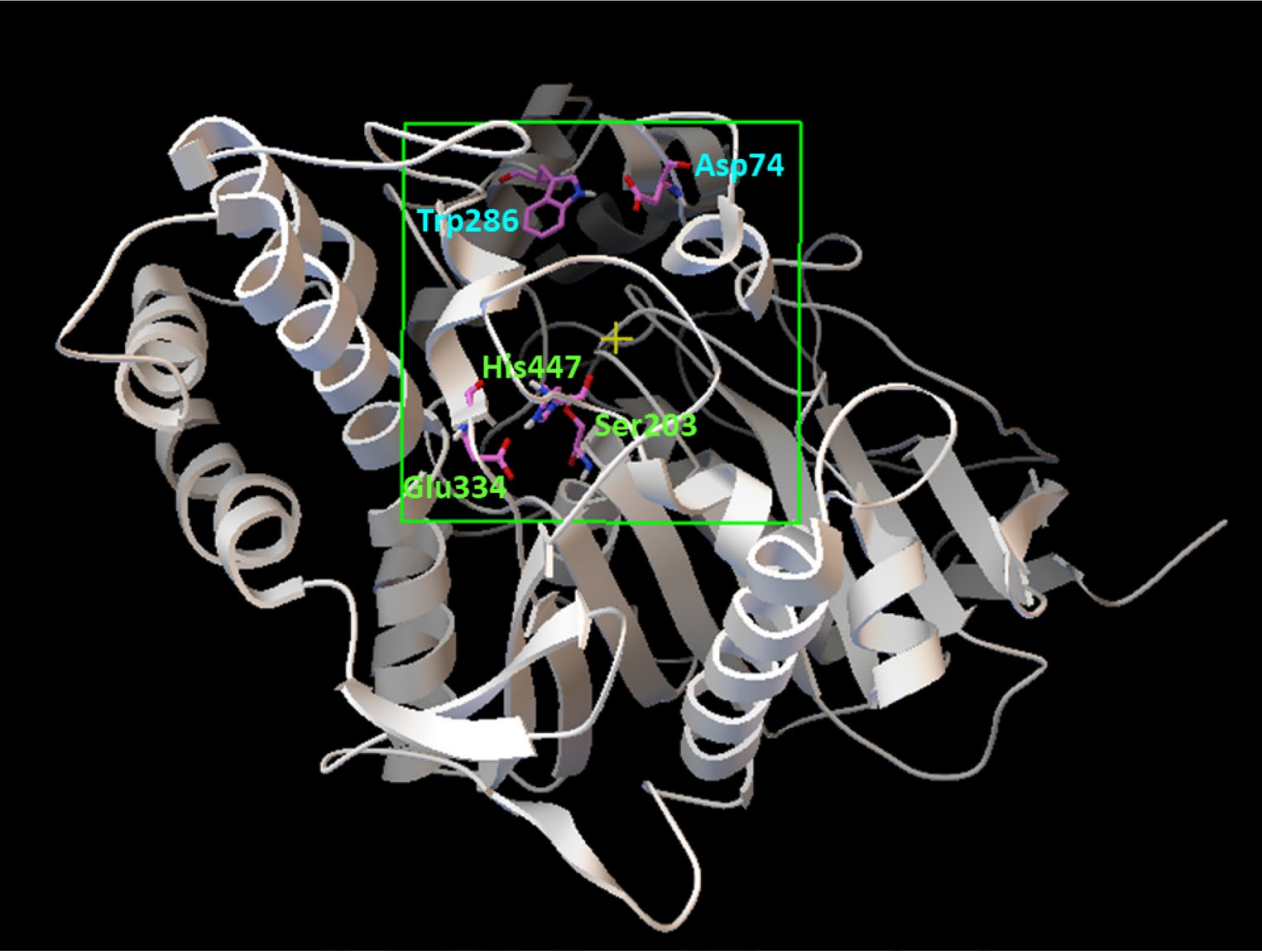
The recombinant human acetylcholinesterase; rhAChE (PDB ID: 4EY6) representing the cubical grid box with dimensions of 60 × 60 × 60 with 0.375 Å around the two active sites: the peripheral site (Asp74 and Trp286) and the catalytic site (Ser203, Glu334 and His447).

**Figure 4:**
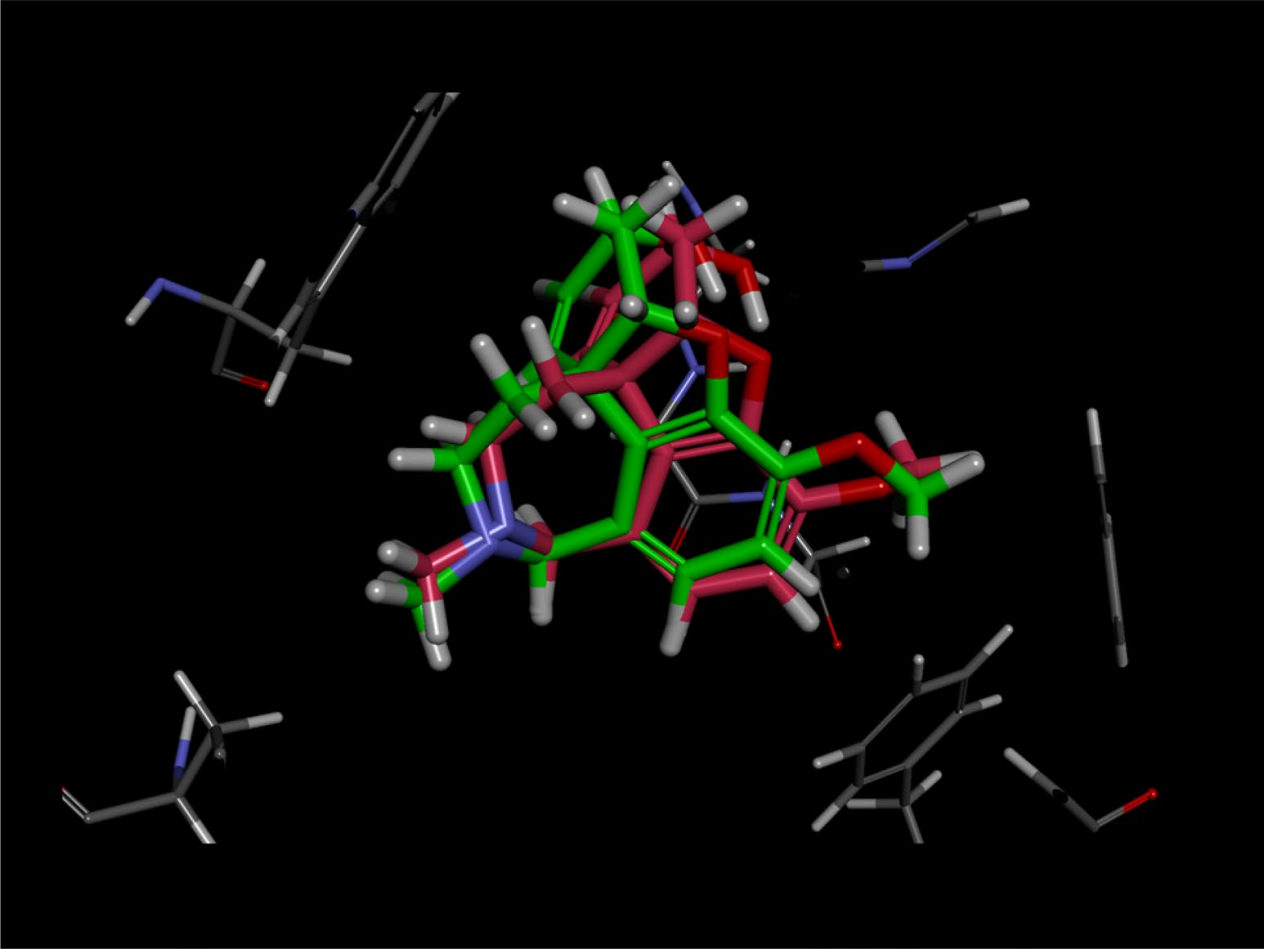
Re-docked pose of galantamine into the binding site of rhAChE. The structures of galantamine were represented in stick by superposition of docked pose (pink) and co-crystallized structure (green).

**Figure 5:**
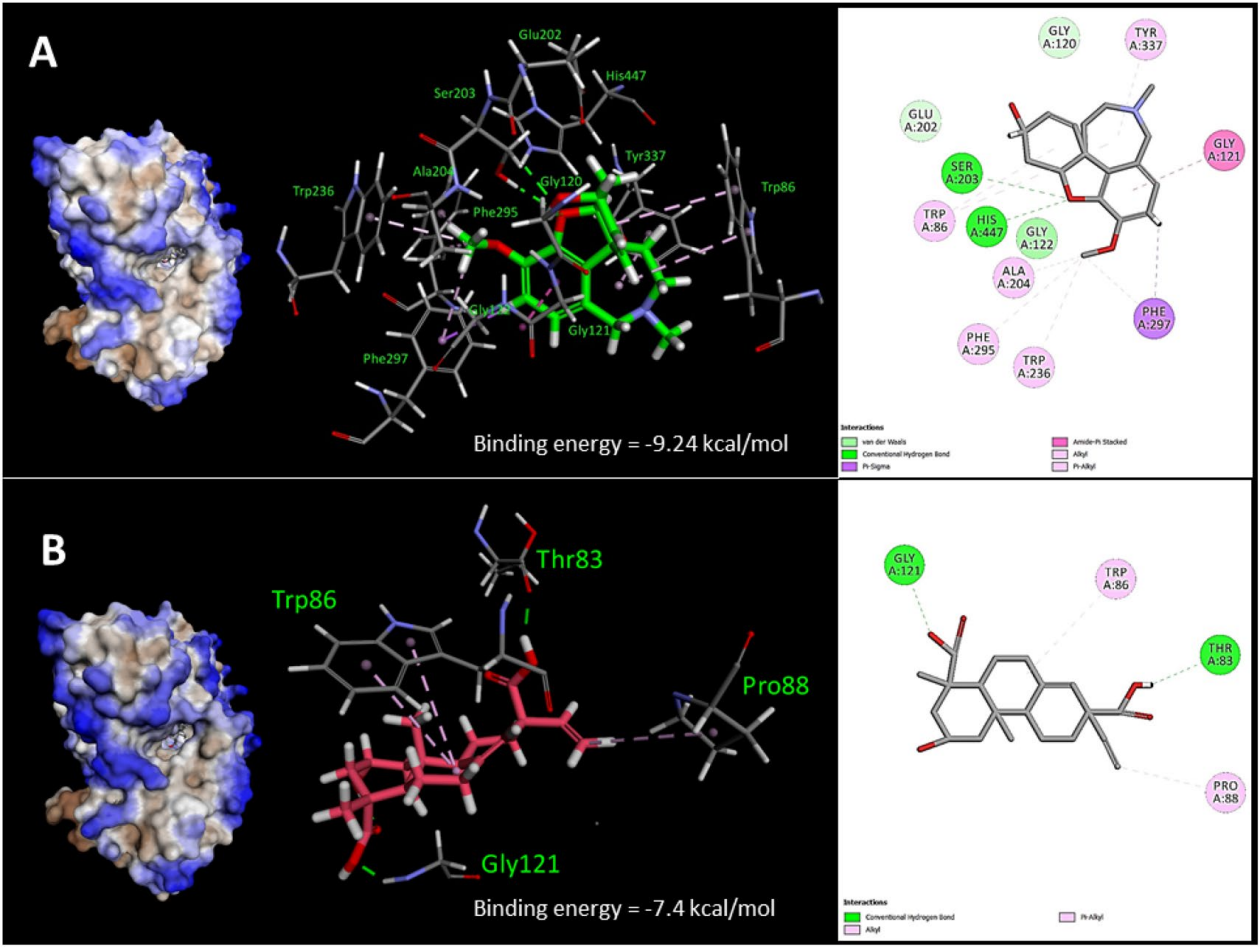
The docked galantamine (**A**) and compound **1** (**B**) in the binding site of rhAChE representing for binding energy (kcal/mol) and bonding types of hydrophobic interaction and hydrogen bond. The graphical representations for binding interactions were illustrated in 3D and 2D diagram.

The crude extract from the whole culture (broth and cells) of *Microbispora* sp. CSR-4 exhibited anti-AChE activity by showing 35.36 ± 2.34% to 58.25 ± 1.47% depending on the concentration. Thus, further study on the chemical ingredient with anti-AChE activity was then conducted. The investigation led to the isolation of the new diterpene 2α-hydroxy-8(14),15-pimaradien-17,18-dioic acid (compound **1**). Compound **1** (at 100 µg/ml) possessed 52.81 ± 1.24% inhibition of the anti-rhAChE activity, whereas galanthamine (positive control) exhibited was 88.81 ± 1.32% inhibition at the same concentration (Additional files: Table S6). To analyze the binding mode of compound **1** in the active sites of rhAChE, the molecular docking technique by Autodock was performed. The reliability of the docking protocol was proven by re-docking of (–)-galantamine into the binding site of rhAChE. The cubical grid box was generated by covering the two active sites: the peripheral site (Asp74 and Trp286) and the catalytic site (Ser203, Glu334, and His447) as represented in Fig. 3. The docking scores were calculated by the default scoring function. The best-docked pose of galantamine presented the lowest negative score of the binding energy of − 9.24 kcal/mol. The docked conformation with respect to the crystal conformation of galantamine in rhAChE (PDB ID: 4EY6) showed the RMSD value of 0.7731 Å (Fig. 4) indicating a significantly reliable protocol since the threshold of reliability was 2.0 Å for a good docking^63^. The compound **1** was then docked into the active site pocket of rhAChE using the same protocol as a galantamine docking condition. The results of docking analyses showed that all binding energies of 100 docked poses of compound **1** were in the range of − 5.92 to − 7.54 kcal/mol. The best pose of compound **1** in the binding site of rhAChE was selected by pose clustering analysis. The protocol was employed with a cutoff value of 2 Å. The lowest energy docked model (the most negative value) of the most populated cluster was chosen to indicate the suitable docking pose. The best docking pose of compound **1** showed the highest energy score of − 7.54 kcal/mol among 76 poses of the largest cluster. The binding affinity and nonbonding interactions of the best-docked complexes obtained from molecular docking analysis were illustrated in Fig. 5. According to the pose docking analysis, the binding affinity demonstrated that the binding energy of galantamine (− 9.24 kcal/mol) was more stable than compound **1** (− 7.54 kcal/mol). It was revealed that galantamine showed π-alkyl interaction with several residues including Tyr337 of the PAS in the active site of rhAChE. Moreover, two hydrogen bonds were found on Ser203 and His447 that are the residues of catalytic triad in the CAS of rhAChE. Whereas the binding mode of compound **1** showed π-alkyl interaction with Trp86 residue of the anionic site of rhAChE and two hydrogen bonds with Thr83 and Gly121 of oxyanion hole (Fig. 5). This was the reason why the AChE inhibitory activity of galantamine was more active than compound **1** since the binding mode of compound **1** in the active site was less stable.

### Molecular dynamics analysis for prediction of the binding mode

**Figure 6:**
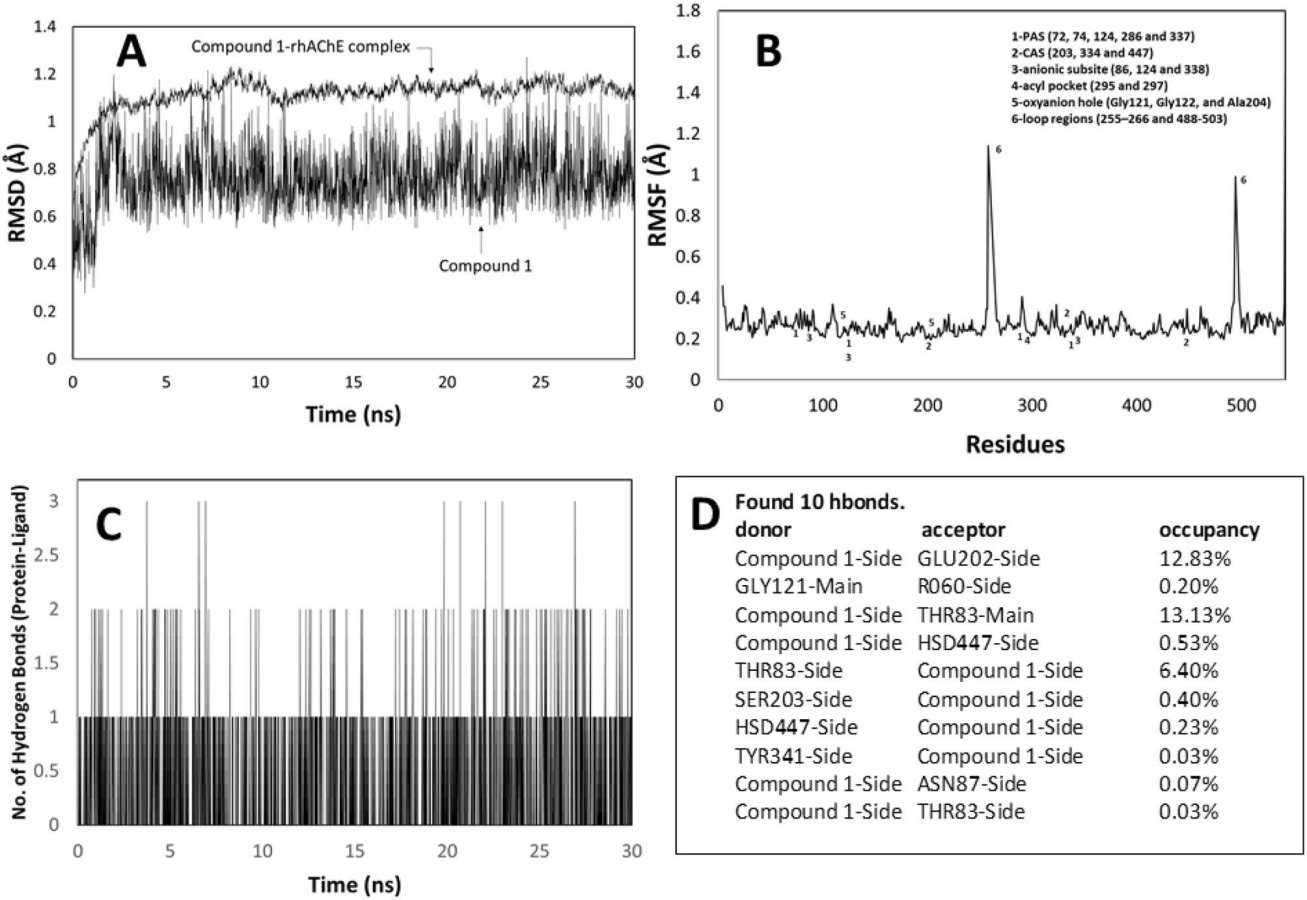
The trajectory analyses along 30 ns of MD simulations. The root mean square deviation (RMSD) values of all atoms in the compound 1-rhAChE complex and heavy atoms in the compound **1** were plotted (**A**). The root means square fluctuations (RMSF) of Cα atoms of rhAChE in the complex (**B**), the total number of hydrogen bonds formed between the rhAChE and compound **1** in the complex state during simulation (**C**) and its analysis (**D**) were also illustrated.

**Figure 7:**
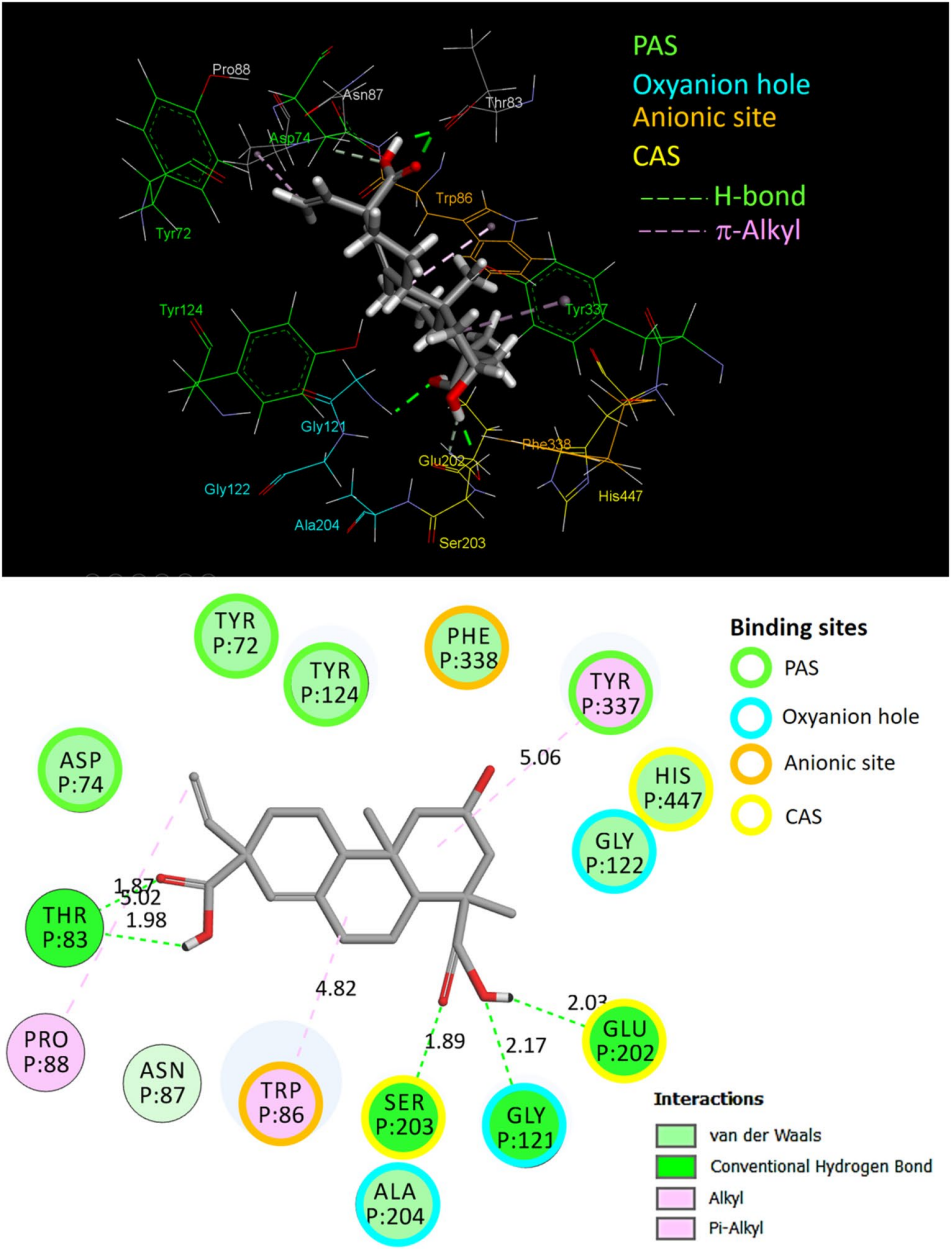
The optimized binding mode of compound **1** in the binding site of rhAChE representing for the energy minimized conformation of the last step in the 30 ns long MD simulation. The binding residues within a radius of 4 Å from the bound compound **1** were illustrated in 3D graphic (**A**) and 2D diagram (**B**), indicating the types of binding interactions and interacting amino acids in the peripheral anions site (PAS), anionic sub-site, oxyanion site and catalytic active site (CAS) of the rhAChE.

To better understand the binding mode of compound **1**, the rigid structure of the compound **1**-rhAChE complex that was obtained from the docking result was further optimized using MD simulations. The MD simulation of the compound 1-rhAChE complex was carried out up to 30 ns. The root mean square deviation (RMSD), the root mean square fluctuation (RMSF), and the number of hydrogen bonds formed between compound **1** and rhAChE were calculated for compound **1**-rhAChE complex and plotted in a time-dependent manner (Fig. 6). All atoms RMSD of the compound **1**-rhAChE complex was observed to archive equilibrium after 5 ns and fluctuated around 1.12 Å indicating that the structure of the complex was stable (Fig. 6A). Moreover, the RMSD of compound **1** (heavy atoms only) in the complex fluctuated around 0.77 Å (Fig. 6A). This average RMSD of compound **1** in the complex was less than 2.0 Å indicating that the binding mode of compound **1** was judged to be stable^64^. Accordingly, the RMSD trajectory plot reveals the conformational stability of the compound 1–rhAChE complex. Further, RMSF of Cα atoms were calculated based on the 30 ns MD trajectory and plotted to identify the fluctuating area of rhAChE in the complex (Fig. 6B). Typically, it was found that the amino acids in the loop regions (255–266 and 488–503) fluctuated than the other parts of the protein. However, such a trend was not found in other regions including the peripheral anions site (PAS), anionic subsite, oxyanion site, and catalytic active site (CAS) as the fluctuation was relatively small. This result indicated that the conformation of the entire binding sites of rhAChE facing compound **1** was quite fixed and likely to contribute to the stability of ligand binding. To demonstrate the stability of this complex, intermolecular hydrogen bonds interacting between compound **1** and rhAChE were determined and represented in Fig. 6C. The result showed 1–3 hydrogen bond contacts along 30 ns of simulations. A total of ten hydrogen bonds were found between compound **1** and rhAChE as an acceptor or donor residues (Fig. 6D). However, the high occupancy of hydrogen bond occurred at Glu202 (12.83%) and Thr83 (13.13%), of which the latter also found in the docking pose of compound **1**. Finally, the complex structure at the end of 30 ns of MD simulation was submitted for energy minimization prior to the analysis of binding interactions within 4 Å around compound **1** (Fig. 7). The results showed that compound **1** bound with amino acid residues in PAS (Tyr72, Asp74, Tyr124, and Tyr337), oxyanion hole (Gly121, Gly122, and Ala204), anionic site (Trp86 and Phe338) and CAS (Glu202, Ser203, and His447) of the active site of rhAChE. Four hydrogen bonds formed between compound **1** and amino acid residues in CAS (Glu202 and Ser 203), oxyanion hole (Gly121), and side door region (Thr83) of rhAChE^65^ that stabilized the complex. The hydrophobic interactions that supported the binding stability of compound **1** were represented by alkyl interaction with Pro88 and π-alkyl interaction with Trp86 and Tyr337. In the light of MD simulation and optimized structure of the compound **1**-rhAChE complex, all of the results indicated that compound **1** could be a potential active human AChE inhibitor.

### Neuroprotective, antioxidant and cytotoxic activities of the new compound

**Figure 8:**
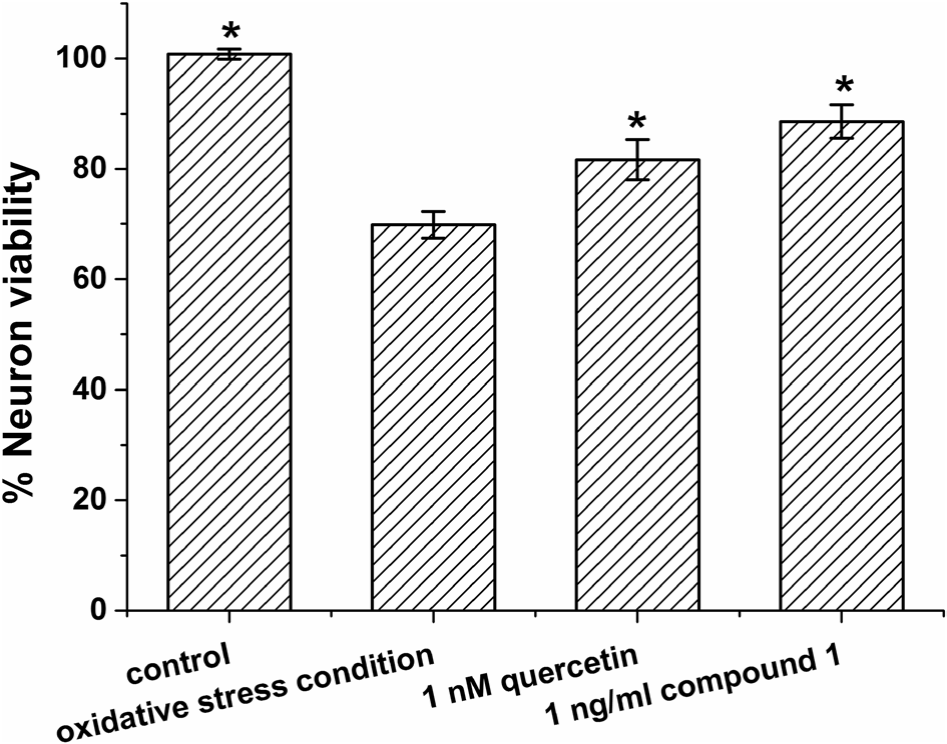
Neuroprotective ability at 1 ng/ml of compound 1 on P19-derived neuron. The error bar represented standard error of the mean (SE). (*p < 0.05 when compared to oxidative stress condition produced by serum deprivation).

The crude extract from the whole culture (broth and cells) of *Microbispora* sp. CSR-4 exhibited an ability for neuronal protection by showing 122.19 ± 2.29% viability of neurons at the concentration of 1 ng/ml, suggesting a presence of a compound with the ability to prevent neuronal cell death led us to focus on the isolation of its active component. In this study, we found that our new compound 1 (at 1 ng/ml) enhanced the viability of the P19-derived neuron showing the % neuron viability (113.91 ± 9.41%) more than that of the control (99.87 ± 0.13%) (Additional files: Fig. S37). Then, further evaluation of the neuroprotective ability was performed at this concentration (1 ng/ml of compound **1**). The result showed that compound **1** had a significant neuroprotective activity at 1 ng/ml with % neuron viability of 88.56 ± 5.19%, compared to the oxidative stress (induced by deprivation of the serum) control group (% neuron viability = 69.86 ± 4.19%), while the positive control (quercetin) at 1 nM exhibited % neuron viability at 81.64 ± 6.28% and the control (0.5% DMSO as a solvent in complete medium) showed % neuron viability at 100.79 ± 1.65% as shown in Fig. 8. No significance observed between the positive control and the tested compound. The anti-oxidant activity was done for preliminary studying the mechanism of action on the neuroprotective activity of the compound. The result showed that at 100 µg/ml of compound **1** possessed only 9.72 ± 2.09% inhibition of the DPPH radical performed by radical scavenging assay (Additional files: Table S6). These suggested that the mechanism of action for the neuroprotective ability of compound **1** was not the responsibility for radical scavenging ability due to no anti-oxidant activity was detected at the same concentration of neuroprotective activity. Additionally, compound **1** was found to be inactive to normal cells (Vero cell) at 1,000 µg/ml (Additional files: Table S6). Moreover, compounds **2** and **3** showed antifungal activity against *Candida albicans* (MIC 31.25 µg/ml and > 250 µg/ml)^55,57^ and compound **9** acted as a spore germination inhibitor (IC_50_ at ca. 50 µg/ml) of *Streptomyces* spp., isolated from root tumor of melon^62^. To our knowledge, the compound, 2α-Hydroxy-8(14),15-pimaradien-17,18-dioic acid, was a new active diterpene compound obtained from the EtOAc crude extract of *Microbispora* sp. CSR-4 and possessed neuroprotective and anti-acetylcholinesterase activities without cytotoxicity. Therefore, this compound is a potential molecule for development as anti-Alzheimer’s agent.

### Preliminary in silico pharmacokinetics

The preliminary pharmacokinetics related properties of compound **1** were predicted using the SwissADME web-based application^66^. The predicted physicochemical properties and ADME pharmacokinetics related properties were included (Additional files: Fig. S38). Based on the Lipinski`s Rule for the central nervous system drugs (RoCNS), compound **1** revealed CNS drug-likeness properties i.e., molecular weight = 348.43 (MW < 400), Consensus LogP = 2.57 (CLogP ≤ 5), a number of H-bond acceptor = 5 (HBA ≤ 7) and a number of H-bond donor = 3 (HBD ≤ 5)^67,68^. Our findings showed that these properties are consistent with the molecular property of CNS drugs that have significantly lower numbers of H-bond donors and H-bond acceptors compared with that of non-CNS drugs^69,70^. The topological polar surface area (TPSA) of compound **1** was 94.83 Å^2^ that was a little higher than 90 Å^2^ as a cutoff for optimal CNS exposure^71^. Compound **1** contains the number of rotatable bonds of 3 that was well agreed with the proposed guideline of a rotatable bond count < 8 as an attribute of a successful CNS drug candidate^72^. However, the pharmacokinetics BBB permeation of compound **1** that was predicted by the Brain Or IntestinaL EstimateD permeation method (BOILED-Egg model)^73^ showed that compound **1** might not be able to pass through the blood–brain barrier and penetrate into the CNS (Additional files: Fig. S38). The plotting between the lipophilicity (WLOGP) and polarity (tPSA) of compound **1** showed that it was outside the BBB permeation area of the BOILED-Egg model. Although the lipophilic of compound **1** (WLOGP 3.24) was in the range of relatively lipophilic (log P from + 0.4 to + 6.0) of the model, the tPSA (94.83 Å^2^) of compound **1** was higher than the cutoff value of the moderately polar PSA (79 Å^2^) of the model. This was the reason why compound **1** was predicted as a brain non-penetrant molecule. Accordingly, the structure of compound **1** may be further optimized and/or experimentally measured the BBB permeability to develop compound **1** as a new lead for CNS active anticholinesterase inhibitor in the field of Alzheimer’s disease pharmacotherapy.

## Conclusions

In conclusion, we herein reported the identification and genome properties of *Microbispora* sp. CSR-4. The taxonomic characterization revealed that strain CSR-4 was identified as *Microbispora hainanensis*. Analysis of the draft genomes of strain CSR-4 revealed the presence of abundant smBGCs e.g. terpene, PKS, LAP, NRPS-like fragment, and bacteriocin gene clusters are of particular interest in the search for novel antibiotics. This suggested that strain CSR-4 was a potential source of the production of bioactive metabolites. The crude extract showed anti-AChE and neuroprotective activity and the chemical investigation led to the isolation of nine known compounds and one new diterpenoid compound. This is the first report for the discovery of a new diterpenoid compound produced by *Microbispora*. The diterpenoid compound also exhibited in vitro anti-AChE activity. We used molecular docking and MD simulations to understand its ability as the inhibitor against rhAChE activity. The prediction of the diterpenoid compound-rhAChE complex was obtained from the top rank pose with the highest score (in minus) of binding energy using molecular docking. The binding mode of the diterpenoid compound in the active site of rhAChE was analyzed from the optimized structure after molecular dynamics simulation for 30 ns and the result showed that the diterpenoid compound had interaction with peripheral anions site (PAS), anionic subsite, oxyanion site and catalytic active site (CAS) of the rhAChE. The key residues involving in the binding interactions were (1) Thr83, Glu202, Ser203, and Gly121 for hydrogen bond interactions and (2) Pro88, Trp86, and Tyr337 for hydrophobic interactions that promoted the stability of binding mode in the diterpenoid compound. Furthermore, the new diterpenoid compound showed the neuroprotective activity and no cytotoxicity to Vero cells. For preliminary in silico pharmacokinetics, compound **1** was predicted as a potential CNS drug candidate based on Lipinski`s Rule for central nervous system drugs (RoCNS). However, it was predicted as a non-blood–brain barrier (BBB) penetrant molecule. Therefore, further study is needed to develop this compound as a new lead of CNS active anti-cholinesterase inhibitor for the treatment of Alzheimer’s disease.

## Methods

### Isolation, cultivation, and preservation of strain CSR-4

An actinomycete strain CSR-4 was isolated rhizosphere soil of *Zingiber cassumunar*. The isolation process was performed according to the protocol of Kittisrisopit et al.^30^. Briefly, the air-dried soil was heated at 120 °C for 1 h and kept continuously in a desiccator containing silica gel beads for 7 days. The diluted 1,000-fold soil solution was prepared by serial dilution technique with 0.01% sterile SDS in distilled water and spread onto soil extract agar (1 g soluble starch, 0.1 g KNO_3_, 0.005 g FeSO_4_⋅7H_2_O, 0.005 g MgSO_4_⋅7H_2_O, 0.001 g CaCl_2_⋅2H_2_O, 1.5 g agar, 100 ml soil extract solution; pH 7.2) supplemented with 20 mg nalidixic acid l^−1^ and 50 mg nystatin. After incubation at 30 °C for 21 days, a pale yellowish-brown colony of strain CSR-4 was isolated and purified on yeast extract-malt extract agar (International *Streptomyces* Project, ISP2 medium)^74^. The pure culture was maintained in glycerol solution (20%, v/v) at – 80 °C or lyophilized for long-term preservation.

### Taxonomic characterization

#### Morphological, cultural and physiological characteristics

Morphology properties of strain CSR-4 were first observed by light microscopy (ECLIPSE E200; Nikon) using cultures grown on ISP 2 agar at 30 °C for 14 days. Then, the arrangement of spores was observed by scanning electron microscopy (JSM-6610 LV; JEOL). Cultural characteristics were determined after 14 days at 30 °C using International *Streptomyces* Project (ISP) media 1–7. The ISCC-NBS color charts^75^ were used for assigning the colors of the aerial and substrate mycelia and any soluble pigments. Growth at various conditions i.e. temperature (10–60 °C), NaCl tolerance (0–7% w/v) and pH (4–12 at intervals of 1 pH units) were tested in ISP2 broth for 14 days. The utilization of sole nitrogen sources, decomposition of adenine, hypoxanthine, xanthine, tyrosine and cellulose, hydrolysis of starch, reduction of nitrate, peptonization, and coagulation of milk, liquefaction of gelatin and acid production from carbon sources were examined as described previously^76–78^. Enzyme productions (API ZYM assays) were carried out using ISP2 agar as the basal medium. The reference strain, *Microbispora hainanensis* DSM 45428^T^, was cultured under the same conditions for comparative analyses.

#### Chemotaxonomic analyses

Dried cells of strain CSR-4 and *M. hainanensis* DSM 45428^T^, the reference strain, were prepared by cultivation in ISP2 broth on a rotary shaker (200 rpm) at 30 °C for 5 days and cells were harvested by centrifugation. The cell pellets were then washed with sterile distilled water 3 times and freeze-dried. To determine the isomer of diaminopimelic acid (DAP), reducing sugars in cell hydrolysates and type of menaquinones, the standard methods of Hasegawa et al.^79^, Komagata and Suzuki^80^ and Collins et al.^81^ were used in this study, respectively. Polar lipids were extracted and determined according to the method described by Minnikin et al.^82^. Cellular fatty acid profile analysis was carried out using GC (model 6890; Agilent) according to the instructions of the Microbial Identification System (MIDI) Sherlock version 6.0^83,84^ with the Microbial Identification software package based on Sherlock Aerobic Bacterial Database (TSBA6).

#### Genotypic analyses

Genomic DNA for PCR amplification was extracted according to a previously described method^85^. Amplification of the nearly complete 16S rRNA gene was performed using the universal primers 9F (5′-GAGTTTGATCCTGGCTCAG-3′) and 1541R (5′-GTTACCTTGTTACGACTT-3′)^86^. The experimental condition was as follows: initial denaturation at 94 °C for 3 min; 40 cycles of 94 °C for 30 s, 56 °C for 30 s and 72 °C for 90 s; and a final extension at 72 °C for 5 min. Sequencing of the PCR product was carried out using the 780R (5′-CTACCAGGGTATCTAATCC-3′), 350F (5′-TACGGGAGGCAGCAG-3′), 780F (5′-GATTAGATACCCTGGTAG-3′) and 1541R primers^87^. For calculating and comparing levels of similarity, the 16S rRNA gene sequence of strain CSR-4 was submitted to the EzBioCloud server^33^. CLUSTAL W multiple alignment modes within the BioEdit program version 7.1.3.0^88^ was used to align nearly full-length 16S rRNA gene sequences obtained in this study with the type strains of *Microbispora* species retrieved from EzBioCloud database. The neighbor joining^31^ and maximum-likelihood^32^ trees were reconstructed with MEGA version 6.0 program^89^ and all positions containing gaps and missing data were eliminated using complete deletion option in the same program. A distance matrix was generated using Kimura’s two-parameter model^90^. The stability of the clades in the trees was assessed by bootstrap analysis with 1,000 resamplings^91^. Genomic DNAs for whole-genome sequencing of strain CSR-4 and *M. hainanensis* DSM 45428^T^ were extracted from 3-day-old cultures grown in ISP 2 broth at 30 °C. The GeneJET Genomic DNA purification Kit (Thermo Scientific) was used for purification. Whole-genome shotgun (WGS) sequencing was performed using an Illumina MiSeq 1 TB platform (Illumina, Inc., San Diego, US-CA) and assembled de novo by using SPAdes version 3.10.1^92^. The annotation was performed by using the Prokka software 1.12^93^ in line with the NCBI Prokaryotic Genome Annotation Pipeline (PGAP). To calculate the average nucleotide identity (ANI) and the digital DNA G + C values, ANI-MUMmer (ANIm) algorithms^94^ within the JSpeciesWS webservice^35^, the online tools from Environmental Microbial Genomics Laboratory (Kostas lab)^34^ and EzBioCloud server (https://www.ezbiocloud.net/tools/ani)^95^ were used. A phylogenomic tree of strain CSR-4 and their closest type strains was constructed by using TYGS web server (https://tygs.dsmz.de/)^96^. The digital DNA–DNA hybridization value between the genome of strain CSR-4 and the most closely related species was determined using the Genome-to-Genome Distance Calculator, version 2.1^37^. The secondary metabolite biosynthesis gene clusters in the bacterial genome were analyzed using anti-SMASH program^40^. For genome visualization, CGView application^97^, a highly customizable circular genome rendering system, was implemented. The resulting circular map shows annotated genes and COG category classifications. The synteny map between strain CSR-4 and *M. hainanensis* DSM 45428^ T^ was built using Artermis Comparison Tool (ACT)^39^.

### The accession number of *Microbispora* sp. CSR-4 and the reference strain, *M. hainanensis* DSM 45428^T^

The GenBank/EMBL/DDBJ accession number for the complete 16S rRNA gene sequence of *Microbispora* sp. CSR-4 is LC383886 and of *M. hainanensis* DSM 45428^T^ is FJ261972. The Whole Genome Shotgun projects for *Microbispora* sp. CSR-4 and *M. hainanensis* DSM 45428^T^ have been deposited at DDBJ/ENA/GenBank under the accession VJVX00000000 and VIRM00000000, respectively. The versions described in this paper are versions VJVX01000000 and VIRM01000000, respectively. Strain CSR-4 is deposited in Thailand Bioresource Research Center for code number TBRC 10616 (Additional files: Fig. S39).

### Fermentation, extraction, and isolation of a bioactive substance

*Microbispora* sp. CSR-4 was cultivated on ISP2 agar at 30 °C for 5 days. A loopful of the strain from an agar culture was inoculated into a 250 ml Erlenmeyer flask (20 flasks), containing 100 ml ISP2 medium (composed of (% w/v): 0.4% glucose, 0.4% powdered yeast extract, and 1.0% powdered malt extract in distilled water at pH 7.2). The culture was incubated for 4 days at 30 °C on a rotary shaker at 180 rpm. Then an equal volume (2 ml) of the seed culture was transferred into 80 × 1 l Erlenmeyer flasks, which each contained 250 ml of ISP2 medium supplemented with 0.05% calcium carbonate at pH 7.2 and the production culture was cultured for 14 days at 30 °C on rotary shakers at 200 rpm. After the cultivation period, the mycelium was separated from the broth by centrifugation and the broth was extracted three times with equal volume of ethyl acetate (EtOAc). EtOAc was evaporated at reduced pressure to dryness to obtain a brown gum (1.69 g). The gum was precipitated with 20% dichloromethane (CH_2_Cl_2_) in methanol (MeOH) and the solid was filtered through a Whatman No. 1 membrane. The filtrate was then applied to a column of Sephadex™ LH-20 (GE healthcare Bio-Sciences AB, Sweden), eluted with 5% CH_2_Cl_2_ in MeOH as a mobile phase to give seven fractions. The active fraction (F4, 0.50 g) was re-chromatographed on a Sephadex LH-20 column (4.2 cm × 36.5 cm) eluted with the same solvent system to give six subfractions (F4f1–F4f6). The active fraction (F4f3, 0.25 g) was then purified by a preparative HPLC (performed on a Dionex–Ultimate 3000 series equipped with a binary pump, an autosampler, and a diode array detector), using a Sunfire C18 OBD column (particle size 10 µm, diam. 19 mm × 250 mm) and eluted with a linear gradient system of 5–35% CH_3_CN in a presence of 0.05% formic acid over 45 min at the flow rate of 15 ml/min, to furnish a diterpenoid compound **1**, named 2α-hydroxy-8(14), 15-pimaradien-17, 18-dioic acid (15.9 mg), compounds **4** (17.5 mg), **5** (6.8 mg), and **10** (6.5 mg). The active fraction (F4f4, 0.13 g) was further purified by a preparative HPLC, using the same column as the previous fraction and eluted with a linear gradient system of 5–35% CH_3_CN over 50 min at the flow rate of 12 ml/min, to furnish compounds **2** (1.3 mg), **3** (9.2 mg), **6** (2.3 mg), **7** (17.1 mg), **8** (2.5 mg), and **9** (21.1 mg).

### Structure identification of the active compound

UV spectrum was performed in MeOH on a Spekol 1200 spectrophotometer from Analytik Jena. Optical rotation was measured with a JASCO P-1030 digital polarimeter. FT-IR spectrum was measured on a Bruker ALPHA spectrometer. NMR spectra were acquired in acetone-*d*_*6*_ or CD_3_OD or DMSO-*d*_*6*_ on either a Bruker Avance 400 MHz or Bruker Avance 500 MHz NMR spectrometer. HRESIMS data was determined on a Bruker MicrOTOF mass spectrometer.

### Determination of anti-acetylcholinesterase (anti-AChE) activity

In vitro anti-AChE activity was conducted as previously described^98,99^, with some modifications. Briefly, the compound was dissolved in the buffer containing 50% MeOH (two-fold dilution, 0.78–1,000 µg/ml in concentrations). In a 96-well microtiter plate, 25 µl of the tested compound was added to 200 μl reaction medium [50 µl of 0.1% BSA in 50 mM Tris HCl buffer, pH 8.0, 125 µl of 3 mM 5,5′-dithiobis-(2-nitrobenzoic acid) (DTNB) in buffer containing 0.1 M NaCl and 0.02 M MgCl_2_⋅2H_2_O, and 25 µl of 15 mM *S*-acetyl thiocholine iodide (ATCI) in distilled water]. These contents were mixed and preincubated for 5 min at 37 °C. The plate was pre-read at 405 nm using a microplate reader (FLUOstar Omega, Germany). Thereafter, the reaction was initiated by the addition of 25 µl of recombinant human acetylcholinesterase (rhAChE) (0.22 U/ml). After 20 min incubation at 37 °C, the absorbance of the yellow 5-thio-2-nitrobenzoate anion produced was measured at a wavelength of 405 nm within 4–7 min. Galanthamine (0.1 mg/ml in 50% MeOH) served as the positive control. Each assay was done in triplicate.

### Structure preparations and molecular docking

The 3D structure of rhAChE in complex with (–)-galantamine (PDB ID: 4EY6) with resolution 2.3983 Å was retrieved from the RCSB protein data bank. The molecular docking analysis of compound **1** with the rhAChE was carried out to predict its geometry in the active site of rhAChE. To perform molecular docking, the molecular structure of rhAChE (chain A) was retrieved from PDB ID 4EY6, and further, chain B and water molecules were removed from rhAChE. The 3D structure of compound **1** was constructed using the Sketch Modules in the BIOVIA Discovery Studio Visualizer 2020^100^. The structures of rhAChE and compound **1** were submitted for energy minimization with the steepest descent and conjugate gradient algorithms to reduce the atomic clash. The binding mode of compound **1** in rhAChE was predicted using molecular docking technique. The AutoDock Tools (ADT) of the package MGLTools 1.5.7 RC 1 was used to prepare input files of the protein target and ligand. The polar hydrogen coordinates were added and the atomic charges were assigned using the Gasteiger–Marsili atomic charges for calculation of electrostatic interactions and desolvation energies. A simplified typing of atoms, including identification of aromatic and aliphatic carbon atoms and identification of the hydrogen bonding state of heteroatoms were applied using the AutoDock 4.2 force field parameters. The center of the grid box was respectively set at − 9.9, − 43.0 and 30.852 for X, Y, Z coordinates with the cubical grid box of 60 × 60 × 60 size with 0.375 Å around the active sites of rhAChE. A receptor grid was created around the protein binding residues i.e., W86, G120, G121, G122, Y124, E202, S203, A204, W236, F295, F297, Y337, F338, Y341, and H447^101^. In the docking protocol, three torsional counts of compound **1** were detected as the rotatable bonds that may play a role in the binding of compound **1** in the binding site of rhAChE. Using a standard protocol of the popular program AutoDock, computer-aided docking of small ligands with 6 or fewer rotatable bonds, is reasonably fast and accurate^102,103^. The docking protocol was performed with the AutoDock 4.2 software^104^. The docked poses of compound **1** within active sites of rhAChE were evaluated using a negative score of binding energy (kcal/mol).

### Molecular dynamics simulation

The complex structure was optimized by molecular dynamics (MD) simulation using the NAMD software, version 2.13^105^ with the CHARMm force field^106^. For protein and molecule, the hydrogens were added to the structures using the pH of the solution of 7.4 and ionization tolerance of 1 to establish the protonation state of those molecules. The complex was solvated in the TIP3P water box of 89.4 × 93.9 × 97.8 Å^3^. The charge of the system was neutralized with the number of 75 for Na^+^ and 65 for Cl^−^ ions with the ionic strength of 0.15 M. Initially, the system was minimized by the conjugate gradient method following by equilibration of 50,000 steps (100 ps) using NPT ensemble at 310 K and 1 atm which was controlled by the Nosé-Hoover Langevin piston method^107^. The periodic boundary condition (PBC) was set to avoid truncation effects. The Particle Mesh Ewald (PME) method^108^ was used for the calculation of long-range electrostatic forces. All bonds with the hydrogen of water were constraint with SHAKE algorithm^109^. The full system of MD was run for 30 ns for the production step with the same NPT conditions as shown in equilibration. The trajectories were saved every 2 ps for analysis. The stability of a complex structure was evaluated using the root mean square deviation (RMSD) and root mean square deviation fluctuation (RMSF). The number of hydrogen bonds formed throughout a trajectory was calculated using the HBonds Plugin, Version 1.2 in the VMD Version 1.9.3^110^. The default criteria for the formation of a hydrogen bond between the donor (D) and acceptor (A) was less than the cut-off distance D-A (default 3.0 Å) and the angle D-H-A was less than the cut-off angle (default 20°). The graphics visualization tool for viewing, analyzing protein, and modeling data were carried out using the free DS Visualizer^100^.

### Preliminary in silico pharmacokinetics

The preliminary pharmacokinetic properties of compound **1** were predicted using the SwissADME web-based application^66^.

### Neuroprotective assay

The assay was performed in a 96-well plate for 3 independent experiments and each experiment was run in triplicate. First of all, the sample was evaluated for its ability to enhance the viability of the cholinergic P19-derived neuron by XTT reduction assay as described previously^111^ at various concentrations (1–10,000 ng/ml). The concentration that enhances the viability of the neuron more than the %neuron viability of the control (0.5% DMSO in the medium was used as a control representing no effect on the neuron viability) will be selected to further evaluated for neuroprotective activity by serum deprivation method. Quercetin at 1 nM concentration was used as a positive control for the neuroprotective assay.

### Statistical analysis

One-way analysis of variance test and post evaluated by %LSD from Origin Pro 9.0 was used to analyze the significant difference of average % neuron viability on the neuroprotective assay. Data were presented in the form of mean with standard deviation and considering *P*-value < 0.05 as significant.

### Antioxidant assay

The antioxidant capacity was estimated in terms of radical scavenging activity according to a modified version of Brand-Williams method^112^. Briefly, 100 μl of tested compound (1–1,000 µg ml^−1^ dissolved in MeOH) was mixed with 100 μl of freshly prepared DPPH solution (3 × 10^−5^ M dissolved in MeOH). The reaction mixture was incubated for 30 min. The absorbance was read at 517 nm. Each assay was done in triplicate. Ascorbic acid was used as a positive control.

### Cytotoxicity assay

The green fluorescent protein microplate assay^113^ (GFPMA) was employed to evaluate cytotoxicity against non-cancerous (Vero) cells (African green monkey kidney fibroblasts, ATCC CCL-81). The assay was done in a 384-well plate in quadruplicate. Each well was added with 5 μl of samples and 45 μl of cell suspension. The plate was incubated at 37 °C in a humidified incubator with 5% CO_2_ for 4 days. Fluorescence was measured in the bottom-reading mode with excitation and emission wavelengths at 485 and 535 nm, respectively. % Cytotoxicity was calculated following the equation [1 − [(FU_T_/FU_C_)] × 100, where FU_T_ and FU_C_ were the mean fluorescent unit from cells treated with a test compound and that with 0.5% DMSO, respectively. Less than 50% cytotoxicity was reported as inactive. IC_50_ value is derived from the dose–response-curve that plotted between %cytotoxicity versus the sample concentrations by using SOFTMax Pro software. The maximum tested concentration was done at 1,000 μg/ml. Ellipticine, a reference standard for cytotoxicity against Vero cells.

## Supplementary information

Supplementary information

## Abbreviations

rhAChE: Recombinant human acetylcholinesterase
CH_2_Cl_2_: Dichloromethane
DAP: Diaminopimelic acid
DSM: German Collection of Microorganisms and Cell Cultures GmnH
EtOAc: Ethyl acetate
FT-IR: Fourier transformation infrared spectroscopy
GC: Gas chromatography
ISP: International *Streptomyces* Project
GFPMA: Green fluorescent protein microplate assay
MeOH: Methanol
MS: Mass spectrometry
NBT: Nitroblue tetrazolium
NMR: Nuclear magnetic resonance
NRPS: Non-ribosomal peptide synthetase
smBGCs: Secondary metabolism biosynthetic gene clusters
T1PKS: Type I polyketide synthase
T3PKS: Type III polyketide synthase
TBRC: Thailand Bioresource Research Center

## References

[1] Lewin GR (2016). Evolution and ecology of actinobacteria and their bioenergy applications. Annu. Rev. Microbiol..

[2] Agrawal PK, Agrawal S, Shrivastava R (2015). Modern molecular approaches for analyzing microbial diversity from mushroom compost ecosystem. 3 Biotech..

[3] Yagi A (2017). Anti-*Mycobacterium* activity of microbial peptides in a silkworm infection model with *Mycobacterium smegmatis*. J. Antibiot..

[4] Okujo N (2007). Bispolides, novel 20-membered ring macrodiolide antibiotics from *Microbispora*. J. Antibiot..

[5] Indananda C (2013). Linfuranone A, a new polyketide from plant-derived *Microbispora* sp. GMKU 363. J. Antibiot..

[6] Klafki HW, Staufenbiel M, Kornhuber J, Wiltfang J (2006). Therapeutic approaches to Alzheimer’s disease. Brain.

[7] Lin, M. T. & Beal, M. F. Mitochondrial dysfunction and oxidative stress in neurodegenerative diseases. *Nature.* **443**, 787–795 (2006). 10.1038/nature05292 17051205

[8] Devasagayam TP (2004). Free radicals and antioxidants in human health: current status and future prospects. J. Assoc. Physicians India..

[9] Ohlendorf B, Schulz D, Erhard A, Nagel K, Imhoff JF (2012). Geranylphenazinediol, an acetylcholinesterase inhibitor produced by a *Streptomyces* species. J. Nat. Prod..

[10] Li JL (2015). Acetylcholinesterase inhibitory dimeric indole derivatives from the marine actinomycetes *Rubrobacter radiotolerans*. Fitoterapia.

[11] Almasi F, Mohammadipanah F, Adhami H-R, Hamedi J (2018). Introduction of marine-derived *Streptomyces* sp. UTMC 1334 as a source of pyrrole derivatives with anti-acetylcholinesterase activity. J. Appl. Microbiol..

[12] Zheng ZH (2007). Isolation and characterization of N98–1272 A, B and C, selective acetylcholinesterase inhibitors from metabolites of an actinomycete strain. J. Enzym. Inhib. Med. Chem..

[13] Kim JS, Shin-ya K, Furihata K, Hayakawa Y, Seto H (1997). Structure of mescengricin, a novel neuronal cell protecting substance produced by *Streptomyces griseoflavus*. Tetrahedron Lett..

[14] Hayakawa Y (2010). Flaviogeranin, a new neuroprotective compound from *Streptomyces* sp. J. Antibiot..

[15] Kim JS, Shin-ya K, Eishima J, Furihata K, Seto H (1996). A novel neuronal cell protecting substance, naphthomycinol, produced by *Streptomyces* sp. PF7. J. Antibiot..

[16] Kobayashi H (2001). Neuroprotectins A and B, bicyclohexapeptides protecting chick telencephalic neuronal cells from excitotoxicity. I. Fermentation, isolation, physico-chemical properties and biological activity. J. Antibiot..

[17] Hayakawa Y, Kobayashi T, Izawa M (2013). Indanostatin, a new neuroprotective compound from *Streptomyces* sp. J. Antibiot..

[18] Shin-ya K, Tanaka M, Furihata K, Hayakawa Y, Seto H (1993). Structure of carquinostatin a, a new neuronal cell protecting substance produced by *Streptomyces exfoliatus*. Tetrahedron Lett..

[19] Shin-Ya K (1995). A new neuronal cell protecting substance, lavanduquinocin, produced by *Streptomyces viridochromogenes*. J. Antibiot..

[20] Sukatta U, Rugthaworn P, Punjee P, Chidchenchey S, Keeratinijakal V (2009). Chemical composition and physical properties of oil from Plai (*Zingiber**cassumunar* Roxb.) obtained by hydrodistillation and hexane extraction. Kasetsart J. Natl. Sci..

[21] Jasim B, Joseph AA, John CJ, Mathew J, Radhakrishnan EK (2014). Isolation and characterization of plant growth promoting endophytic bacteria from the rhizome of *Zingiber officinale*. 3 Biotech.

[22] Singh M, Kumar A, Singh R, Pandey KD (2017). Endophytic bacteria: a new source of bioactive compounds. 3 Biotech.

[23] Nonomura H, Ohara Y (1960). Distribution of actinomycetes in soil. IV. The isolation and classification of the genus *Microbispora*. J. Ferment. Technol..

[24] Li C (2015). *Microbispora**bryophytorum* sp. Nov., an actinomycete isolated from moss (*Bryophyta*). Int. J. Syst. Evol. Microbiol..

[25] Han C (2016). *Microbispora**camponoti* sp. Nov., a novel actinomycete isolated from the cuticle of *Camponotus**japonicus* Mayr. Antonie Van Leeuwenhoek.

[26] Nakajima Y, Kitpreechavanich V, Suzuki K, Kudo T (1999). *Microbispora* corallina sp. Nov., a new species of the genus *Microbispora* isolated from Thai soil. Int. J. Syst. Bacteriol..

[27] Boondaeng A, Ishida Y, Tamura T, Tokuyama S, Kitpreechavanich V (2009). *Microbispora**siamensis* sp. Nov., a thermotolerant actinomycete isolated from soil. Int. J. Syst. Evol. Microbiol..

[28] Miyadoh S, Amano S, Tohyama H, Shomura T (1990). A taxonomic review of the genus *Microbispora* and a proposal to transfer two species to the genus *Actinomadura* and to combine ten species into *Microbispora rosea*. J. Gen. Microbiol..

[29] Xu XX (2012). *Microbispora**hainanensis* sp. nov., isolated from rhizosphere soil of Excoecaria agallocha in a mangrove. Int. J. Syst. Evol. Microbiol..

[30] Kittisrisopit S, Pittayakhajonwut P, Tadtong S, Thawai C (2018). *Microbispora**soli* sp. nov., isolated from soil of a hot spring. Int. J. Syst. Evol. Microbiol..

[31] Saitou N, Nei M (1987). The neighbor-joining method: a new method for reconstructing phylogenetic trees. Mol. Biol. Evol..

[32] Felsenstein J (1981). Evolutionary trees from DNA sequences: a maximum likelihood approach. J. Mol. Evol..

[33] Yoon SH (2017). Introducing EzBioCloud: a taxonomically united database of 16S rRNA and whole genome assemblies. Int. J. Syst. Evol. Microbiol..

[34] Rodriguez-R, L. M. & Konstantinidis, K. T. The enveomics collection: a toolbox for specialized analyses of microbial genomes and metagenomes.* PeerJ Preprints*** 4**, e1900v1.

[35] Richter M, Rosselló-Móra R, Glöckner FO, Peplies J (2016). JSpeciesWS: a web server for prokaryotic species circumscription based on pairwise genome comparison. Bioinformatics.

[36] Chun J (2018). Proposed minimal standards for the use of genome data for the taxonomy of prokaryotes. Int. J. Syst. Evol. Microbiol..

[37] Meier-Kolthoff JP, Auch AF, Klenk HP, Göker M (2013). Genome sequence-based species delimitation with confidence intervals and improved distance functions. BMC Bioinform..

[38] Wayne LG (1987). Report of the ad hoc committee on reconciliation of approaches to bacterial systematics. Int. J. Syst. Bacteriol..

[39] Carver TJ (2005). ACT: the Artemis comparison tool. Bioinformatics.

[40] Weber T (2015). AntiSMASH 3.0—a comprehensive resource for the genome mining of biosynthetic gene clusters. Nucleic Acids Res..

[41] Funabashi M, Funa N, Horinouchi S (2008). Phenolic lipids synthesized by type III polyketide synthase confer penicillin resistance on *Streptomyces griseus*. J. Biol. Chem..

[42] Becerril A (2018). Uncovering production of specialized metabolites by *Streptomyces argillaceus*: activation of cryptic biosynthesis gene clusters using nutritional and genetic approaches. PLoS ONE.

[43] Pan G (2017). Discovery of the leinamycin family of natural products by mining actinobacterial genomes. Proc. Natl. Acad. Sci..

[44] Giglio S, Jiang J, Saint CP, Cane DE, Monis PT (2008). Isolation and characterization of the gene associated with geosmin production in cyanobacteria. Environ. Sci. Technol..

[45] Lee GC (2014). Molecular characterization of actinomycetes isolated from terrestrial environment and their synthesis of geosmin and 2-MIB. J. Pure Appl. Microbiol..

[46] Luo Q, Hu H, Peng H, Zhang X, Wang W (2015). Isolation and structural identification of two bioactive phenazines from *Streptomyces griseoluteus* P510. Chin. J. Chem. Eng..

[47] Gao X (2012). A novel anticancer and antifungus phenazine derivative from a marine actinomycete BM-17. Microbiol. Res..

[48] Gutierrez-Lugo MT (2005). Isolation of three new naturally occurring compounds from the culture of *Micromonospora* sp. P1068. Nat. Prod. Res..

[49] Hwang BK, Lim SW, Kim BS, Lee JY, Moon SS (2001). Isolation and in vivo and in vitro antifungal activity of phenylacetic acid and sodium phenylacetate from *Streptomyces humidus*. Appl. Environ. Microbiol..

[50] Dominic A (2018). Biologically active phenolic acids produced by *Aspergillus* sp., an endophyte of *Moringa**oleifera*. Eur. J. Biol. Res..

[51] Ohtani K, Fujioka S, Kawano T, Shimada A, Kimura Y (2011). Nematicidal activities of 4-hydroxyphenylacetic acid and oidiolactone D produced by the fungus *Oidiodendron* sp. Z. Naturforsch. C. J. Biosci..

[52] Jiang K, Yang SX (2013). Chemical constituents from marine *Streptomycete* sp. S11. Appl. Mech. Mater..

[53] Evidente A, Iacobellis NS, Sisto A (1993). Isolation of indole-3-acetic acid methyl ester, a metabolite of indole-3-acetic acid from *Pseudomonas amygdali*. Experientia.

[54] Tu WC (2019). Diterpenoids and sesquiterpenoids from the stem bark of *Metasequoia glyptostroboides*. Phytochem..

[55] Kerr JR (1999). *Pseudomonas aeruginosa* pyocyanin and 1-hydroxyphenazine inhibit fungal growth. J. Clin. Pathol..

[56] Breitmaier E, Hollstein U (1976). Carbon-13 nuclear magnetic resonance chemical shifts of substituted phenazines. J. Org. Chem..

[57] Akabori H, Nakamura M (1959). 1,6-Dihydroxyphenazine, an antibiotic produced by *Streptomyces thioluteus*. J. Antibiot..

[58] Lu CH, Li YY, Wang HX, Wang BM, Shen YM (2013). A new phenoxazine derivative isolated from marine sediment actinomycetes, *Nocardiopsis* sp. 236. Drug. Discov. Ther..

[59] Supong K, Thawai C, Supothina S, Auncharoen P, Pittayakhajonwut P (2016). Antimicrobial and anti-oxidant activities of quinoline alkaloids from *Pseudomonas aeruginosa* BCC76810. Phytochem. Lett..

[60] Myo EM (2019). Indole-3-acetic acid production by *Streptomyces fradiae* NKZ-259 and its formulation to enhance plant growth. BMC Microbiol..

[61] Savia DC (2015). *Microbispora* sp. LGMB259 endophytic actinomycete isolated from *Vochysia**divergens* (Pantanal, Brazil) producing β-carbolines and indoles with biological activity. Curr. Microbiol..

[62] Aoki Y (2005). Anthranilic acid, a spore germination inhibitor of phytopathogenic *Streptomyces* sp. B-9-1 causing root tumor of melon. Actinomycetologica.

[63] Gohlke H, Hendlich M, Klebe G (2000). Knowledge-based scoring function to predict protein-ligand interactions. J. Mol. Biol..

[64] Liu K, Kokubo H (2017). Exploring the stability of ligand binding modes to proteins by molecular dynamics simulations: a cross-docking study. J. Chem. Inf. Model..

[65] Roca C (2018). Identification of new allosteric sites and modulators of AChE through computational and experimental tools. J. Enzym. Inhib. Med. Chem..

[66] Daina A, Michielin O, Zoete V (2017). SwissADME: a free web tool to evaluate pharmacokinetics, drug-likeness and medicinal chemistry friendliness of small molecules. Sci. Rep..

[67] Goldberg JS (2011). Low molecular weight opioid peptide esters could be developed as a new class of analgesics. Perspect. Med. Chem..

[68] Fernandesa TB, Segrettib MCF, Pollic MC, Parise-Filho R (2016). Analysis of the applicability and use of Lipinski’s rule for central nervous system drugs. Lett. Drug Des. Discov..

[69] Doan MKM (2002). Passive permeability and P-glycoprotein-mediated efflux differentiate central nervous system (CNS) and non-CNS marketed drugs. J. Pharmacol. Exp. Ther..

[70] Leeson PD, Davis AM (2004). Time-related differences in the physical property profiles of oral drugs. J. Med. Chem..

[71] Van de Waterbeemd, H., Camenisch, G., Folkers, G., Chretien, J. R. & Raevsky, O. A. Estimation of blood−brain barrier crossing of drugs using molecular size and shape, and H-bonding descriptors. *J. Drug. Target.* **6**, 151−165 (1998). 10.3109/10611869808997889 9886238

[72] Pajouhesh H, Lenz GR (2005). Medicinal chemical properties of successful central nervous system drugs. NeuroRx..

[73] Daina A, Zoete VA (2016). BOILED-Egg to predict gastrointestinal absorption and brain penetration of small molecules. Chem. Med. Chem..

[74] Shirling EB, Gottlieb D (1966). Methods for characterization of *Streptomyces* species. Int. J. Syst. Bacteriol..

[75] Kelly KL (1964). Inter-Society Color Council—National Bureau of Standard Color Name Charts Illustrated with Centroid Colors.

[76] Arai T (1975). Culture Media for Actinomycetes.

[77] Williams ST, Cross T, Booth C (1971). Actinomycetes. Methods in Microbiology.

[78] Gordon RE, Barnett DA, Handerhan JE, Pang CHN (1974). *Nocardia coeliaca*, *Nocardia autotrophica*, and the nocardia strain. Int. J. Syst. Bacteriol..

[79] Hasegawa T, Takizawa M, Tanida S (1983). A rapid analysis for chemical grouping of aerobic actinomycetes. J. Gen. Appl. Microbiol..

[80] Komagata K, Suzuki KI (1987). Lipid and cell-wall analysis in bacterial systematics. Methods Microbiol..

[81] Collins MD, Pirouz T, Goodfellow M, Minnikin DE (1977). Distribution of menaquinones in actinomycetes and *corynebacteria*. J. Gen. Microbiol..

[82] Minnikin DE (1984). An integrated procedure for the extraction of bacterial isoprenoid quinones and polar lipids. J. Microbiol. Methods.

[83] Sasser M (1990). Identification of bacteria by gas chromatography of cellular fatty acids MIDI. Technical Note 101.

[84] Kämpfer P, Kroppenstedt RM (1996). Numerical analysis of fatty acid patterns of coryneform bacteria and related taxa. Can. J. Microbiol..

[85] Tamaoka J, Goodfellow M, O’Donnell AG (1994). Determination of DNA base composition. Chemical methods in prokaryotic systematics.

[86] Weisburg WG, Barns SM, Pelletier DA, Lane DJ (1992). 16S ribosomal DNA amplification for phylogenetic study. J. Bacteriol..

[87] Lane DJ, Stackebrandt E, Goodfellow M (1991). 16S/23S rRNA sequencing. Nucleic Acid Techniques in Bacterial Systematics.

[88] Hall TA (1999). BioEdit: a user-friendly biological sequence alignment editor and analysis program for Windows 95/98/NT. Nucleic Acids Symp Ser..

[89] Tamura K, Stecher G, Peterson D, Filipski A, Kumar S (2013). MEGA6: Molecular Evolutionary Genetics Analysis Version 6.0. Mol. Biol. Evol..

[90] Kimura M (1980). A simple method for estimating evolutionary rates of base substitutions through comparative studies of nucleotide sequences. J. Mol. Evol..

[91] Felsenstein J (1985). Confidence limits on phylogenies: an approach using the bootstrap. Evolution.

[92] Bankevich A (2012). SPAdes: a new genome assembly algorithm and its applications to single-cell sequencing. J. Comput. Biol..

[93] Seemann T (2014). Prokka: rapid prokaryotic genome annotation. Bioinformatics.

[94] Ritcher M, Rosselló-Móra R (2009). Shifting the genomics gold standard for the prokaryotic species definition. Proc. Natl. Acad. Sci. USA.

[95] Yoon SH, Ha SM, Lim JM, Kwon SJ, Chun J (2017). A large-scale evaluation of algorithms to calculate average nucleotide identity. Antonie Van Leeuwenhoek.

[96] Meier-Kolthoff JP, Göker M (2019). TYGS is an automated high-throughput platform for state-of-the-art genome-based taxonomy. Nat. Commun..

[97] Stothard P, Wishart DS (2005). Circular genome visualization and exploration using CGView. Bioinformatics.

[98] Ellman GL, Courtney KD, Andres V, Featherstone RM (1961). A new and rapid colorimetric determination of acetylcholinesteraseactivity. Biochem. Pharmacol..

[99] Komersova A, Komers K, Čegan A (2007). New findings about Ellman’s method to determine cholinesterase activity. Naturforsch..

[100] BIOVIA, Discovery Visualizer (v.20.1.0.19295). San Diego (2020).

[101] Cheung J (2012). Structures of human acetylcholinesterase in complex with pharmacologically important ligands. J. Med. Chem..

[102] Hetényi C, van der Spoel D (2002). Efficient docking of peptides to proteins without prior knowledge of the binding site. Protein Sci..

[103] Plewczynski D, Lziniewski M, Augustyniak R, Ginalski K (2011). Can we trust docking results? Evaluation of seven commonly used programs on PDBbind database. J. Comput. Chem..

[104] Morris GM (2009). Autodock 4 and AutoDockTools 4: automated docking with selective receptor flexiblity. J. Comput Chem..

[105] Phillips JC (2005). Scalable molecular dynamics with NAMD. J. Comput. Chem..

[106] Vanommeslaeghe K (2010). CHARMM General Force Field (CGenFF): A force field for drug-like molecules compatible with the CHARMM all-atom additive biological force fields. J. Comput. Chem..

[107] Feller SE, Zhang Y, Pastor RW, Brooks BR (1995). Constant pressure molecular dynamics simulation: the Langevin piston method. J. Chem. Phys..

[108] Darden T, York D, Pedersen L (1993). Particle mesh Ewald: an N.log(N) method for Ewald sums in large systems. J. Chem. Phys..

[109] Ryckaert J-P, Ciccotti G, Berendsen HJ (1977). Numerical integration of the cartesian equations of motion of a system with constraints: molecular dynamics of n-alkanes. J. Comput. Phys..

[110] Humphrey W, Dalke A, Schulten K (1996). VMD-visual molecular dynamics. J. Mol. Graph..

[111] Tadtong S, Kanlayavattanakul M, Laurith N (2013). Neuritogenic and neuroprotective activities of fruit residues. Nat. Prod. Commun..

[112] Brand-Williams W, Cuvelier ME, Berset C (1995). Useofafree radical method to evaluate antioxidant activity. Leben. Wissens. Technol..

[113] Changsen C, Franzblau SG, Palittapongarnpim P (2003). Improved green fluorescent protein reporter gene-based microplate screening for antituberculosis compounds by utilizing an acetamidase promoter. Antimicrob. Agents Chemother..

