# Supplementary material for: A novel diterpene agent isolated from Microbispora hainanensis strain CSR-4 and its in vitro and in silico inhibition effects on acetylcholine esterase enzyme
Source: Sci Rep. 2020 Jul 6;10:11058. doi: 10.1038/s41598-020-68009-y (PMC7338456; doi:10.1038/s41598-020-68009-y)
Supplement: Supplementary file 1 — Supplementary information [file 41598_2020_68009_MOESM1_ESM.pdf]

Scientific Reports (Additional information)

A novel diterpene agent isolated from *Microbispora hainanensis* strain CSR-4 and its *in vitro* and *in silico* inhibition effects on acetylcholine esterase enzyme

Chitti Thawai[1,2,3,*], Nantiya Bunbamrung[4], Pattama Pittayakhajonwut[4], Sumet Chongruchiroj[5], Jaturong Pratuangdejkul[5], Ya-Wen He[6], Sarin Tadtong[7], Vipaporn Sareedenchai[7], Pinidphon Prombutara[8], Yang Qian[9]

Affiliation

[1]Department of Biology, Faculty of Science, King Mongkut's Institute of Technology Ladkrabang, Bangkok 10520, Thailand

[2]Actinobacterial Research Unit, Faculty of Science, King Mongkut's Institute of Technology Ladkrabang, Bangkok 10520, Thailand

[3]Center of Excellence in Applied Biosciences, King Mongkut's Institute of Technology Ladkrabang, Bangkok 10520, Thailand

[4]National Center for Genetic Engineering and Biotechnology (BIOTEC), National Science and Technology Development Agency (NSTDA), Thailand Science Park, Phaholyothin road, Klong Luang, Pathum Thani, 12120 Thailand

[5]Department of Microbiology, Faculty of Pharmacy, Mahidol University, Phayathai Bangkok 10400, Thailand.

[6]State Key Laboratory of Microbial Metabolism, School of Life Sciences & Biotechnology, Shanghai Jiao Tong University, Shanghai 200240, P R China

[7]Department of Pharmacognosy, Faculty of Pharmacy, Srinakharinwirot University, Nakhon nayok 26120, Thailand

[8]Omics Sciences and Bioinformatics Center, Faculty of Science, Chulalongkorn University

254 Payathai Road, Patumwan, Bangkok 10330, Thailand

[9]Department of Life Science and Engineering, Harbin Institute of Technology, Harbin 150001,

P R China

***Correspondence**

Assoc. Prof. Dr. Chitti Thawai, Department of Biolog6y, Faculty of Science, King Mongkut's Institute of Technology Ladkrabang, Bangkok 10520, Thailand

E-mail: chitti.th@kmitl.ac.th   Phone: +66(2)3298400 ext 235   Fax: +66(2)3298427

Additional information

Table S1 Phenotypic characteristics of strain CSR-4 and the closest phylogenetically relative, *M. hainanensis* DSM 45428^T. +, Positive; -, Negative; w, Weakly positive.

Characteristics	*Microbispora* sp. CSR-4	*M. hainanensis* DSM 45428^T
Starch hydrolysis	-	+
Urease activity	+	-
Maximum NaCl tolerance (%w/v)	1	1
The pH range for growth	5-10	5-10
Acid production from:		
D-ribose	+	w
D-Galactose	+	w
D-Glucose	+	+
D-Lactose	w	w
L-Raffinose	-	-
D-Cellobiose	+	+
D-mannitol	+	w
D-fructose	+	+
D-xylose	+	w
Nitrogen utilization:		
L-Methionine	+	w
DL-2-aminobutyric acid	-	-
4-Hydroxyproline	-	+
L-Cysteine	+	w
L-Asparagine	+	+
L-histidine	+	w
L-serine	+	+
L-Valine	-	-
L-Arginine	+	+
L-Phenylalanine	+	-
Decomposition of:		
Tyrosine	-	w
CM-cellulose	-	-
Hypoxanthine	-	+
Xanthine	-	-
Adenine	-	-

Table S2 Cellular fatty acid composition of strain TBRC 10616 and *M. hainanensis* DSM 45428^T

Fatty acids	Strain TBRC 10616	*M. hainanensis* DSM 45428^T
**Saturated fatty acids**		
$C_{12:0}$	0.2	3.5
$C_{13:0}$	0.1	-
$C_{14:0}$	1.0	2.0
$C_{15:0}$2-OH	0.1	5.7
$C_{16:0}$	14.6	20.6
$C_{16:0}$2-OH	0.5	1.0
$C_{17:0}$	4.3	6.3
$C_{17:0}$3-OH	0.2	-
$C_{18:0}$	3.7	4.6
$C_{19:0}$	0.1	-
**Unsaturated fatty acids**		
$C_{15:1}$ ω6c	0.1	1.4
$C_{17:1}$ ω8c	3.3	-
$C_{18:1}$ ω9c	2.0	1.7
**Branched fatty acids**		
iso-$C_{14:0}$	0.5	1.1
iso-$C_{15:0}$	3.2	5.3
anteiso-$C_{15:0}$	2.1	2.2
iso-$C_{16:0}$	32.1	19.7
iso-$C_{16:1}$ H	0.4	-
iso-$C_{17:0}$	2.1	2.7
anteiso-$C_{17:0}$	7.4	3.2
iso-$C_{18:0}$	1.3	-
anteiso-$C_{19:0}$	0.1	-
$C_{16:0}$ 10-methyl	6.5	6.9
$C_{17:0}$ 10-methyl	7.4	5.3
$C_{18:0}$ 10-methyl, TBSA	1.6	2.6

Table S3. ANI (Kostas Lab), ANIm and dDDH values among the draft genomes of strain CSR-4 and *M. hainanensis* DSM 45428^{T}, the closest relative.

Query genome	Reference genome	ANI (%) From Kostas lab	ANIm (%)	Digital DNA-DNA hybridization relatedness												G+C difference
				Formula 1*				Formula 2**				Formula 3***				
				% dDDH	Model C.I. (%)	Distance	Prob. DDH >= 70 (same species)	% dDDH	Model C.I. (%)	Distance	Prob. DDH >= 70 (same species)	% dDDH	Model C.I. (%)	Distance	Prob. DDH >= 70 (same species)	
CSR-4	*M. hainanensis* DSM 45428^{T}	95.34	95.06	74.7	70.7 - 78.3	0.1591	84.04	58.40	55.6-61.1	0.0545	46.87	73.80	70.3-77	0.2049	85.8	0.03

*Formula 1: a formula (HSP length / total length), **Formula 2: a formula (identities/HSP length), ***Formula 3: a formula (identities / total length) which are liberated of genome length and are thus prosperous against the use of incomplete draft genomes.

Table S4. General features of the genome sequences of the strain CSR-4 and *M. hainanensis* DSM 45428^T

Features	*Microbispora* sp. CSR-4	*M. hainanensis* DSM 45428^T
Bioproject	PRJNA552279	PRJNA552327
Accession no.	VJVX00000000	VIRM00000000
Sequencing Technology	Illumima MiSeq	Illumima MiSeq
Genome coverage	102x	95x
N50	113,205	121,286
Number of Contigs	354	251
Genome size (bp)	8,666,412	8,717,787
DNA G+C content (%)	71.30	71.26
Number of genes	7,945	8,061
Protein coding genes	7,825	7,692
rRNA	9	4
tRNA	76	57
tmRNA	1	1
misc_RNA	34	0
Total	25	20

Table S5. Numbers of secondary metabolism gene clusters in the genomes of *Microbispora* sp. CSR-4 and *M. hainanensis* DSM 45428^T

Secondary metabolism gene cluster	*Microbispora* sp. CSR-4	*M. hainanensis* DSM 45428^T
Type I PKS	4	1
Type II PKS	0	0
Type III PKS	1	2
NRPS	4	4
NRPS-like fragment	2	2
Bacteriocin	1	0
Linear azol(in)e-containing peptides (LAP)	1	1
Siderophore	2	2
Terpene	6	6
Butyrolactone	1	0
Ectoine	1	0
Phenazine	1	1
Betalactone	1	0
Thiopeptide	0	1
Total	25	20

Table S6. Antioxidant and anti-acetylcholinesterase activities of compound 1

	Antioxidant		Antiacetylcholinesterase (using recombinant human AChE)		Cytotoxicity[a] (Vero cell)
	% inhibition (at 100 μg/mL)	IC_{50} (μg/mL)	% inhibition (at 100 μg/mL)	IC_{50} (μg/mL)	IC_{50} (μg/ml)
Compound 1	9.72 ± 2.09	>1000	52.81 ± 1.24	96.87 ± 2.31	>1000
Positive control	Ascorbic acid: 93.41 ± 2.21	Ascorbic acid: IC_{50} 10.12 ± 1.84	Galanthamine: 88.81 ± 1.32	Galanthamine: 1.03 ± 1.97	Ellipticine: 1.12 ± 0.84

Values are mean ± SEM (n = 3)

[a]Maximum tested concentration was at 1000.00 μg/ml.

Figure S1 Maximum-Likelihood (ML) phylogenetic tree based on 16S rRNA gene sequences comparing strain CSR-4 to *Microbispora* species and other genera in the family *Streptosporangiaceae*. *Actinomadura madurae* JCM 7436^T was used as the out-group. The numbers on the branches indicate the percentage bootstrap values of 1,000 replicates.

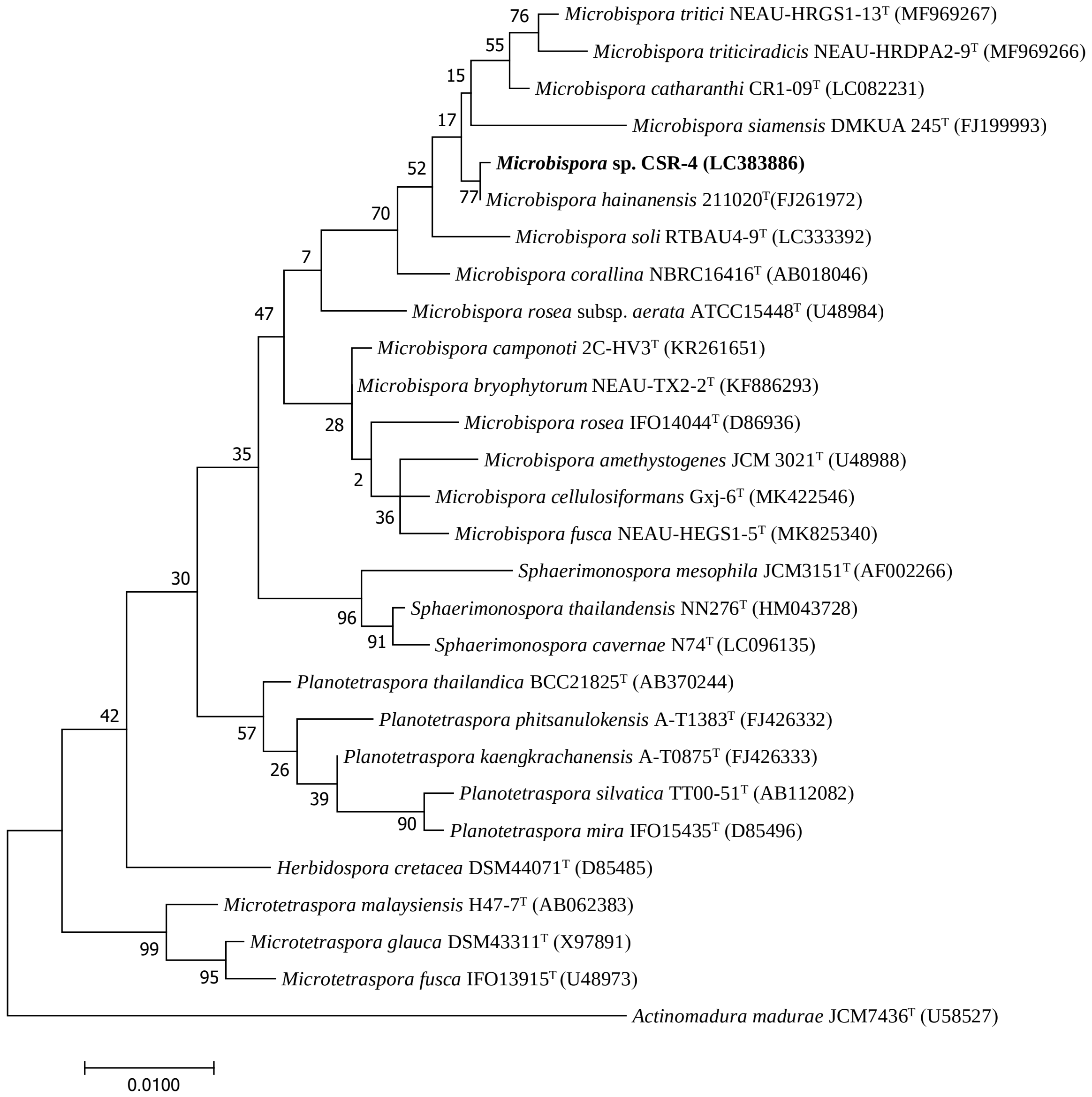

Figure S2 The phylogenomic tree of strain CSR-4 and their related type strains of the genus *Microbispora* obtained from TYGS. Tree inferred with FastME 2.1.6.1[1] from GBDP distances calculated from genome sequences. The branch lengths are scaled in terms of GBDP distance formula *d5*. The numbers above branches are GBDP pseudo-bootstrap support values > 60 % from 100 replications, with an average branch support of 96.3 %. The tree was rooted at the midpoint[2].

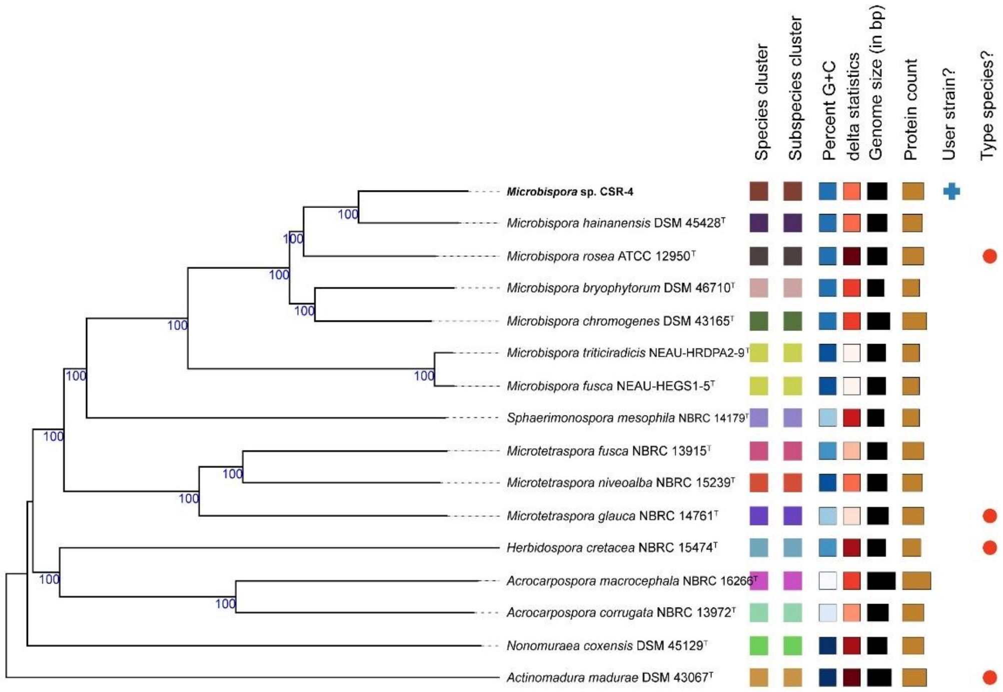

1. Lefort V, Desper R, Gascuel O. FastME 2.0: A comprehensive, accurate, and fast distance-based phylogeny inference program. Mol Biol Evol. 2015;32: 2798–2800. DOI: 10.1093/molbev/msv150.

2. Farris JS. Estimating phylogenetic trees from distance matrices. Am Nat. 1972;106: 645–667.

Figure S3 Pairwise genome alignments comparing the genomes of *Microbispora* sp. CSR-4 and *Microbispora hainanensis* DSM 45428^{T}. The Artemis Comparison Tool (ACT) was used to compare the two genome sequences against each other. Genome sequences were aligned and visualized in ACT with a cut-off set to blast scores >500. Red and blue bars indicate regions of similarity in the same orientation (red) and inverted (blue).

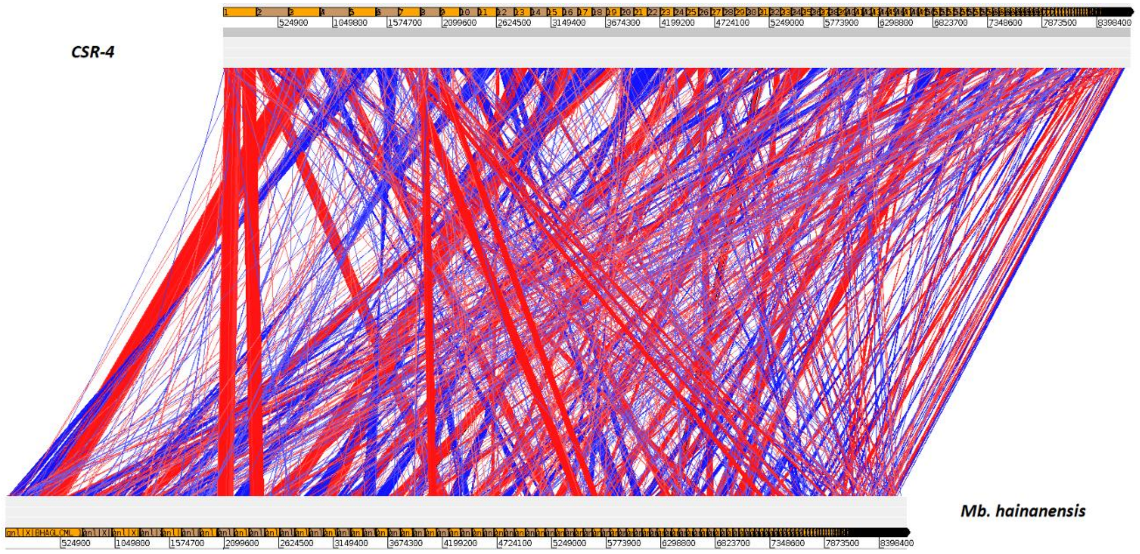

Fig. S4 a) Colony morphology and b) Scanning electron micrograph of strain CSR-4 grown on ISP2 agar for 3 weeks at 30°C. Bar, 5 μm.

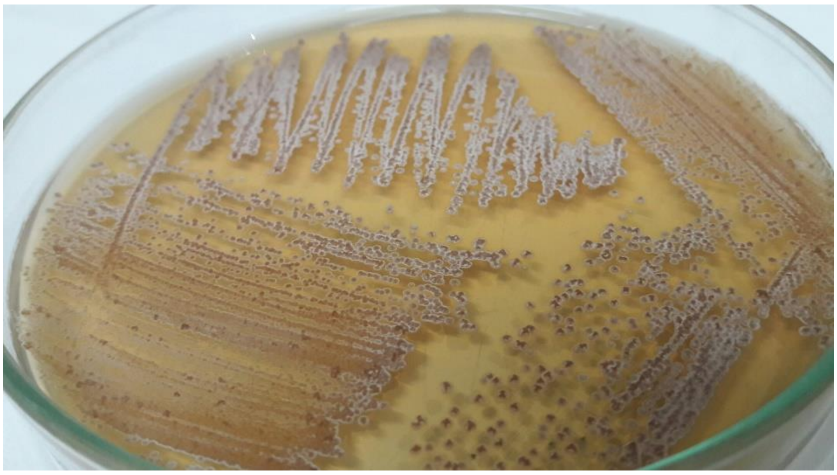

Fig. S4a

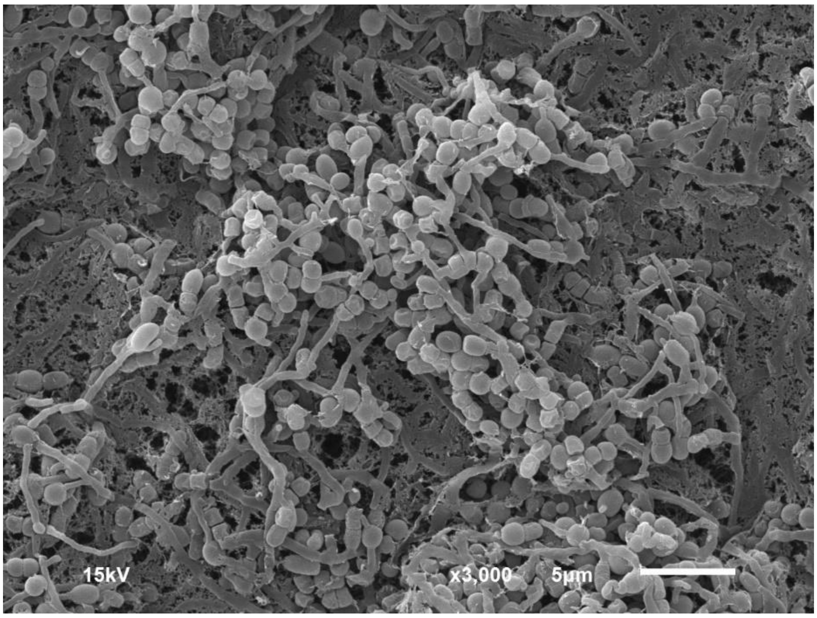

Fig. S4b

Fig. S5 Circular genome map of strain CSR-4. The genome map was made using CGView (Stothard and Wishart, 2005) with COG function annotation. From outside to inside, circle 1, the outer scale is numbered in intervals of 500 Kbp; circles 2 and 3 display the distribution of genes related to COG categories (different colors represent different COG function classification) and the predicted protein-coding genes in the forward strand, respectively; circle 4, the separation line; circles 5 and 6 display the predicted protein-coding genes and the distribution of genes related to COG categories in the reverse strand, respectively.

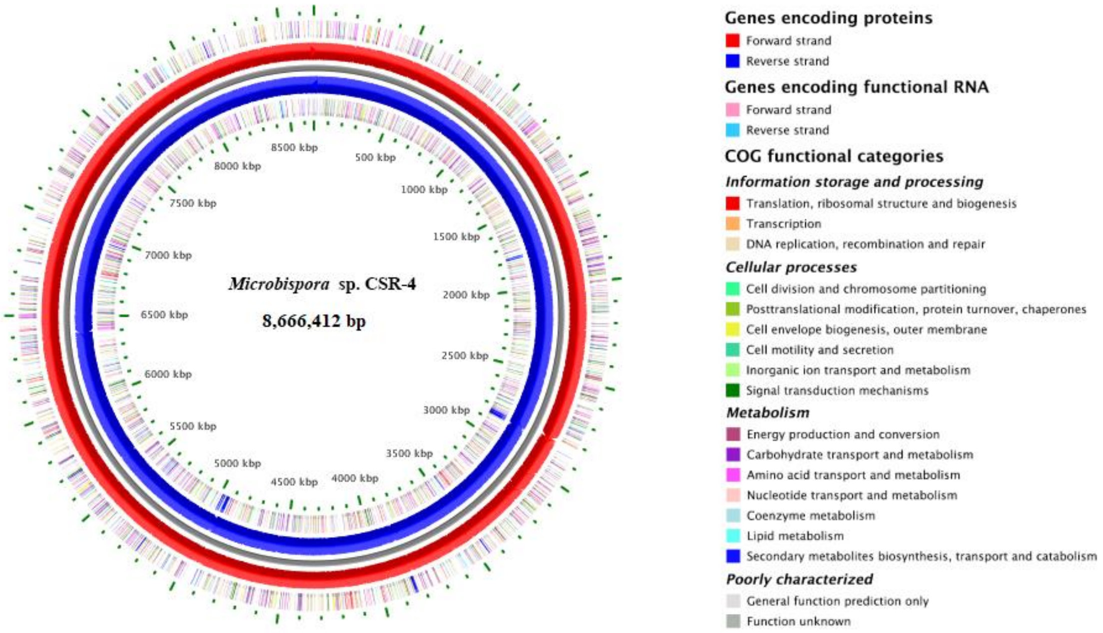

Chemical structure details

2α-Hydroxy-8(14),15-pimaradien-17,18-dioic acid (compound **1**)

Brown oil; $[\alpha]_D^{25}$ +25.65 (*c* 0.16, MeOH); UV λ_{max}, nm (log ε, MeOH) 233 (3.26), 267 (3.21); IR (ATR) ν_{max}, cm^{-1} 3600-2800 (br), 2925, 2854, 1698, 1468, 1391, 1371, 1252, 1233, 1150, 1129, 1030, 1008, 964 and 923 (Fig. S4); ^1H (500 MHz) and ^{13}C NMR (125 MHz) data in CD_3OD; HRESIMS *m/z* $[M + Na]^+$: 371.1826 (calcd for $C_{20}H_{28}O_5Na$, 371.1829).

Figure S6 HRESIMS spectrum of 2α-hydroxy-8(14),15-pimaradien-17,18-dioic acid (compound 1)

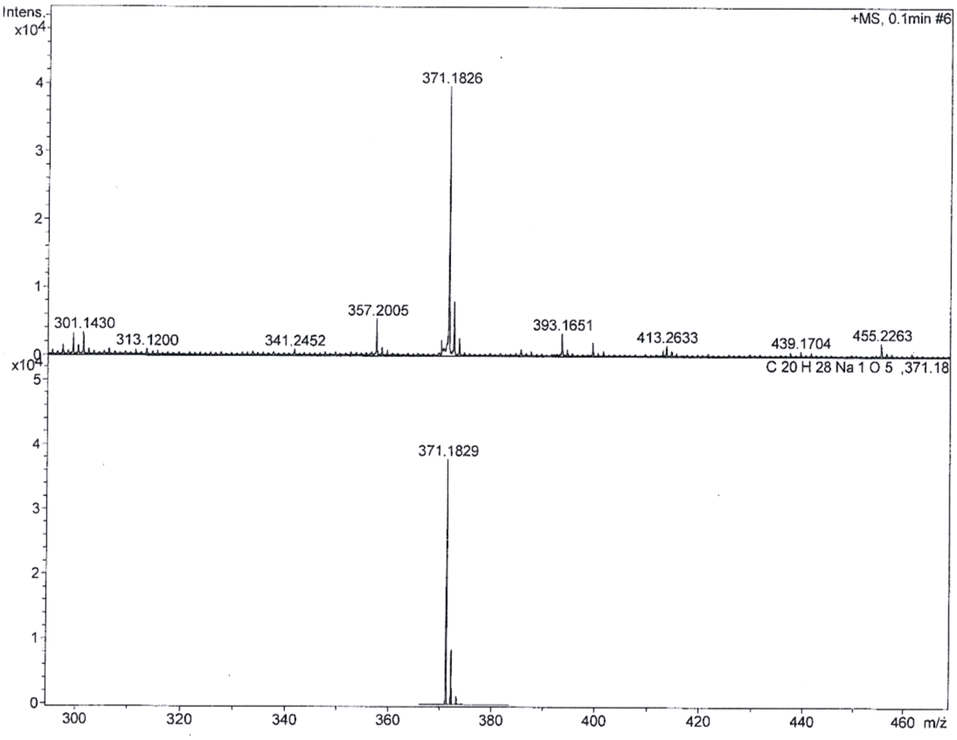

Figure S7 IR spectrum of 2α-hydroxy-8(14),15-pimaradien-17,18-dioic acid (compound 1)

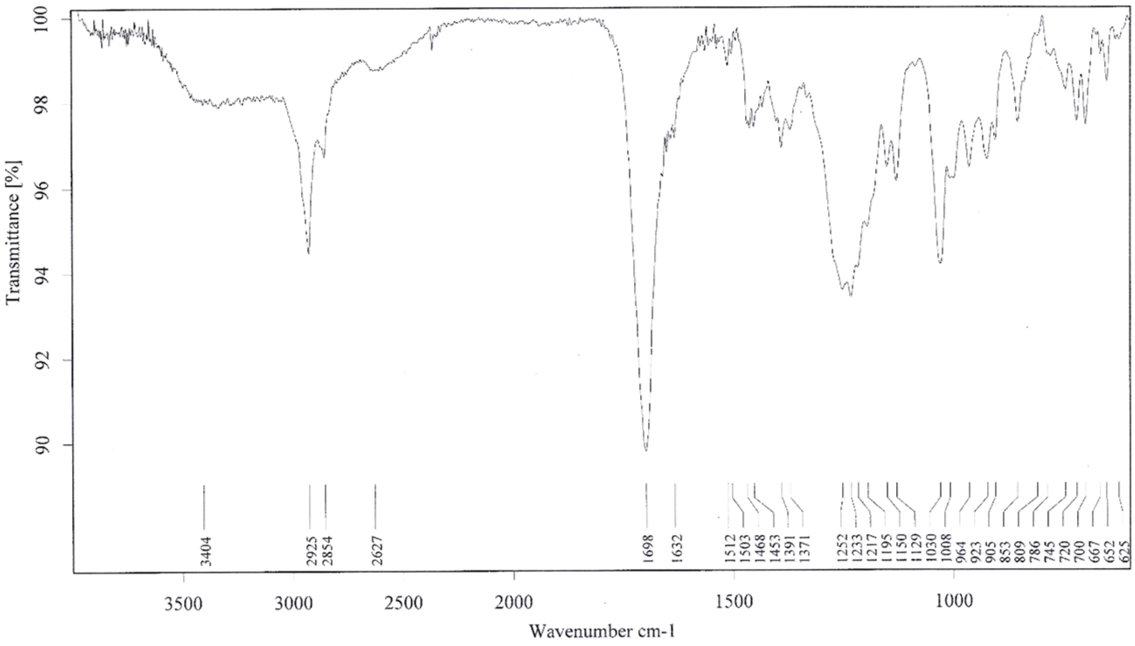

Figure S8 ^{1}H NMR spectrum of 2α-hydroxy-8(14),15-pimaradien-17,18-dioic acid (compound 1) in CD_3OD (500 MHz)

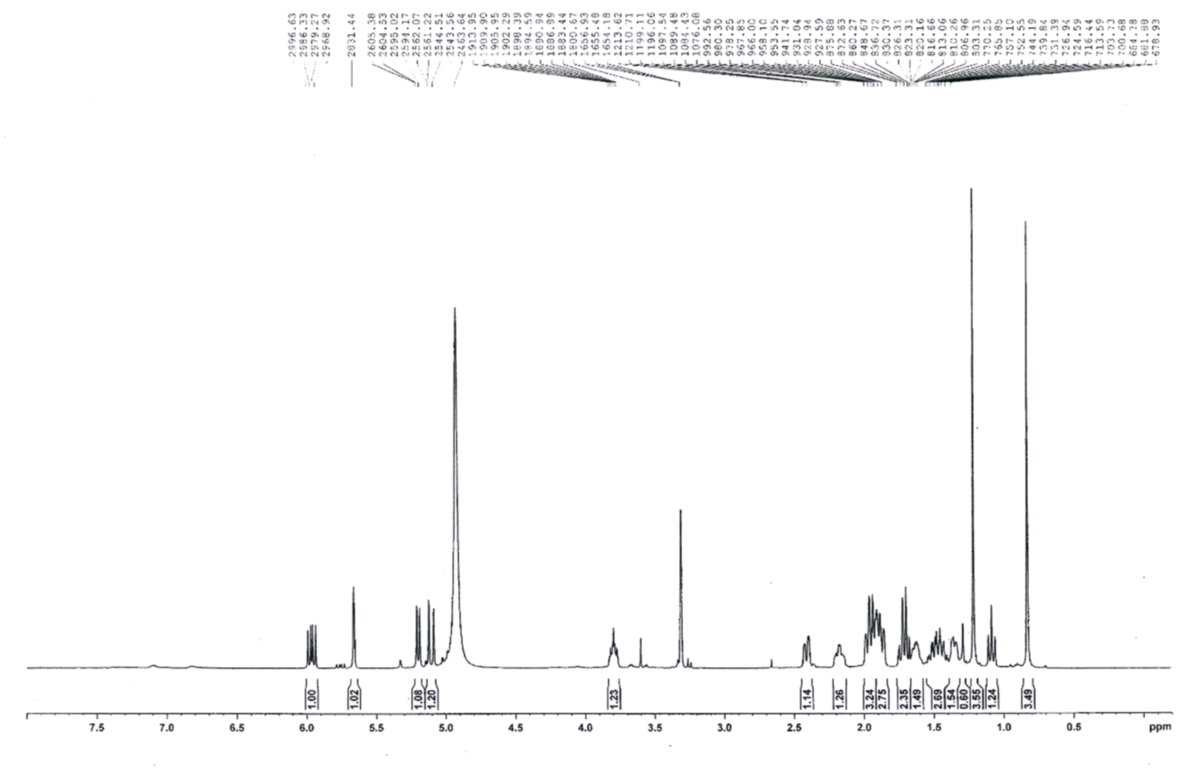

Figure S9 ^{13}C NMR spectrum of 2α-hydroxy-8(14),15-pimaradien-17,18-dioic acid (compound 1) in CD_3OD (125 MHz)

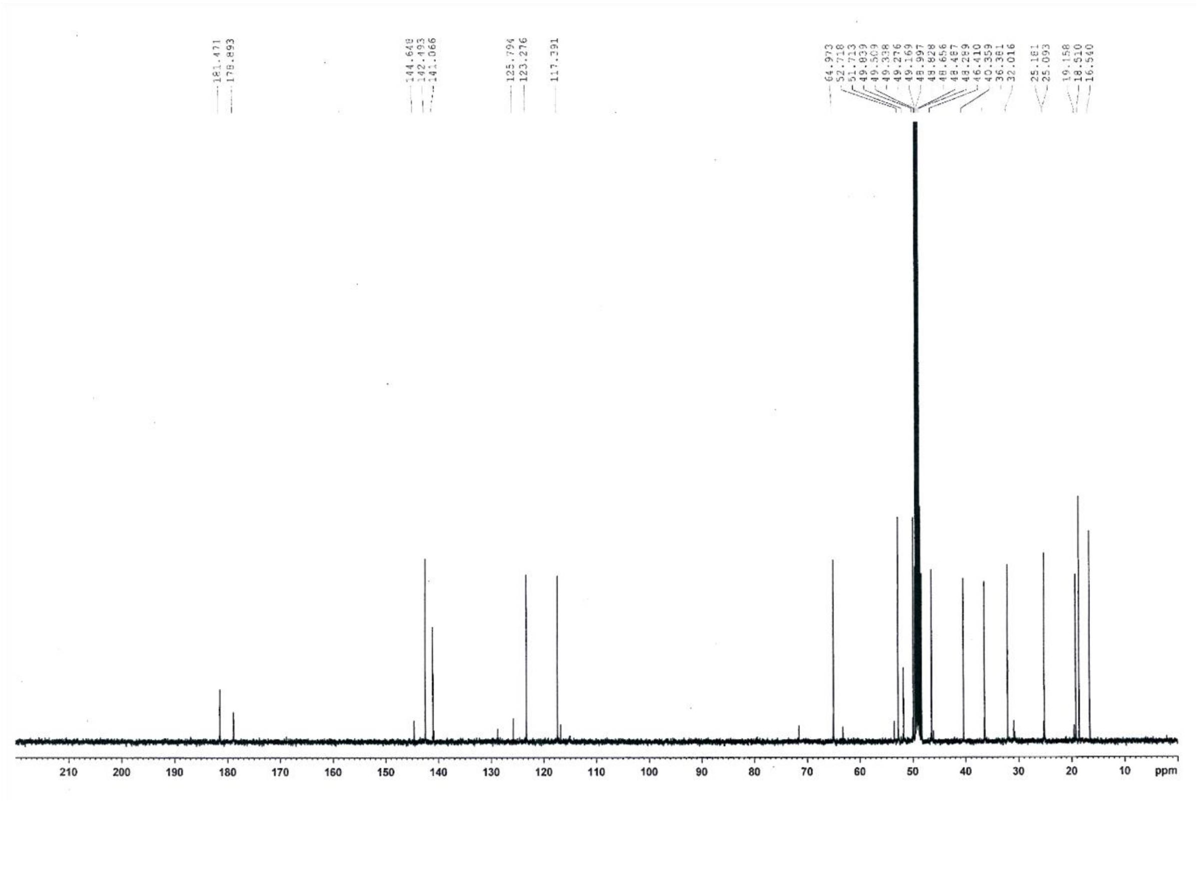

*1-Hydroxyphenazine (compound **2**)*

Brown solid; ^{1}H-NMR (500 MHz, CD_3OD) 7.22 (1H, d, *J* = 7.45 Hz), 7.72 (1H, d, *J* = 8.75 Hz), 7.81 (1H, t, *J* = 6.88 Hz), 7.92 (1H, d, *J* = 8.75 Hz), 7.95, (1H, t, *J* = 6.70 Hz), 8.21 (1H, d, *J* = 8.20 Hz), 8.35 (1H, d, *J* = 8.21 Hz); ^{13}C NMR (125 MHz, CD_3OD) 111.2 (CH), 120.1 (CH), 129.8 (CH), 130.6 (CH), 131.6 (CH), 132.4 (CH), 133.4 (CH), 137.2 (C), 143.1 (C), 144.5 (C), 145.0 (C), 154.5 (C); HRESIMS *m/z* 197.0715 $[M+H]^+$ (calcd for $C_{12}H_9N_2O$, 197.0709).

Figure S10 HRESIMS of compound **2**

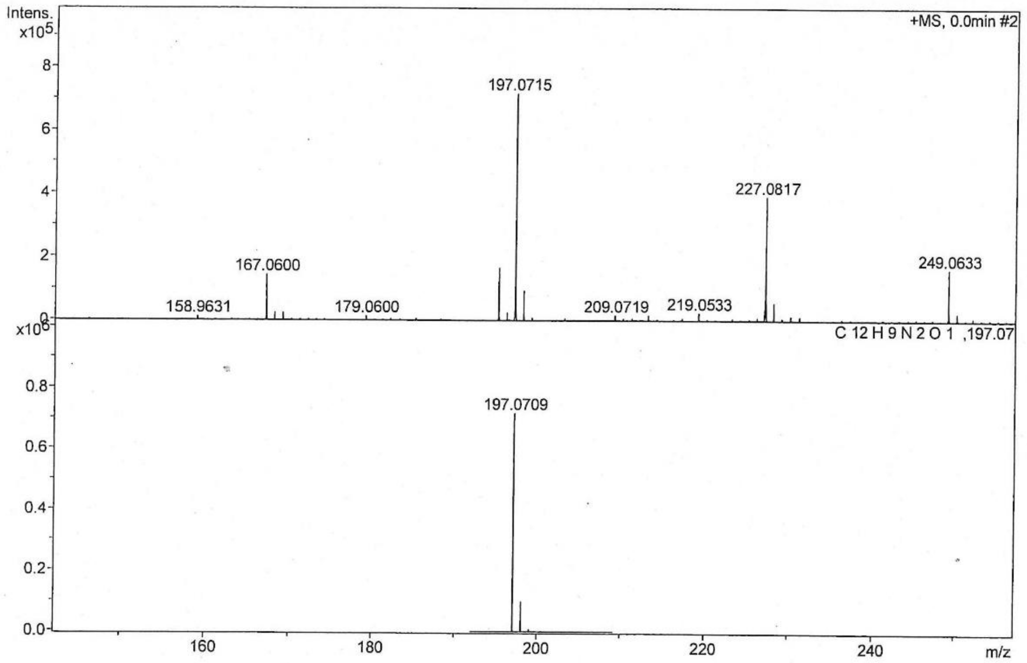

Figure S11 ^{1}H NMR spectrum of compound **2** in CD_3OD (500 MHz)

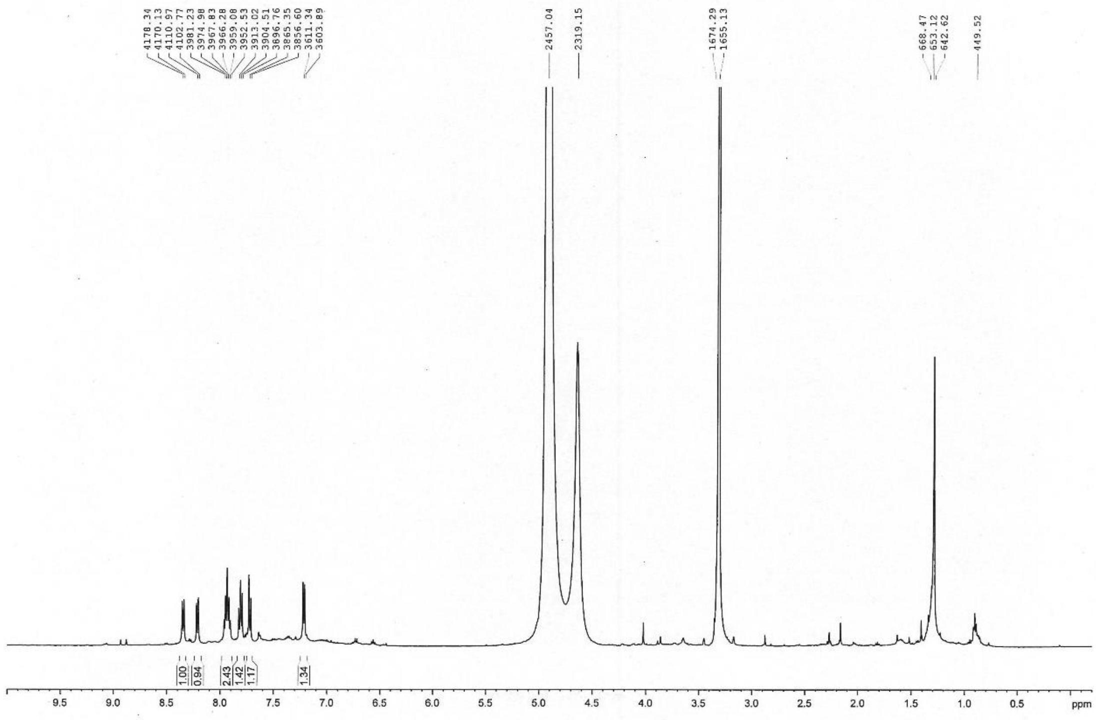

Figure S12 ^{13}C NMR spectrum of compound **2** in CD_3OD (125 MHz)

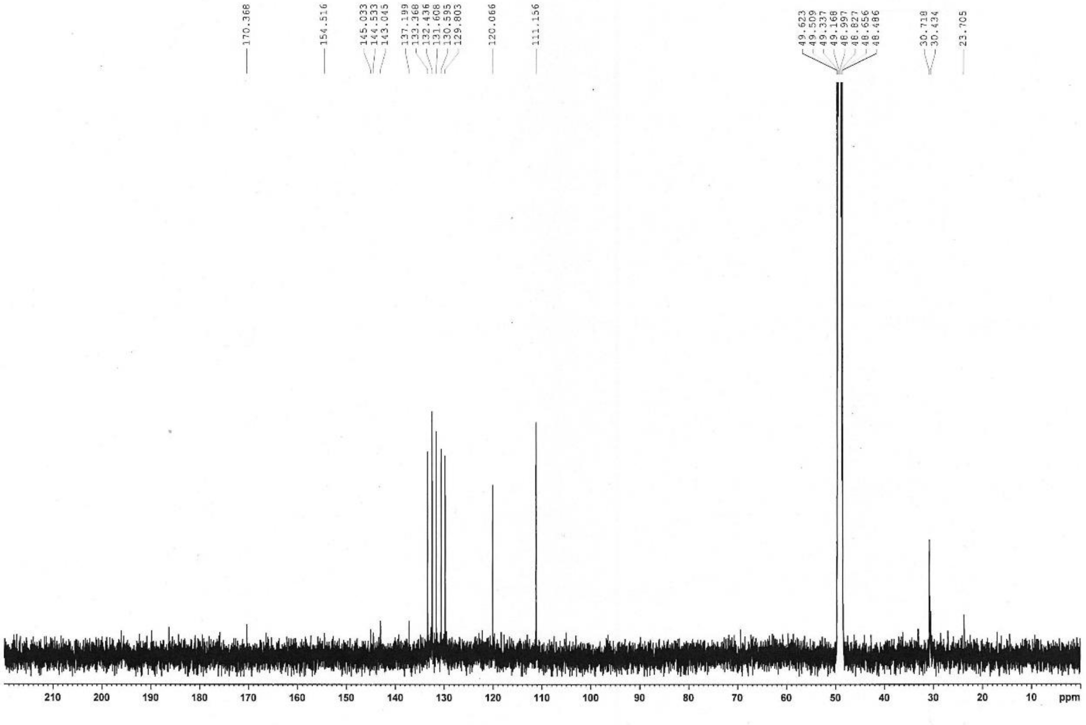

*1, 6-dihydroxylphenazine (compound **3**)*

Dark yellow solid; ^{1}H-NMR (400 MHz, CD_3OD) 7.26 (2H, d, *J* = 7.35 Hz), 7.73 (2H, d, *J* = 8.56 Hz), 7.83 (2H, t, *J* = 8.10 Hz), 9.33 (2H, s); ^{13}C NMR (100 MHz, CD_3OD) 110.5 (CH), 120.2 (CH), 132.6 (CH), 136.5 (C), 142.8 (C), 153.8 (C); HRESIMS *m/z* 213.0657 $[M+H]^+$ (calcd for $C_{12}H_9N_2O_2$, 213.0659).

Figure S13 HRESIMS of compound **3**

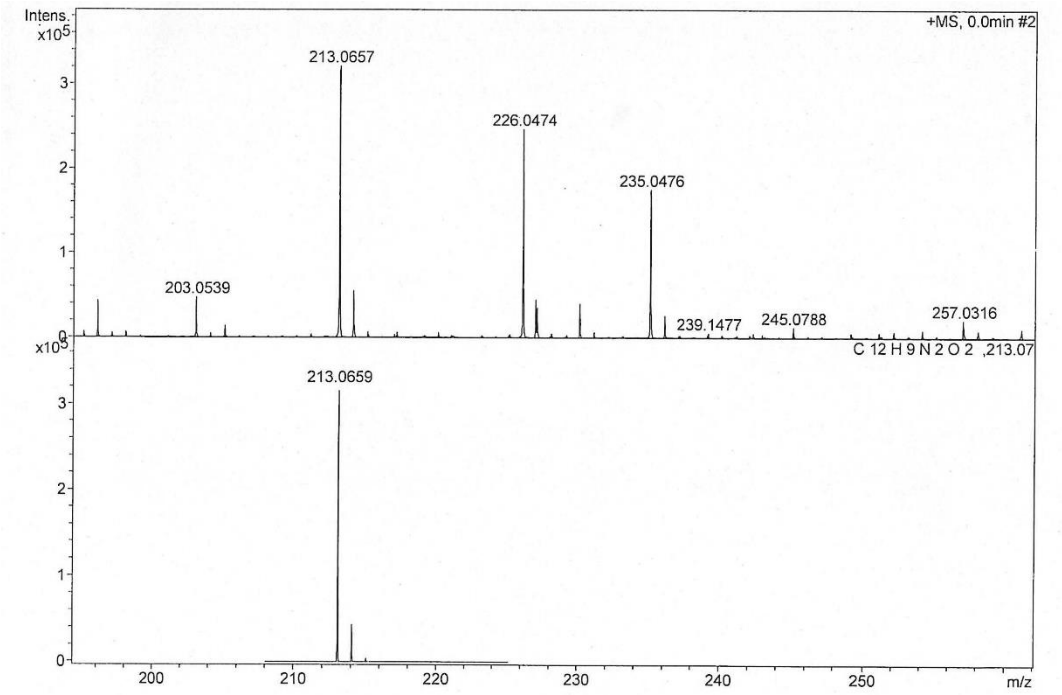

Figure S14 ^{1}H NMR spectrum of compound **3** in CD_3OD (400 MHz)

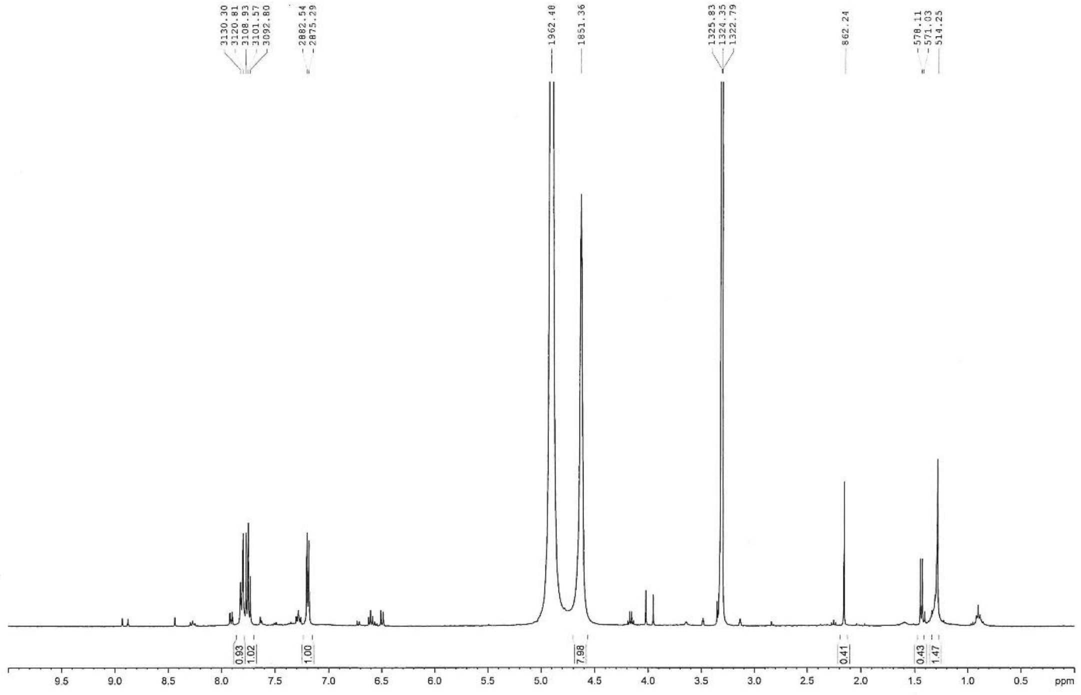

Figure S15 ^{13}C NMR spectrum of compound **3** in CD_3OD (100 MHz)

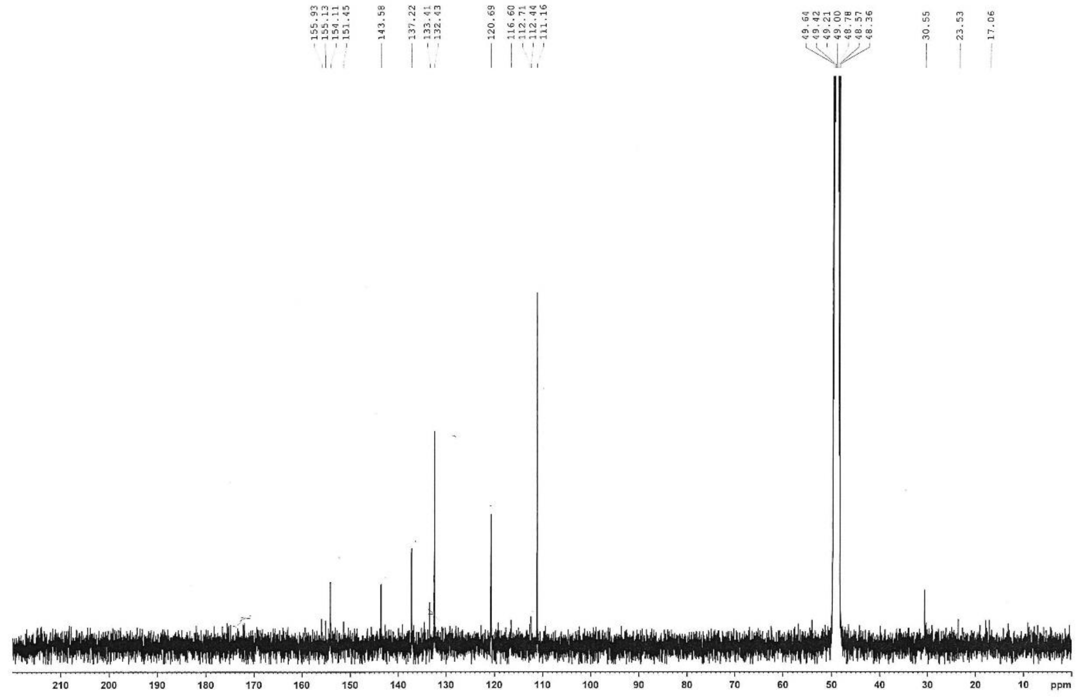

*Phenylacetic acid (compound **4**)*

Brown gum; ^{1}H-NMR (500 MHz, CD_3OD) 3.59 (2H, s), 7.23 (1H, m), 7.29 (4H, m); ^{13}C NMR (125 MHz, CD_3OD) 42.2 (CH_2), 128.0 (CH), 129.6 (CH), 130.5 (CH), 130.9 (C), 134.2 (C), 136.3 (C), 175.9 (C); HRESIMS *m/z* 159.0437 $[M+Na]^+$ (calcd for $C_8H_8O_2Na$, 159.0417).

Figure S16 HRESIMS of compound **4**

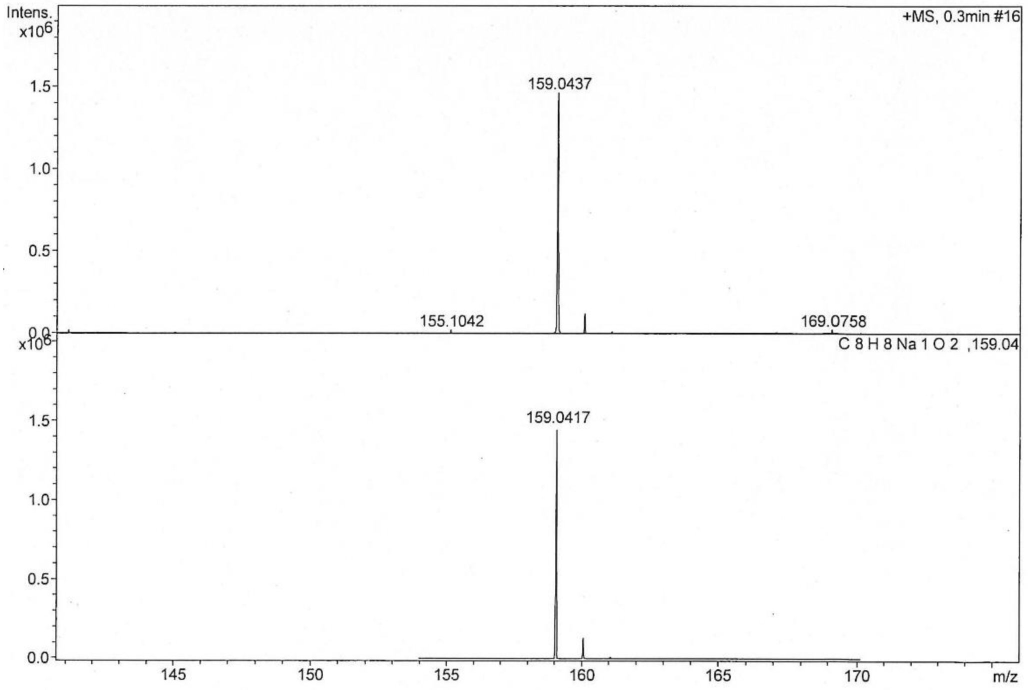

Figure S17 ^{1}H NMR spectrum of compound **4** in CD_3OD (500 MHz)

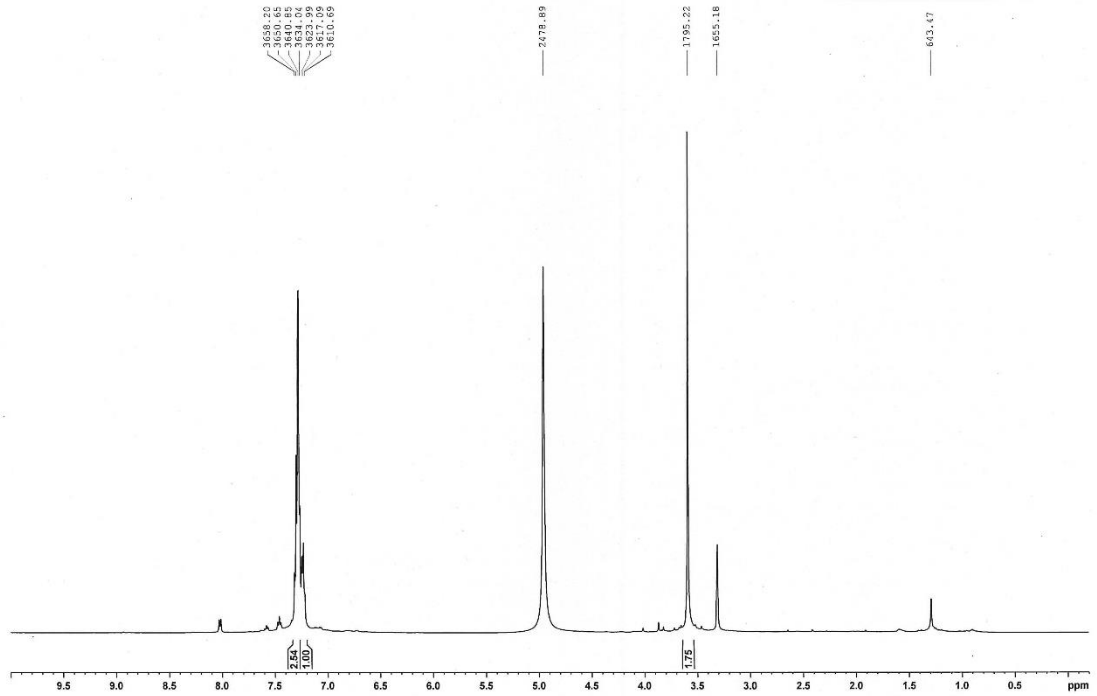

Figure S18 ^{13}C NMR spectrum of compound **4** in CD_3OD (125 MHz)

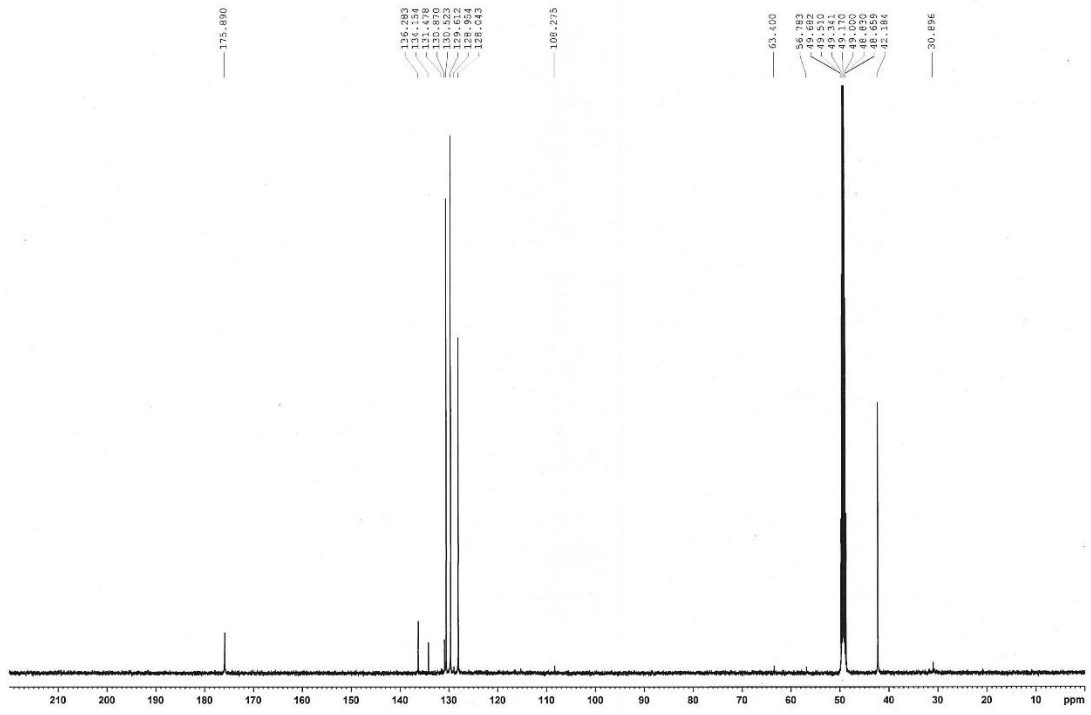

*p-Hydroxylphenylacetic acid (compound **5**)*

Yellow gum; ^{1}H-NMR (400 MHz, acetone-d_6) 3.50 (2H, s), 6.78 (2H, d, J = 8.35 Hz), 7.13 (2H, d, J = 8.30 Hz); ^{13}C NMR (100 MHz, acetone-d_6) 40.4 (CH_2), 115.9 (CH), 126.5 (C), 131.3 (CH), 157.1 (C), 173.2 (C); HRESIMS *m/z* 175.0373 $[M+Na]^+$ (calcd for $C_8H_8O_3Na$, 175.0366)

Figure S19 HRESIMS of compound **5**

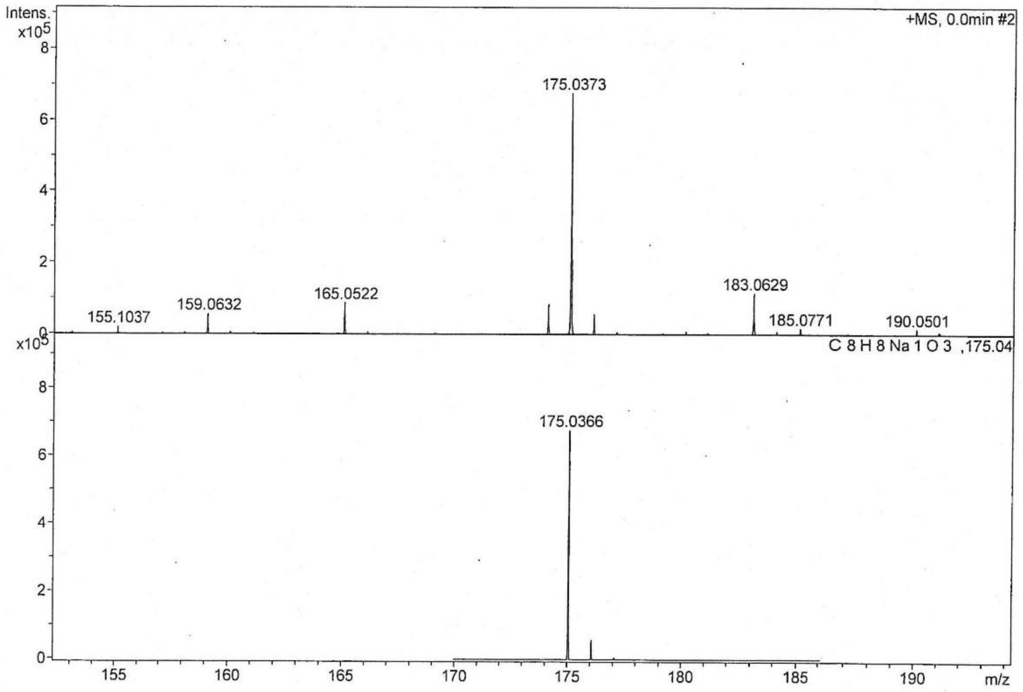

Figure S20 ^{1}H NMR spectrum of compound **5** in acetone-d_6 (400 MHz)

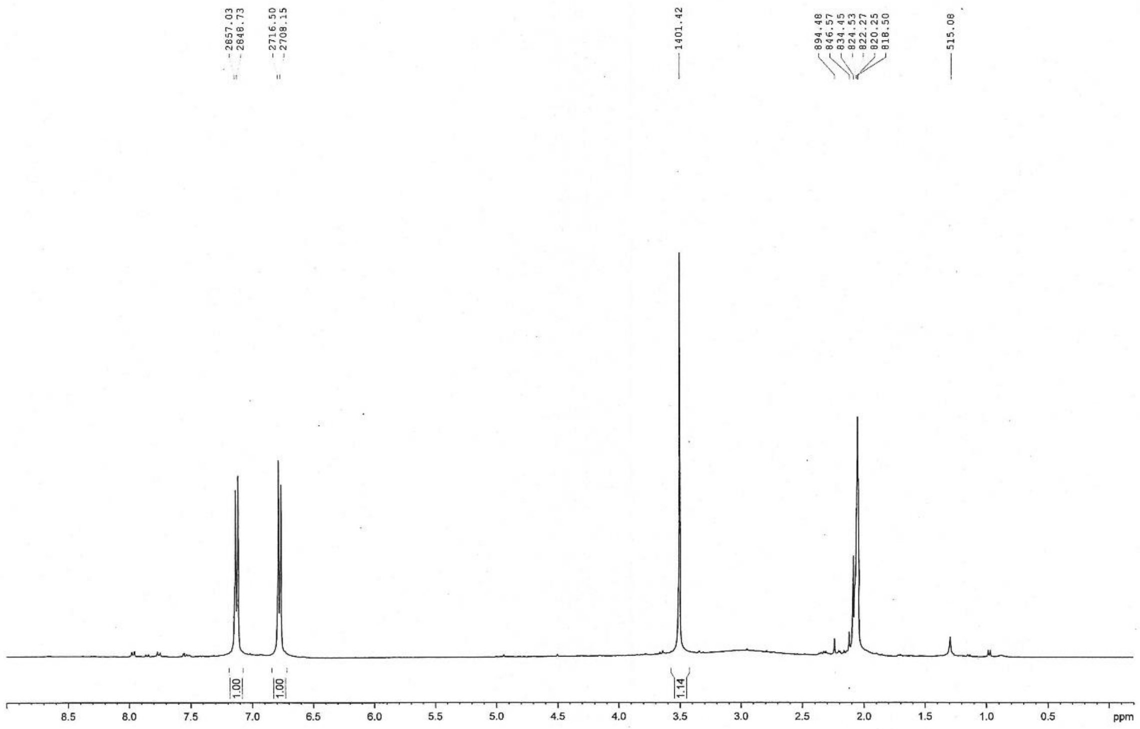

Figure S21 ^{13}C NMR spectrum of compound **5** in acetone-d_6 (100 MHz)

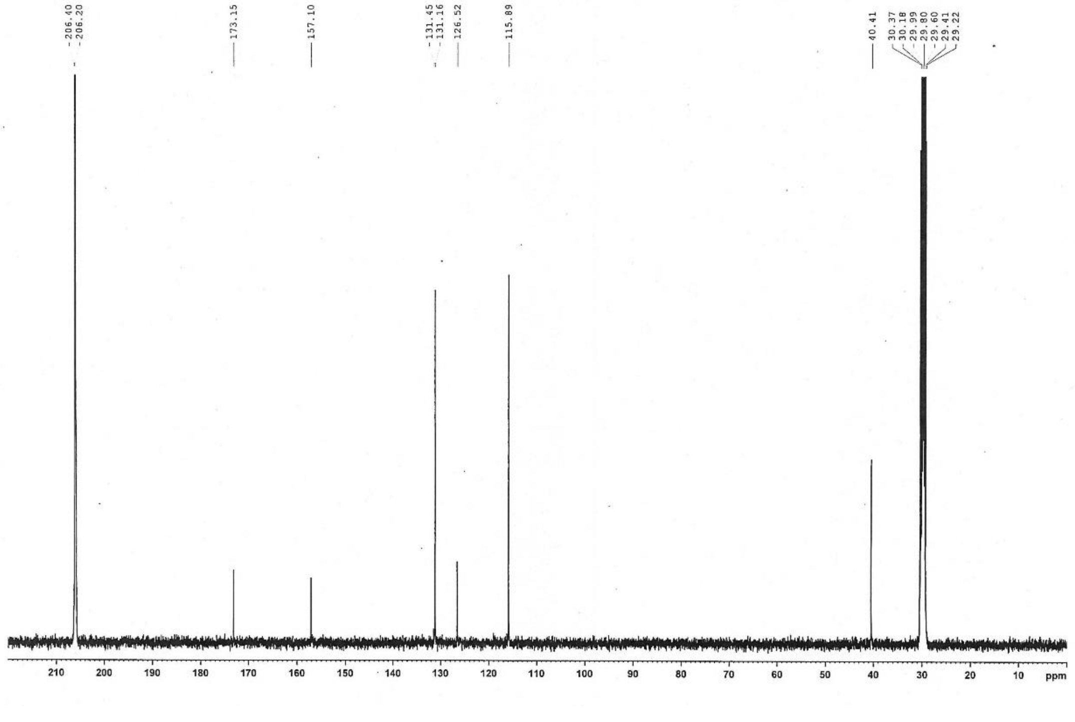

*Indole-3-carboxylic acid (compound **6**)*

Brown solid; ^{1}H-NMR (400 MHz, CD_3OD) 7.17 (1H, ddd, *J* =12.92, 6.46, 1.49 Hz), 7.20 (1H, ddd, *J* = 13.01, 6.51, 2.28 Hz), 7.44 (1H, dd, *J* = 7.02, 1.98 Hz), 7.95 (1H, s), 8.07 (1H, dd, *J* = 6.75, 2.03 Hz); ^{13}C NMR (100 MHz, CD_3OD) 109.1 (C), 112.8 (CH), 122.1 (CH), 122.3 (CH), 123.5 (CH), 127.7 (C), 133.3 (CH), 138.3 (C), 169.1 (C); HRESIMS *m/z* 184.0391 $[M+Na]^+$ (calcd for $C_9H_7NO_2Na$, 184.0369).

Figure S22 HRESIMS of compound **6**

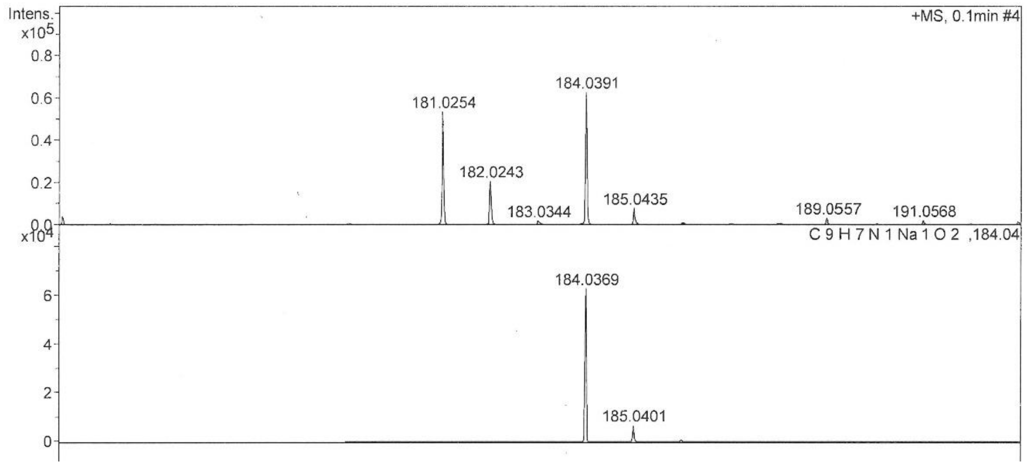

Figure S23 ^{1}H NMR spectrum of compound **6** in CD_3OD (400 MHz)

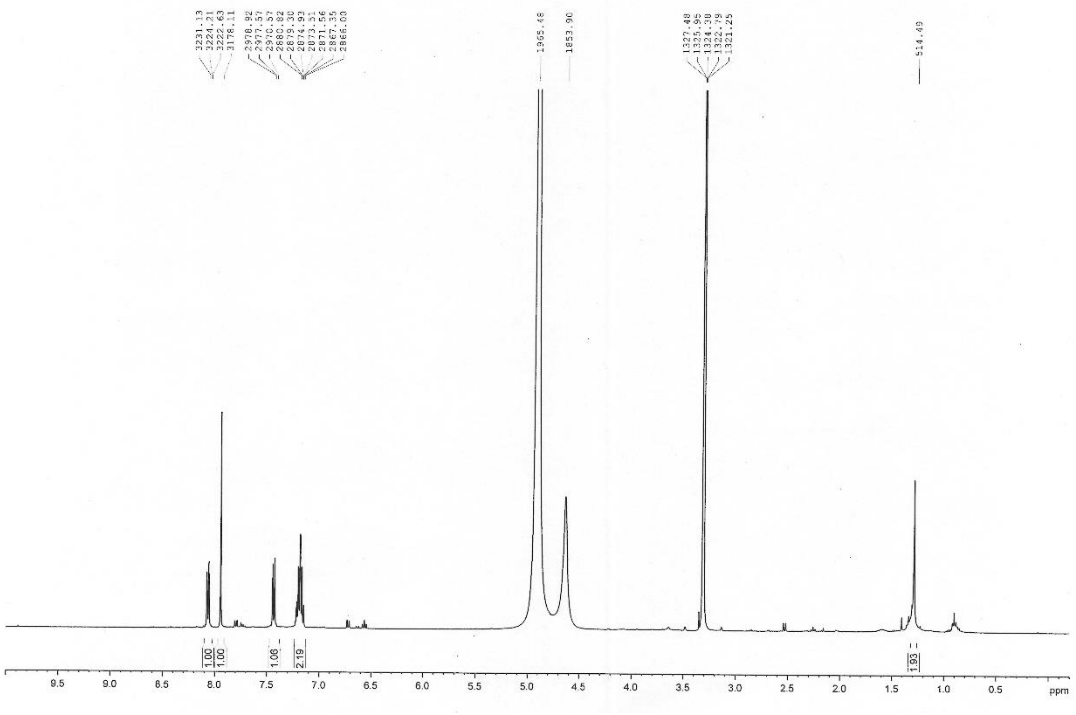

Figure S24 ^{13}C NMR spectrum of compound **6** in CD_3OD (100 MHz)

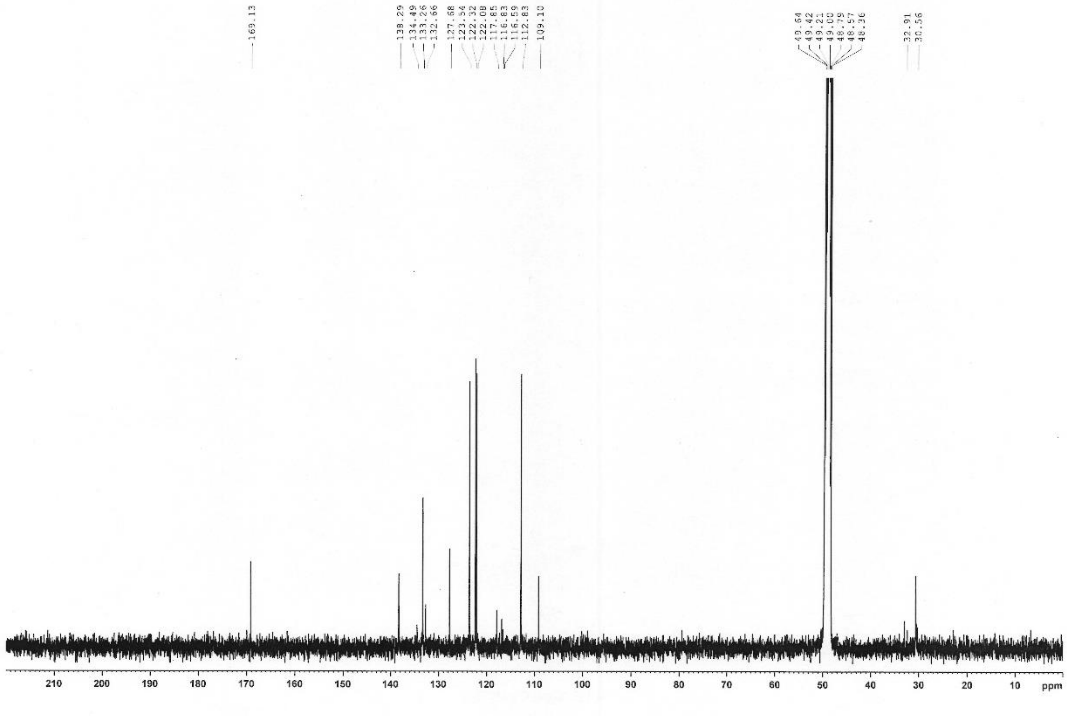

*Indole-3-acetic acid (compound **7**)*

Brown solid; ^{1}H-NMR (400 MHz, CD_3OD) 3.73 (2H, s), 7.01 (1H, t, J = 7.21 Hz), 7.10 (1H, t, J = 7.25 Hz), 7.16 (1H, s), 7.34 (1H, d, J = 8.11 Hz), 7.54 (1H, d, J = 7.91 Hz); ^{13}C NMR (100 MHz, CD_3OD) 32.0 (CH_2), 109.2 (C), 112.2 (CH), 119.4 (CH), 119.9 (CH), 122.5 (CH), 124.6 (CH), 128.8 (C), 138.1 (C), 176.2 (C); HRESIMS *m/z* 198.0524 $[M+Na]^+$ (calcd for $C_{10}H_9NO_2Na$, 198.0525).

Figure S25 HRESIMS of compound **7**

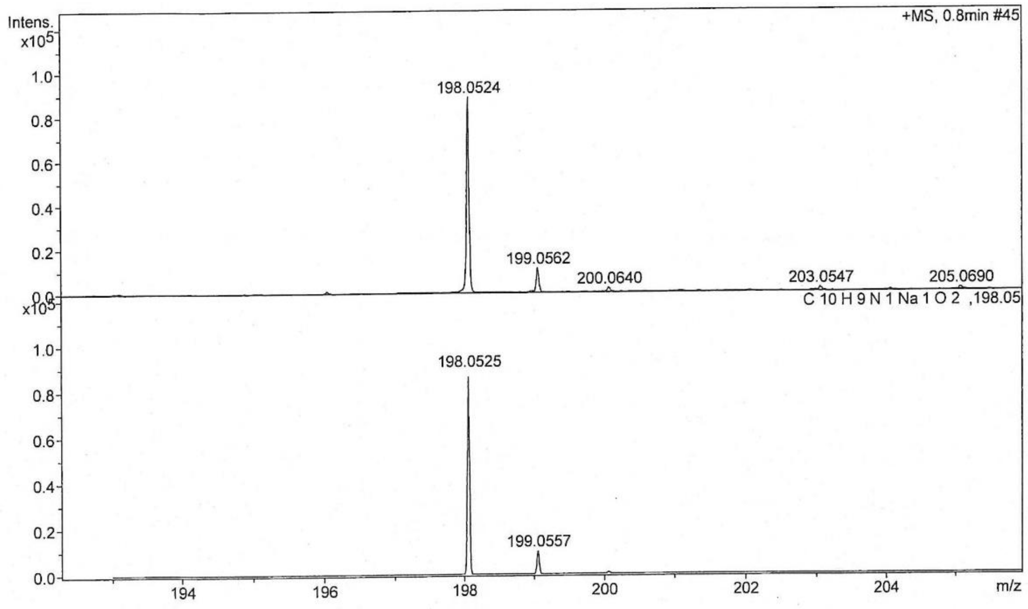

Figure S26 ^{1}H NMR spectrum of compound **7** in CD_3OD (400 MHz)

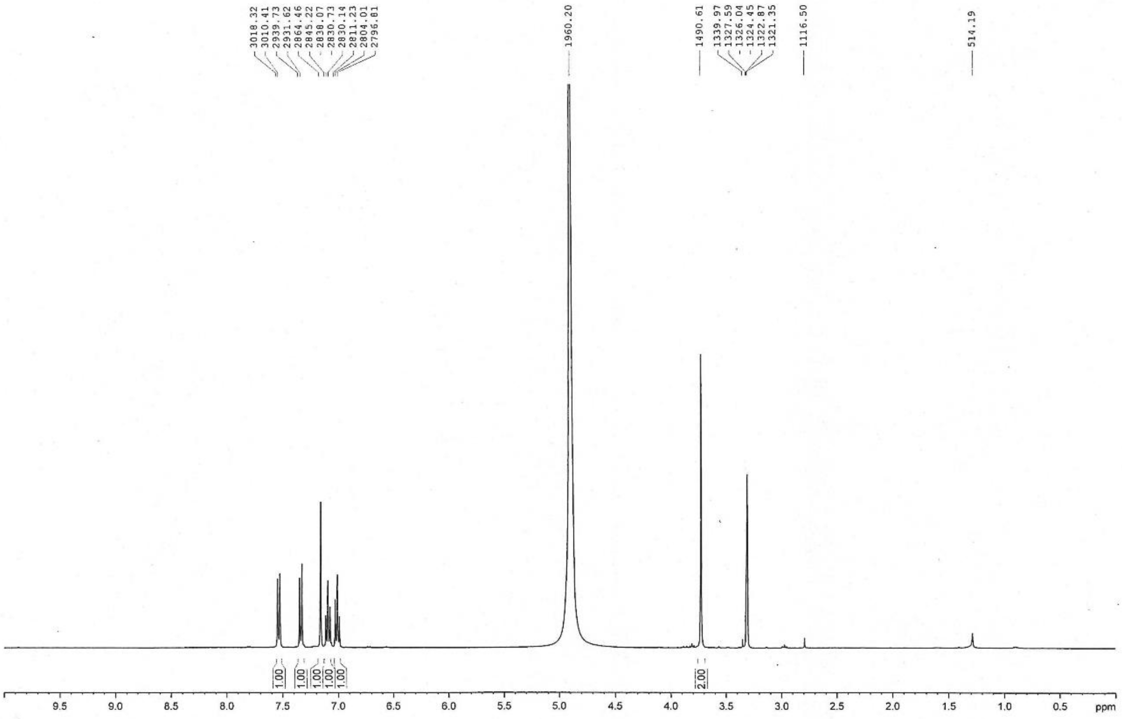

Figure S27 ^{13}C NMR spectrum of compound **7** in CD_3OD (100 MHz)

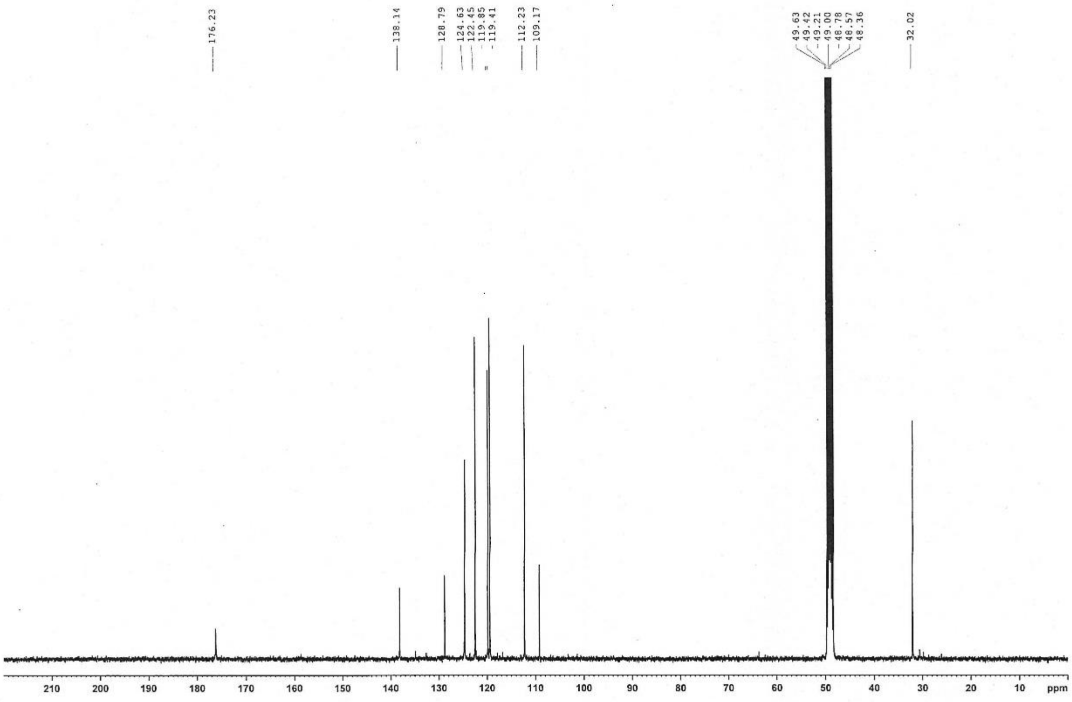

*Methyl indole-3-acetate (compound **8**)*

Brown gum; ^{1}H-NMR (500 MHz, CD_3OD) 3.68 (3H, s), 3.77 (2H, s), 7.01 (1H, t, *J* = 7.45 Hz), 7.10 (1H, t, *J* = 7.53 Hz), 7.16 (1H, s), 7.34 (1H, d, *J* = 8.11 Hz), 7.51 (1H, d, *J* = 7.91 Hz); ^{13}C-NMR (125 MHz, CD_3OD) 31.9 (CH_2), 52.4 (CH_3), 108.6 (C), 112.3 (CH), 119.3 (CH), 119.9 (CH), 122.5 (CH), 124.7 (CH), 128.6 (C), 138.0 (C), 174.9 (C); HRESIMS *m/z* 212.0684 $[M+Na]^+$ (calcd for $C_{11}H_{11}NO_2Na$, 212.0682).

Figure S28 HRESIMS of compound **8**

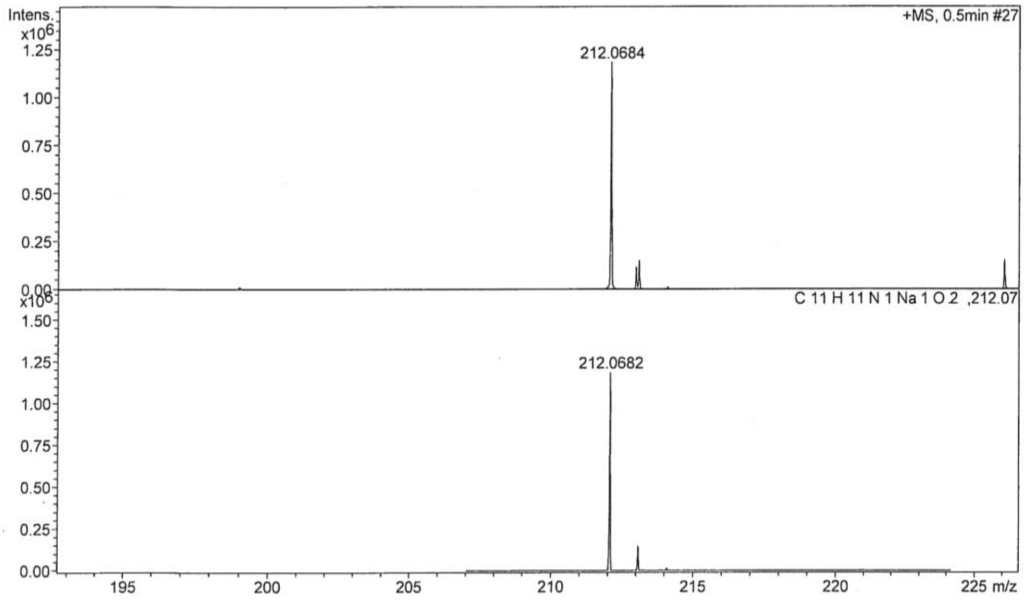

Figure S29 ^{1}H NMR spectrum of compound **8** in CD_3OD (500 MHz)

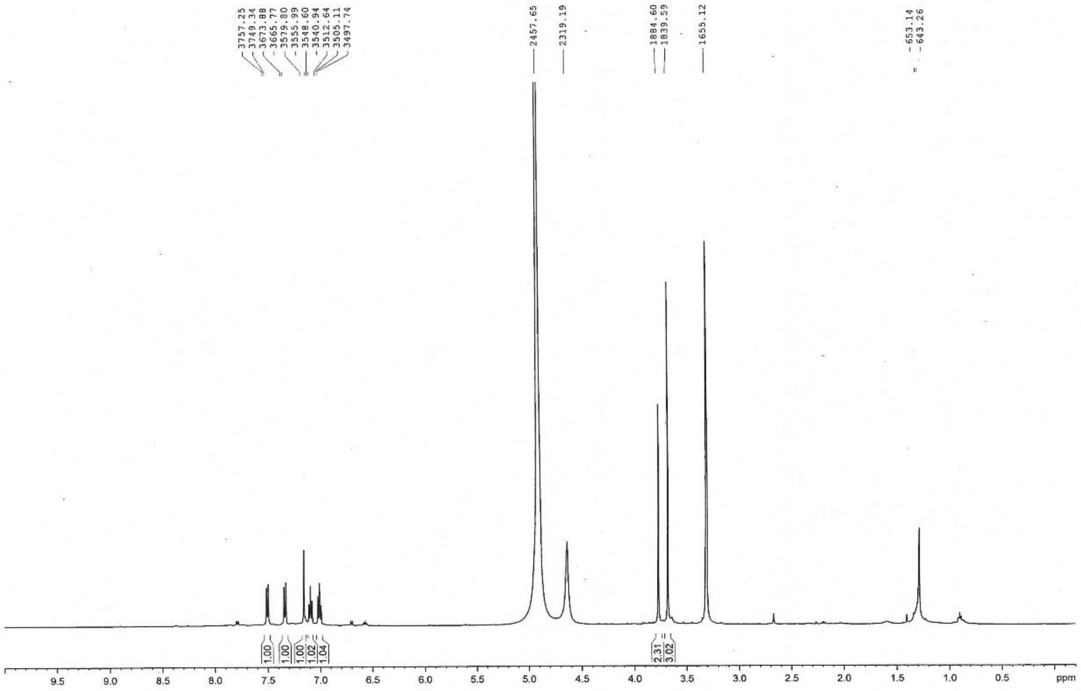

Figure S30 ^{13}C NMR spectrum of compound **8** in CD_3OD (125 MHz)

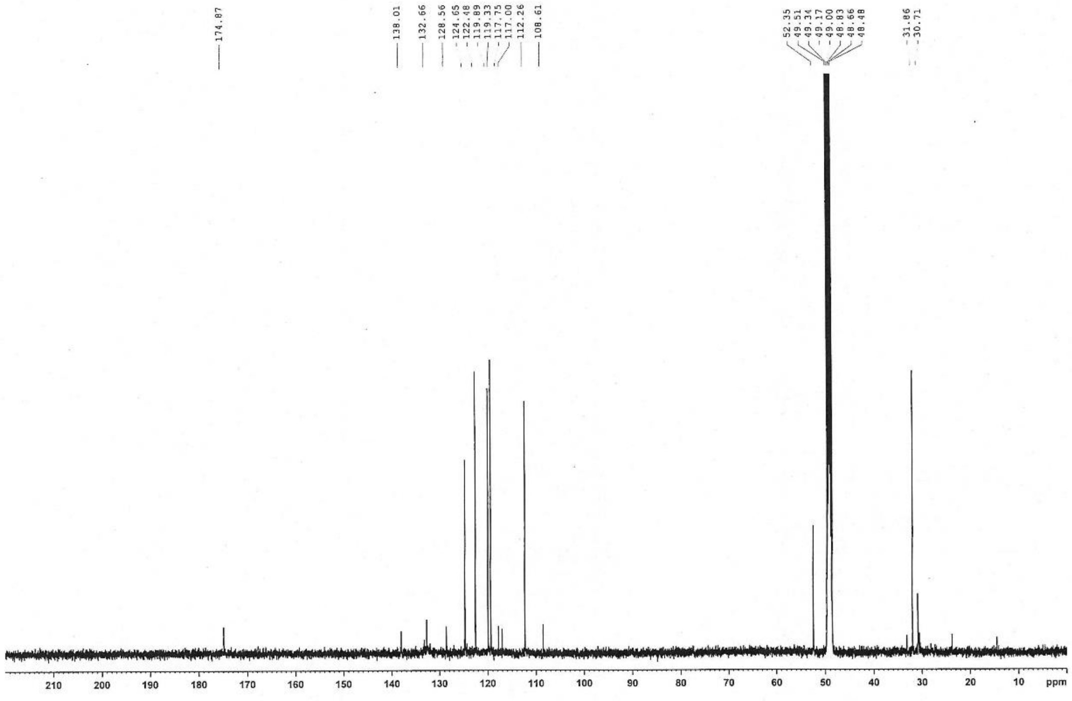

*Anthranillic acid (compound **9**)*

Brown solid; ^{1}H-NMR (400 MHz, CD_3OD) 6.57 (1H, ddd, J = 8.10, 7.02, 0.93 Hz), 6.73 (1H, dd, J = 8.48, 0.64 Hz), 7.22 (1H, ddd, J = 8.17, 7.21, 1.41 Hz), 7.80 (1H, dd, J = 8.04, 1.24 Hz); ^{13}C NMR (100 MHz, CD_3OD) 112.3 (C), 116.8 (CH), 117.9 (CH), 132.7 (CH), 134.9 (CH), 152.6 (C), 171.6 (C); HRESIMS *m/z* 136.0402 [M-H]$^-$ (calcd for $C_7H_6NO_2$, 136.0404).

Figure S31 HRESIMS of compound **9**

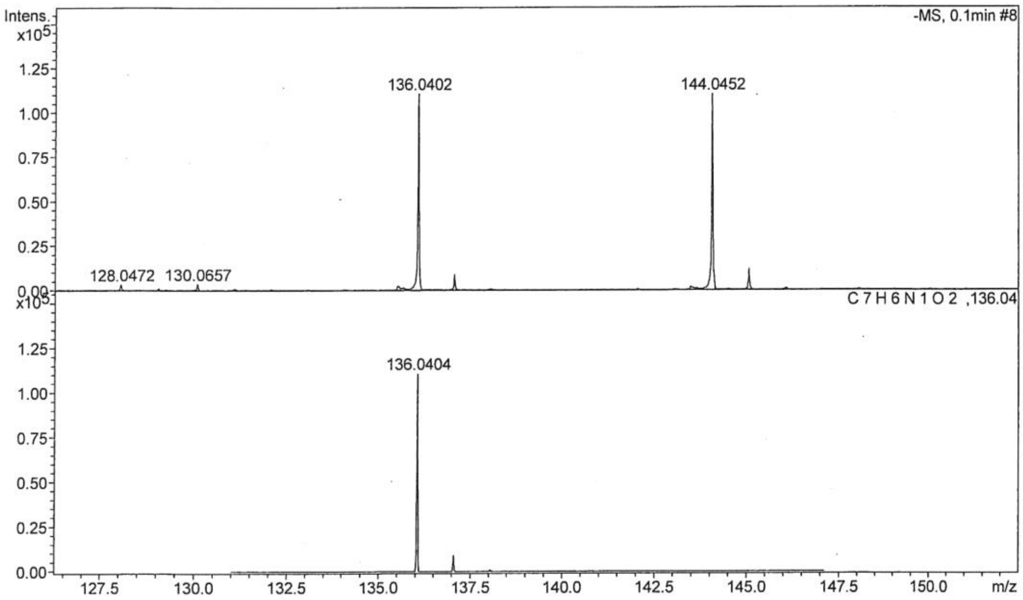

Figure S32 ^{1}H NMR spectrum of compound **9** in CD_3OD (400 MHz)

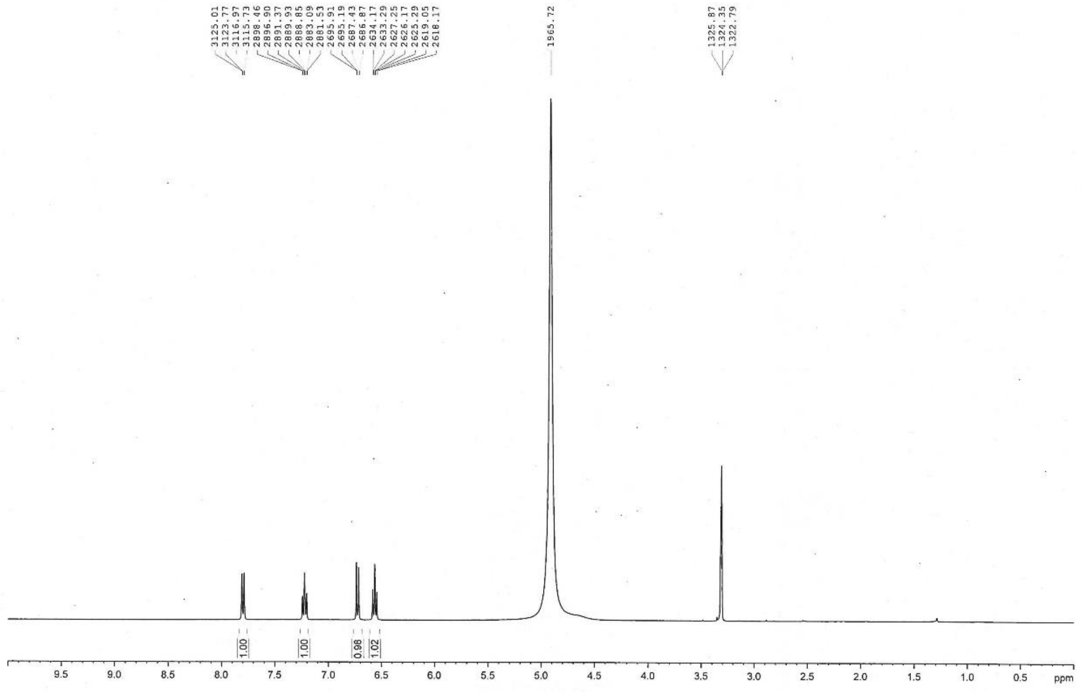

Figure S33 ^{13}C NMR spectrum of compound **9** in CD_3OD (100 MHz)

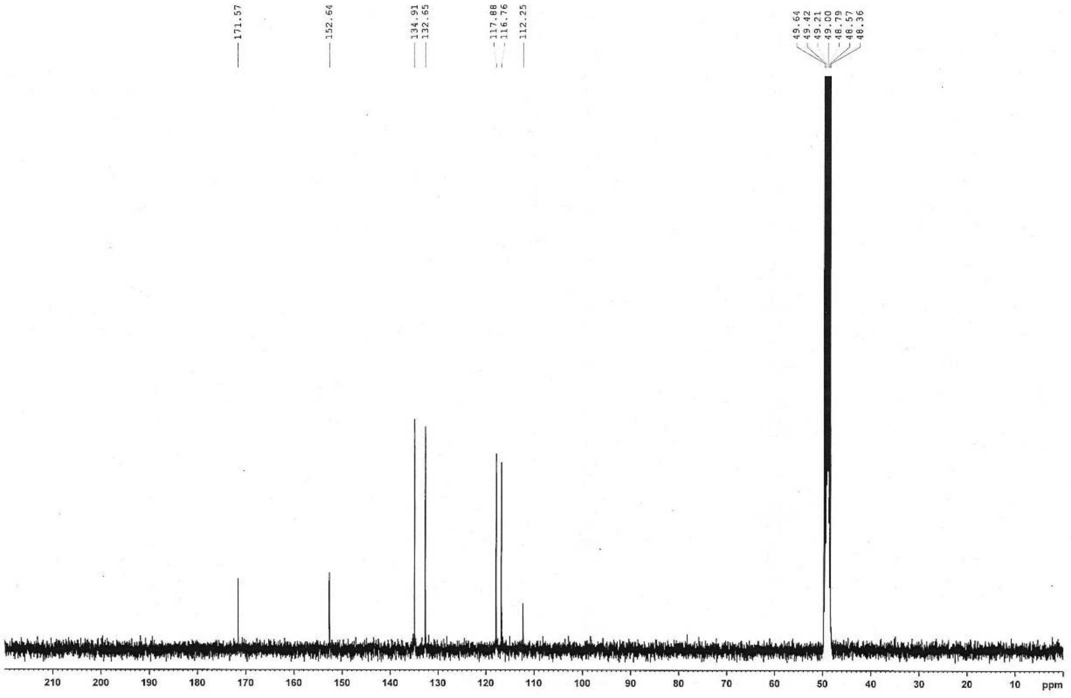

*3-Pyridinecarboxylic acid (compound **10**)*

Brown solid; ^{1}H-NMR (500 MHz, DMSO-d_6) 7.54 (1H, q, J = 7.83, 4.83 Hz), 8.26 (1H, td, J = 7.87, 1.81 Hz), 8.77 (1H, dd, J = 4.75, 1.45 Hz), 9.07 (1H, d, J = 1.65 Hz); ^{13}C-NMR (125 MHz, DMSO-d_6) 124.2 (CH), 127.5 (C), 137.4 (CH), 150.7 (CH), 153.6 (CH), 166.8 (C); HRESIMS *m/z* 146.0304 [M+Na]$^+$ (calcd for $C_6H_5NO_2Na$, 146.0212).

Figure S34 HRESIMS of compound **10**

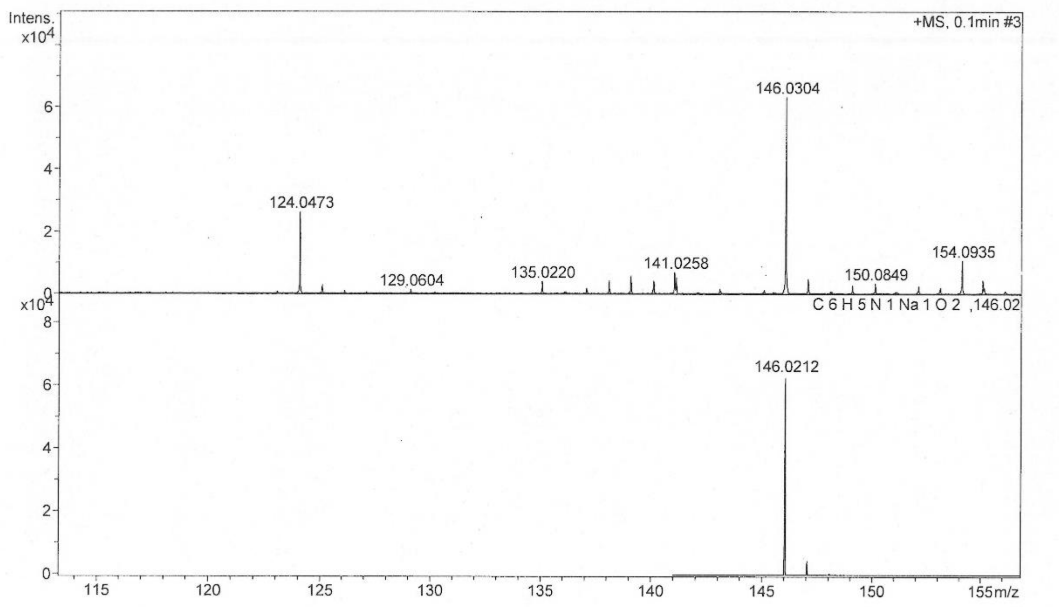

Figure S35 ^{1}H NMR spectrum of compound **10** in DMSO-d_6 (500 MHz)

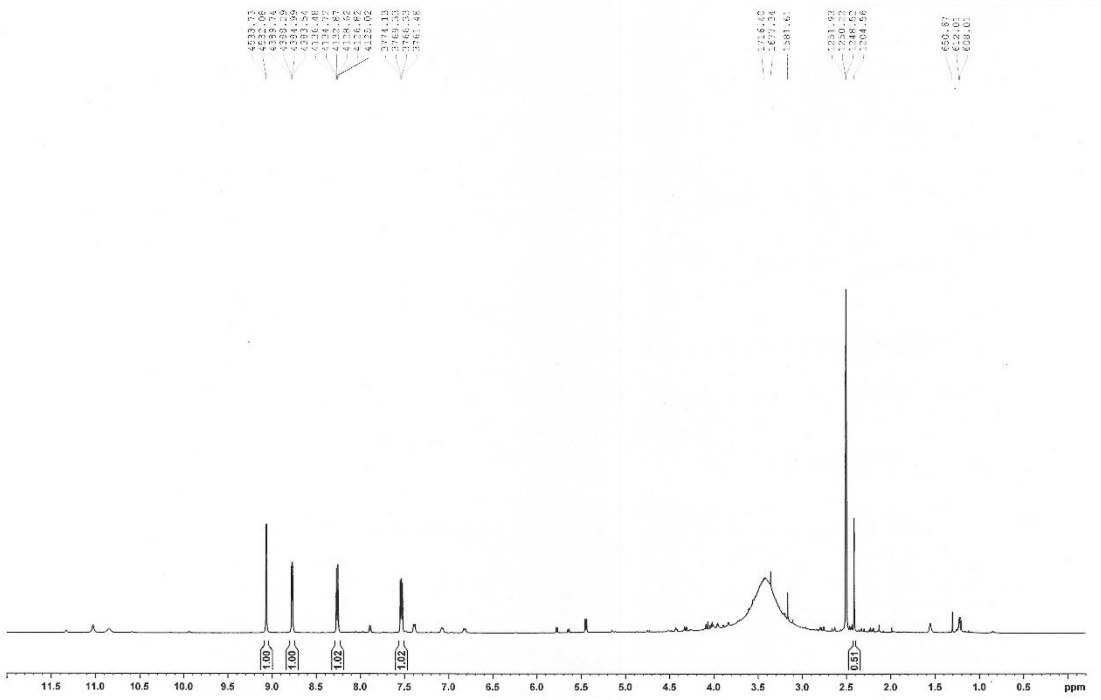

Figure S36 ^{13}C NMR spectrum of compound **10** in DMSO-d_6 (125 MHz)

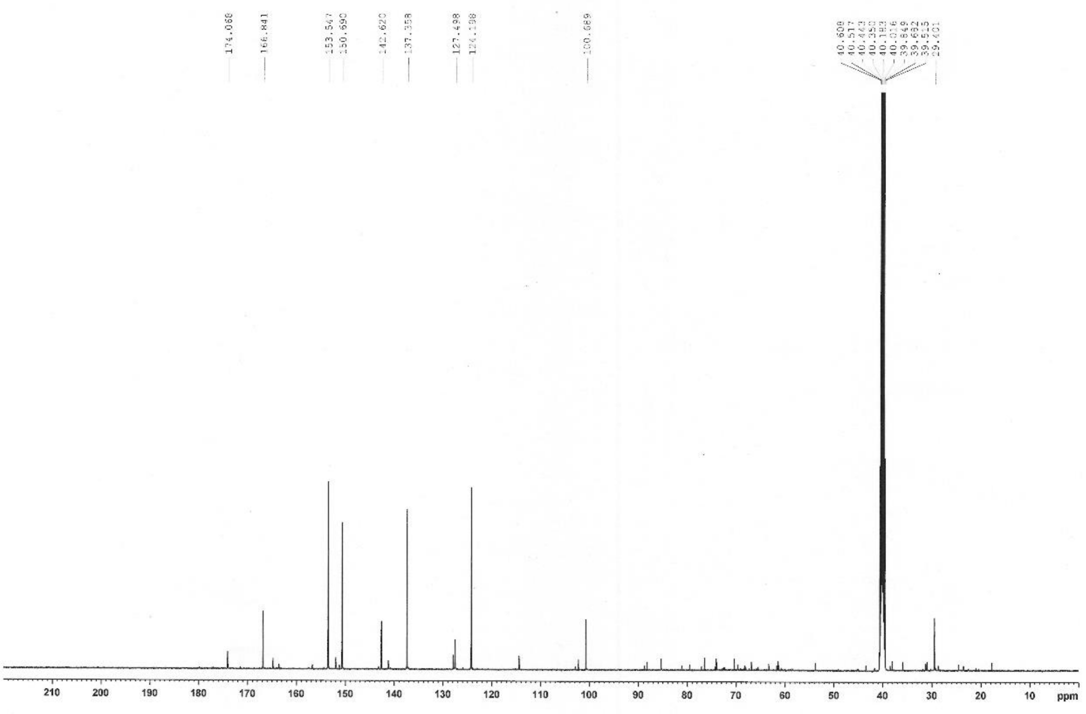

Figure S37. Viability of P19-derived neuron treated with the compound 1 at various concentrations of 1-10000 ng/ml. The error bar represented standard error of the mean (SE). The 0.5%DMSO in the medium was used as a control representing no effects on the neuronal viability (99.87 ± 0.13% neuron viability) of the solvent.

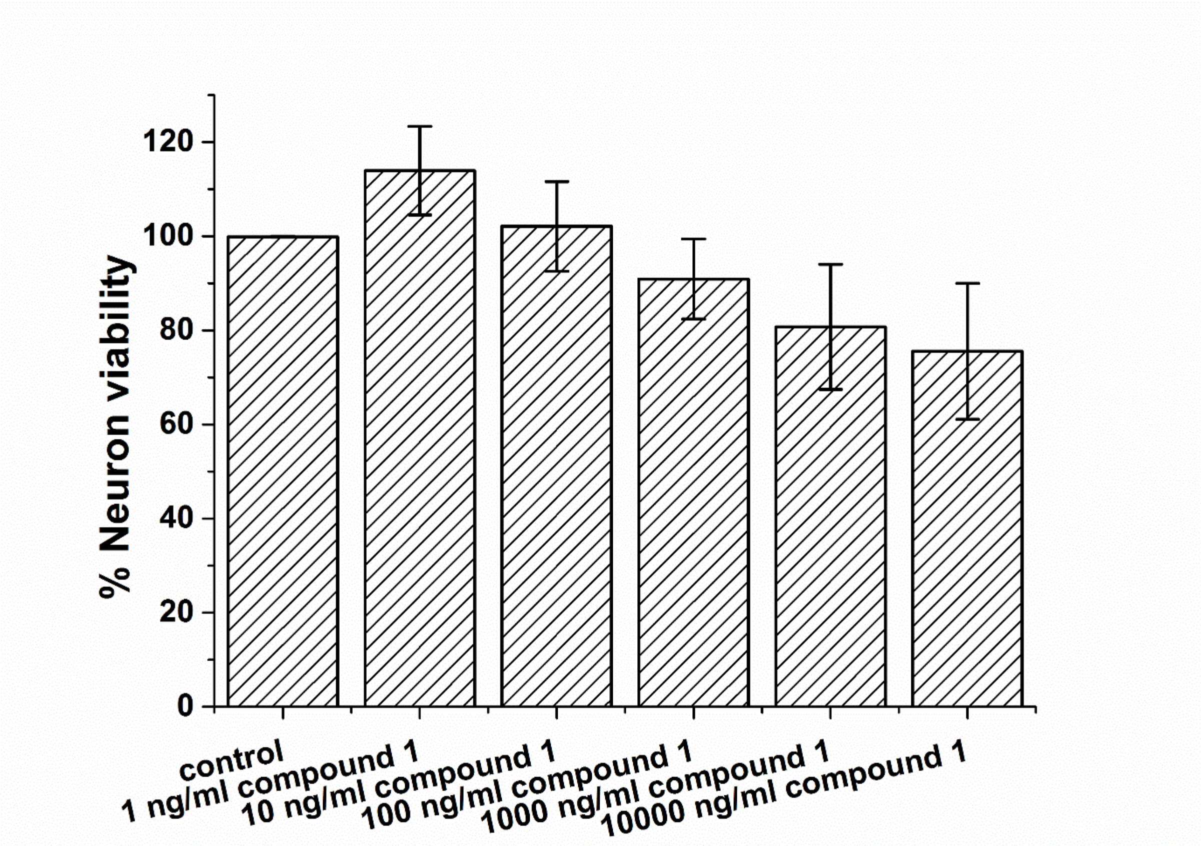

Figure S38. *In silico* ADME prediction of compound 1 using the SwissADME web-based application (http://www.swissadme.ch).

Supplementary Figure

In silico ADME prediction of compound 1 using the SwissADME web-based application (http://www.swissadme.ch).

Hide BOILED-Egg

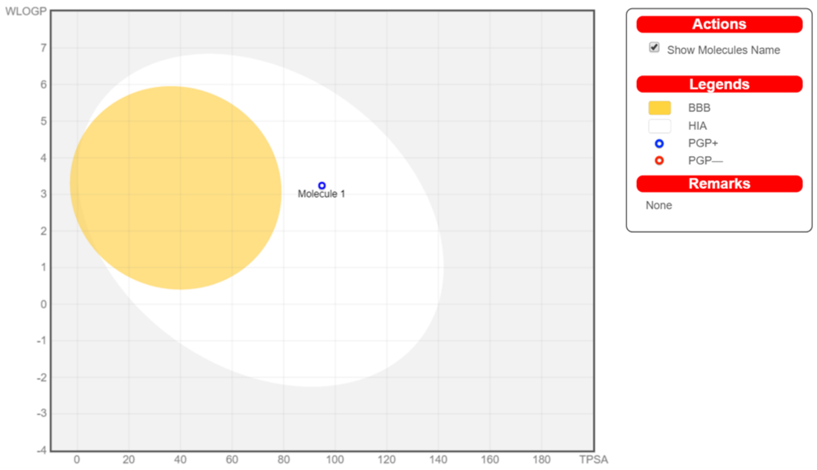

Molecule 1

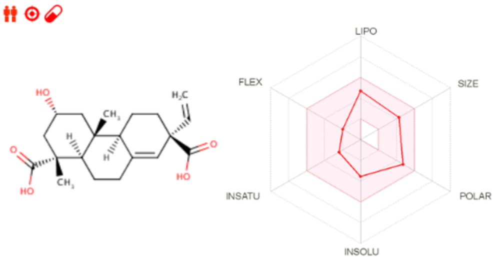

SMILES C=C[C@]1(CC[C@H]2C(=C1)CC[C@@H]1[C@]2(C)C[C@@H](O)C[C@@]1(C)C(=O)O)C(=O)O

Physicochemical Properties	
Formula	C20H28O5
Molecular weight	348.43 g/mol
Num. heavy atoms	25
Num. arom. heavy atoms	0
Fraction Csp3	0.70
Num. rotatable bonds	3
Num. H-bond acceptors	5
Num. H-bond donors	3
Molar Refractivity	94.89
TPSA	94.83 Å²

Lipophilicity	
Log $P_{o/w}$ (iLOGP)	1.69
Log $P_{o/w}$ (XLOGP3)	2.74
Log $P_{o/w}$ (WLOGP)	3.24
Log $P_{o/w}$ (MLOGP)	2.70
Log $P_{o/w}$ (SILICOS-IT)	2.48
Consensus Log $P_{o/w}$	2.57

Water Solubility	
Log *S* (ESOL)	-3.53
Solubility	1.03e-01 mg/ml ; 2.96e-04 mol/l
Class	Soluble
Log *S* (Ali)	-4.39
Solubility	1.43e-02 mg/ml ; 4.11e-05 mol/l
Class	Moderately soluble
Log *S* (SILICOS-IT)	-2.15
Solubility	2.49e+00 mg/ml ; 7.14e-03 mol/l
Class	Soluble

Pharmacokinetics	
GI absorption	High
BBB permeant	No
P-gp substrate	Yes
CYP1A2 inhibitor	No
CYP2C19 inhibitor	No
CYP2C9 inhibitor	No
CYP2D6 inhibitor	No
CYP3A4 inhibitor	No
Log K_p (skin permeation)	-6.48 cm/s

Druglikeness	
Lipinski	Yes; 0 violation
Ghose	Yes
Veber	Yes
Egan	Yes
Muegge	Yes
Bioavailability Score	0.56

Medicinal Chemistry	
PAINS	0 alert
Brenk	1 alert: isolated_alkene
Leadlikeness	Yes
Synthetic accessibility	5.17

Figure S39. A culture collection certificate of CSR-4

BIOTEC
a member of NSTDA

Thailand Bioresource Research Center (TBRC)
National Center for Genetic Engineering and Biotechnology
Innovation Cluster 2 (Tower B, 8th Floor, Room 817)
143 Thailand Science Park, Phahonyothin Road
Khlong Nueng, Khlong Luang, Pathum Thani 12120, Thailand
Tel +66-2-1178000-1, Fax: +66-2-1178003

ใบรับรองการฝาก

CERTIFICATE OF DEPOSIT

ใบรับรองเลขที่ / Certificate number: 2019-032   วันที่ออกใบรับรอง / Date of issue: 2 May 2019

ชื่อผู้ฝาก / Depositor: Assoc. Prof. Dr. Chitti Thawai

หน่วยงานและที่อยู่ / Institute and address: Department of Biology, Faculty of Science,
King Mongkut's Institute of Technology Ladkrabang
Bangkok 10520, Thailand

ขอรับรองว่าชีววัสดุที่มีรายชื่อต่อท้ายนี้ได้นำมาฝากเก็บรักษาและดูแล ณ Thailand Bioresource Research Center (TBRC) และได้ให้เลขที่รหัสประจำตัวชีววัสดุไว้เป็นหลักฐานการรับฝาก

This is to certify that the following biological material(s) has/have been maintained at Thailand Bioresource Research Center (TBRC). Accession number(s) of the biological material(s) is/are the evidence of deposition.

รายชื่อชีววัสดุ / List of biological materials

ชื่อชีววัสดุ<br>Biological material name	รหัสชีววัสดุ<br>Accession number	ระยะเวลาการฝาก<br>Deposit period	หมายเหตุ*<br>Remark
Microbispora sp., CSR-4	TBRC 10616	Since 7 February 2019	Remark 2

* Remark 1 = TBRC จัดตัวอย่างดังกล่าวอยู่ในส่วนที่เผยแพร่แก่สาธารณะได้ ข้อมูลตัวอย่างจะเผยแพร่ในแค็ตตาล็อกหลังจาก TBRC ออกใบรับรองการฝาก
The biological material is available in the publicly accessible section of the TBRC. The biological material will be included in the online catalog after TBRC issues a certificate of deposit.

Remark 2 = TBRC จัดตัวอย่างดังกล่าวอยู่ในส่วนที่เผยแพร่แก่สาธารณะได้ และไม่ได้วางข้อกำหนดในการเข้าถึงข้อมูลเรื่องการมีอยู่ของตัวอย่างดังกล่าว ณ TBRC ข้อมูลตัวอย่างจะเผยแพร่ในแค็ตตาล็อกหลังจากได้รับการตีพิมพ์
The biological material is available in the publicly accessible section of the TBRC and restrictions have not been placed on access to information concerning the presence of the biological material in the TBRC. The biological material will be included in the online catalog after publication.

Remark 3 = ไม่เผยแพร่แก่สาธารณะ
The material is not available for public access.

ลายเซ็น / Signature Chanwit Suriyachadkun

ผู้รับฝาก / Curator Chanwit Suriyachadkun

F-BT-TBRC-05 Rev.3
